# Design, Synthesis, and Molecular Evaluation of S_N_Ar‐Reactive *N*‐(6‐Fluoro‐3‐Nitropyridin‐2‐yl)Isoquinolin‐3‐Amines as Covalent USP7 Inhibitors Reveals an Unconventional Binding Mode

**DOI:** 10.1002/ardp.70053

**Published:** 2025-08-10

**Authors:** Larissa N. Ernst, Jason Stahlecker, Finn Mier, Ricardo A. M. Serafim, Valentin R. Wydra, Benedikt Masberg, Simon J. Jaag, Cornelius Knappe, Michael Lämmerhofer, Thilo Stehle, Matthias Gehringer, Frank M. Boeckler

**Affiliations:** ^1^ Department of Pharmacy and Biochemistry, Laboratory for Molecular Design & Pharmaceutical Biophysics, Institute of Pharmaceutical Sciences Eberhard Karls Universität Tübingen Tübingen Germany; ^2^ Department of Medicinal Chemistry, Faculty of Medicine, Institute for Biomedical Engineering Eberhard Karls Universität Tübingen Tübingen Germany; ^3^ Department of Pharmacy and Biochemistry, Pharmaceutical Chemistry, Institute of Pharmaceutical Sciences Eberhard Karls Universität Tübingen Germany; ^4^ Department of Pharmacy and Biochemistry, Pharmaceutical (Bio‐)Analysis, Institute of Pharmaceutical Sciences Eberhard Karls Universität Tübingen Germany; ^5^ Interfaculty Institute of Biochemistry Eberhard Karls Universität Tübingen Tübingen Germany; ^6^ Cluster of Excellence iFIT (EXC 2180) ‘Image‐Guided & Functionally Instructed Tumor Therapies’ Eberhard Karls Universität Tübingen Tübingen Germany; ^7^ Interfaculty Institute for Biomedical Informatics (IBMI) Tübingen Germany

**Keywords:** covalent inhibitor, differential scanning fluorimetry (DSF), intact protein mass spectrometry, USP7, X‐ray crystallography

## Abstract

The cysteine protease ubiquitin‐specific protease 7 (USP7), also known as herpes‐associated ubiquitin‐specific protease (HAUSP), has gained increasing attention in recent years due to its proven overexpression in several cancer types and its role in tumorigenesis. Herein, after a design based on molecular docking experiments, we report the synthesis of a series of mildly electrophilic compounds that covalently modify the catalytic cysteine 223 in USP7 through a nucleophilic aromatic substitution (S_N_Ar) reaction. The compounds were first evaluated using differential scanning fluorimetry (DSF) to describe their influence on the melting temperature of native and mutant USP7 variants. Furthermore, the possible formation of a covalent bond was analyzed using intact protein mass spectrometry (MS). For promising derivatives, IC_50_ values were determined in an enzyme activity assay to confirm an inhibitory effect on USP7. Finally, a co‐crystal structure revealed that the prototype compound (**7a**) arylates the catalytic cysteine in the apo form of USP7 via an unconventional binding mode near the catalytic triad. The synthesis and biological evaluation of this compound series provides valuable structure–activity relationships (SAR) and reveals an interesting and unprecedented binding mode, thus providing a basis for improving USP7 inhibitors.

## Graphical abstract

The ubiquitin‐specific protease 7 (USP7) catalyzes the removal of ubiquitin and protects substrate proteins from degradation. USP7 causes structural changes upon ubiquitin binding, making it particularly suitable for use in covalent targeting approaches. The development, synthesis, and biophysical screening of a series of mildly electrophilic compounds reveals an unconventional covalent binding mode in a co‐crystal structure of USP7.

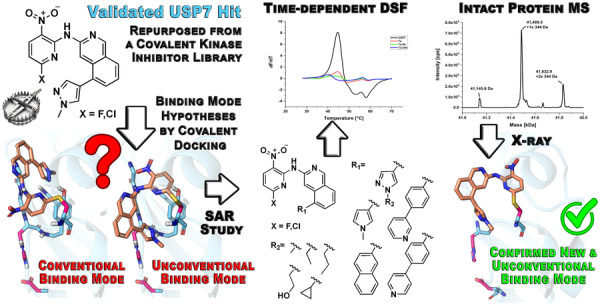

## 1 Introduction

In recent years, the cysteine protease ubiquitin‐specific protease 7 (USP7), also known as herpes‐associated ubiquitin‐specific protease (HAUSP), has become one of the most extensively studied deubiquitinases [1, 2]. This is mainly due to its role in tumorigenesis‐associated signaling pathways [3, 4, 5, 6] and its interaction with the tumor suppressor p53 and the oncogenic ubiquitin E3 ligase murine double minute (MDM2) [7, 8, 9].

Recently, several small molecules targeting USP7 either covalently [10, 11, 12, 13, 14, 15], like 4‐hydroxypiperidine FT827 with a vinyl sulfonamide warhead [10] and *N*‐cyanopyrrolidine derivatives [16], or non‐covalently [10, 17, 18, 19, 20], like quinazolin‐4‐one derivatives [21], have been identified. Other opportunities to target USP7 are allosteric inhibitors like 2‐amino‐4‐ethylpyridine derivatives GNE‐6640 and GNE‐6776 [20, 22] or proteolysis targeting chimera (PROTAC) degraders such as U7D‐1 [23] and CST967 [24]. Notably, none of these compounds have yet advanced to clinical trials [15, 25]. New approaches with a greater diversity of chemotypes, including different ways of targeting the binding site, as well as the use of alternative covalent mechanisms, may increase the selectivity and inhibitory potency on USP7 and provide a basis for clinical translation.

Covalent inhibitors have gained increasing popularity over the last few years since it was shown that the benefits of a well‐designed covalent inhibitor outweigh the risks, even in the case of irreversible covalent modes of action [26]. These inhibitors can offer various advantages like increased potency and prolonged target occupancy. Consequently, this may translate into improved therapeutic efficacy but also an improved selectivity if a poorly conserved amino acid is specifically addressed through the covalent‐reactive motif, often referred to as the covalent “warhead.” Moreover, the formation of an irreversible covalent bond enables the detection of covalent protein adducts by intact protein mass spectrometry (MS), which facilitates hit identification by screening covalent libraries containing reactive fragments or lead‐like compounds [27]. While most targeted covalent inhibitors (TCIs) are currently relying on α,β‐unsaturated amide electrophiles addressing cysteine (i.e., the most nucleophilic proteinogenic amino acid), an extension of the repertoire of covalent warheads is of major importance [28, 29].

Therefore, we have previously screened covalent libraries which included several nonconventional warhead types against USP7 and other target proteins [30, 31]. The focus of this study was the identification and validation of new chemotypes engaging USP7 by reacting with its nucleophilic catalytic cysteine residue (Cys233). The first approach was the screening of a library containing reactive fragments with different electrophilic warheads (CovLib). This screen mostly identified compounds that reacted covalently with the catalytic domain of USP7 in a nucleophilic aromatic substitution reaction (S_N_Ar) [31]. In a second, bigger screen employing a library of compounds which had been designed as covalent protein kinase inhibitors [30], S_N_Ar‐based electrophilic warheads again proved to be most suitable to address the catalytic cysteine. From this recent screen, hit compound **7a** emerged showing a temperature decrease of –4°C with USP7 in a differential scanning fluorimetry (DSF) experiment, indicating binding and a concurrent destabilization of the target protein. The covalent binding mode was confirmed by intact protein MS. Covalent docking of **7a** into the inactive USP7 protein proposed two binding modes, one with the ligand pointing to the commonly observed direction in which the ubiquitin binds and a preferred, more unusual orientation toward the catalytic triad's histidine and aspartate. These results served as a starting point for the design and synthesis of a series of possible USP7 inhibitors bearing a S_N_Ar warhead with either fluoride or, for a more tempered reactivity, chloride as a leaving group. While DSF and intact protein MS were employed for an initial assessment, hit compounds were further validated in an enzymatic assay to confirm the inhibitory effect on USP7. Ultimately, prototype compound **7a** was co‐crystalized and the crystal structure confirmed an unconventional binding mode near the catalytic triad of USP7, providing an important basis for future rational design efforts.

## 2 Results and Discussion

### 2.1 Molecular Docking

**Figure 1:**
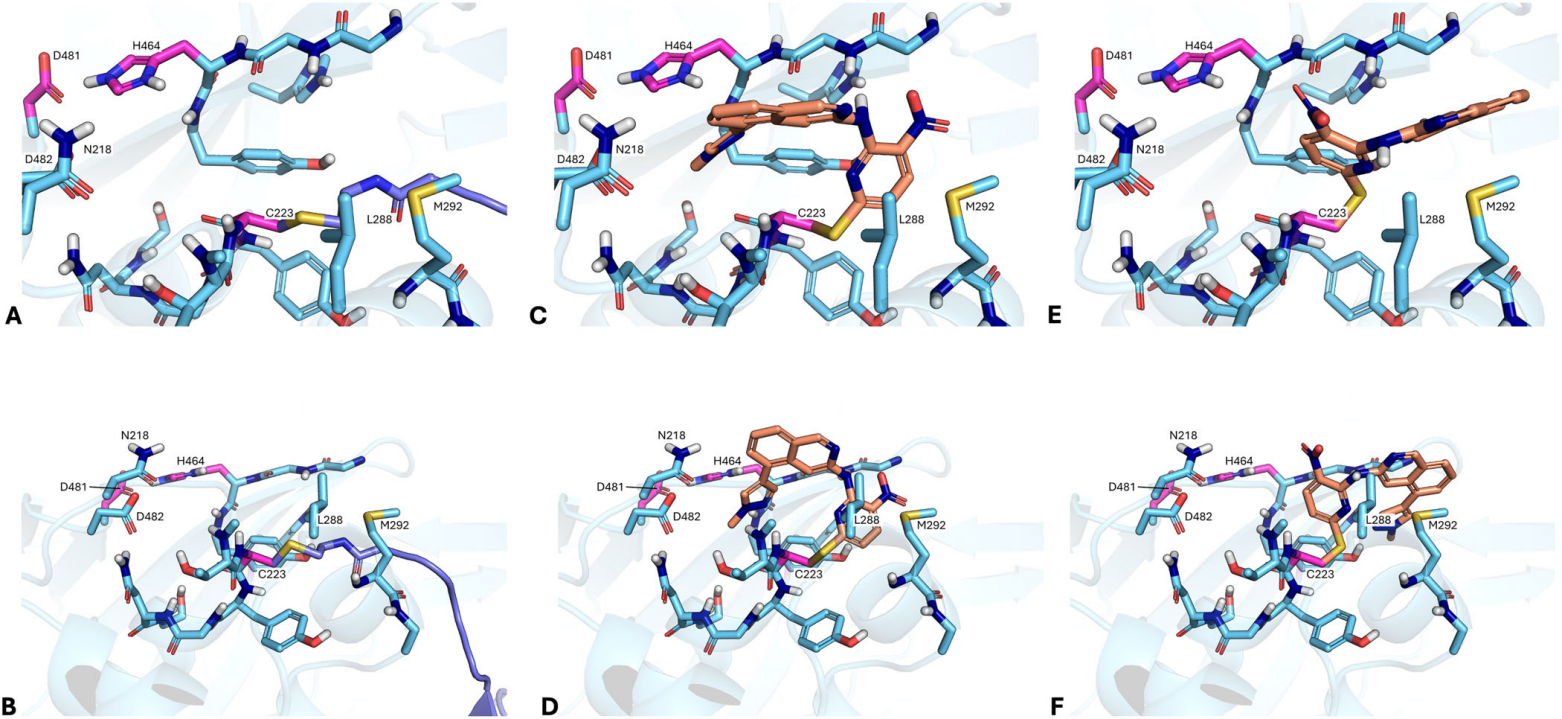
Overview of the docking results: Amino acids of the catalytic triad are highlighted in pink. (A, B) Top‐down and front view of ubiquitin in USP7; (C, D) top‐down and front view of the predicted preferential pose for **7a** in USP7 with an AutoDock score of –7.68. The compound is oriented toward the catalytic aspartate and histidine; (E, F) top‐down and front view of the lower scoring pose for **7a** in USP7 with an AutoDock score of –6.08. Here, the compound is oriented toward the ubiquitin tail binding groove.

Compound **7a**, obtained as a hit in our previous study, was covalently docked into the crystal structure of USP7 in complex with ubiquitin (PDB Code 5KYD) [32] by using AutoDock to see how the compound might orient in the catalytic domain of USP7. The results of the covalent docking studies showed that **7a** could either point to the direction of the ubiquitin tunnel or toward the catalytic histidine and aspartate belonging to the catalytic triad (Figure 1). This unusual pose of **7a** in the catalytic domain of USP7 showed better AutoDock scores (–7.68) than the pose in the direction of the ubiquitin (–6.08). This preferentially predicted binding mode of **7a** would be novel as a previously published structure of a covalent USP7 inhibitor shows an opposite orientation toward the ubiquitin‐tail binding groove [10]. Moreover, the unconventional pose obtained from docking showed space near the catalytic triad around compound **7a**'s pyrazole residue. Therefore, we decided to either extend the pyrazole *N*‐substituent or to replace the pyrazole with bigger moieties. Importantly, this part of the molecule was synthetically quite accessible, further supporting our decision to prioritize the variation of **7a**'s pyrazole moiety for SAR studies.

### 2.2 Chemistry

**Scheme 1:**
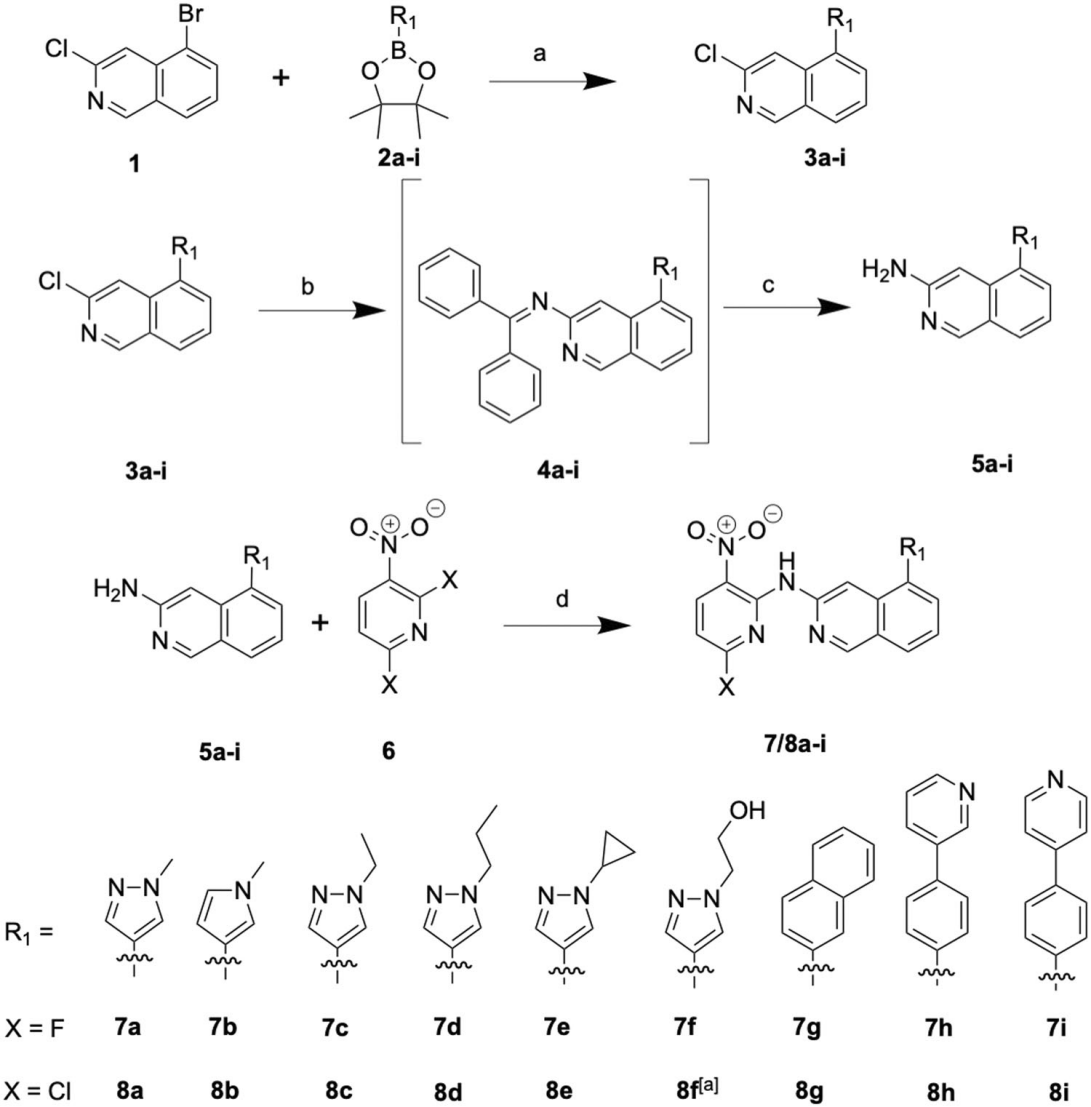
Synthesis procedure. (a) Pd(dppf)Cl_2_, Na_2_CO_3_, 1,4‐dioxane/water mixture (3:1), 105°C, 18 h; (b) benzophenone imine, Pd(OAc)_2_, BINAP, *t*‐BuONa, toluene, 120°C, 18 h; (c) 2 N HCl_aq_., 75°C, 1 h; (d) DIPEA, 1,4‐dioxane, reflux, 101°C, 20 h. ^[a]^N/A.

All compounds were synthesized in four steps starting from commercially available 3‐chloro‐5‐bromoisoquinoline (**1**) and were either based on or were synthesized in analogy to the synthesis protocol of Wydra et al. [33] To this end, starting material **1** was reacted in a Suzuki coupling with one of 8 different boronic acid esters (**2a–i**) based on a modified synthesis protocol of Innocenti et al. [34] The boronic acid ester was coupled regioselectively at position 5 of the isoquinoline due to the preferential reaction at the brominated site. The Suzuki coupling products **3a–i** were obtained with yields ranging from 37% to 93%. In the following reaction, an amino group in the 3‐position of the isoquinoline was introduced by following a modified protocol by Wolfe et al., where benzophenone imine was employed as an ammonia surrogate in a Buchwald‐type coupling reaction [35]. The advantage of this procedure was that it avoids double arylation while the formed imine intermediates (**4a–i**) are easily hydrolyzable by the addition of an aqueous acid (HCl) circumventing the isolation of the intermediate products **4**. Following this 2‐step procedure, primary arylamines **5a–i** were obtained at yields between 5% and 90%. These arylamines **5a–i** were reacted with commercially available 2,6‐difluoro‐3‐nitropyridine or 2,6‐dichloro‐3‐nitropyridine (**6**) in the presence of *N,N*‐diisopropylethylamine (DIPEA) to yield the final compounds (**7/8a–i** without **8f**) through regioselective nucleophilic aromatic substitution (S_N_Ar) (Scheme 1). The correct regiochemistry was previously confirmed by a small molecule X‐ray crystal structure of compound **8a** [33].

### 2.3 Biophysical Evaluation

#### 2.3.1 DSF

The influence of the synthesized compounds on the melting temperature (*T*_m_) of USP7 and its mutant USP7asoc (active site only cysteine) was investigated by DSF. USP7asoc only retains the catalytic Cys223 while all the other six cysteines are mutated to serine [30]. DSF was selected for initial assessment since it is an efficient and rapid primary screening method readily available to our lab. This method relies on the detection of a shift in melting temperature (∆*T*_m_) and hence in protein stability upon compound binding [36, 37, 38].

**Figure 2:**
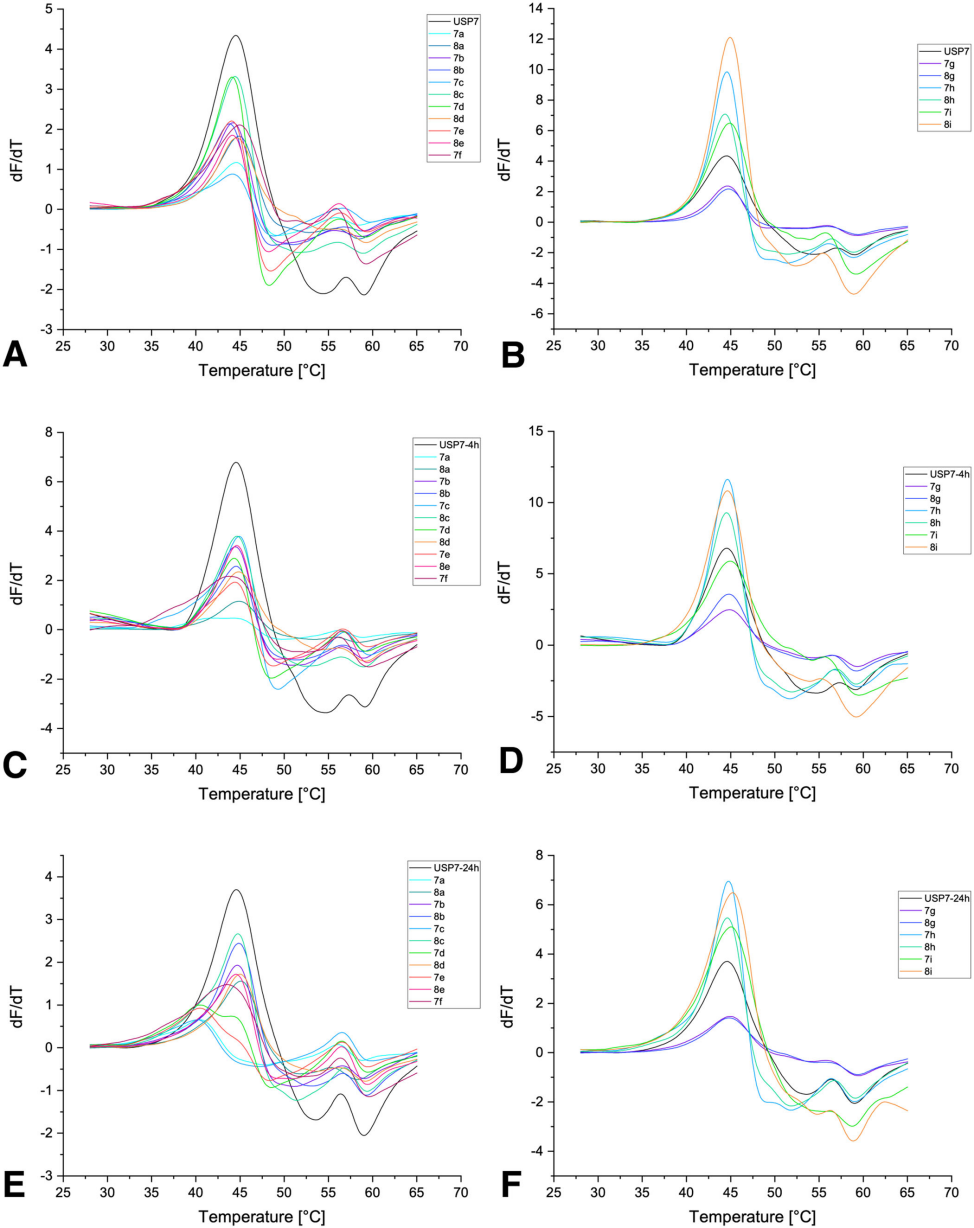
Overview of the first derivatives of the melting curves of USP7 after 30 min (A, B), 4 h (C, D), and 24 h (E, F) of incubation at 20°C with the synthesized compounds.

**Figure 3:**
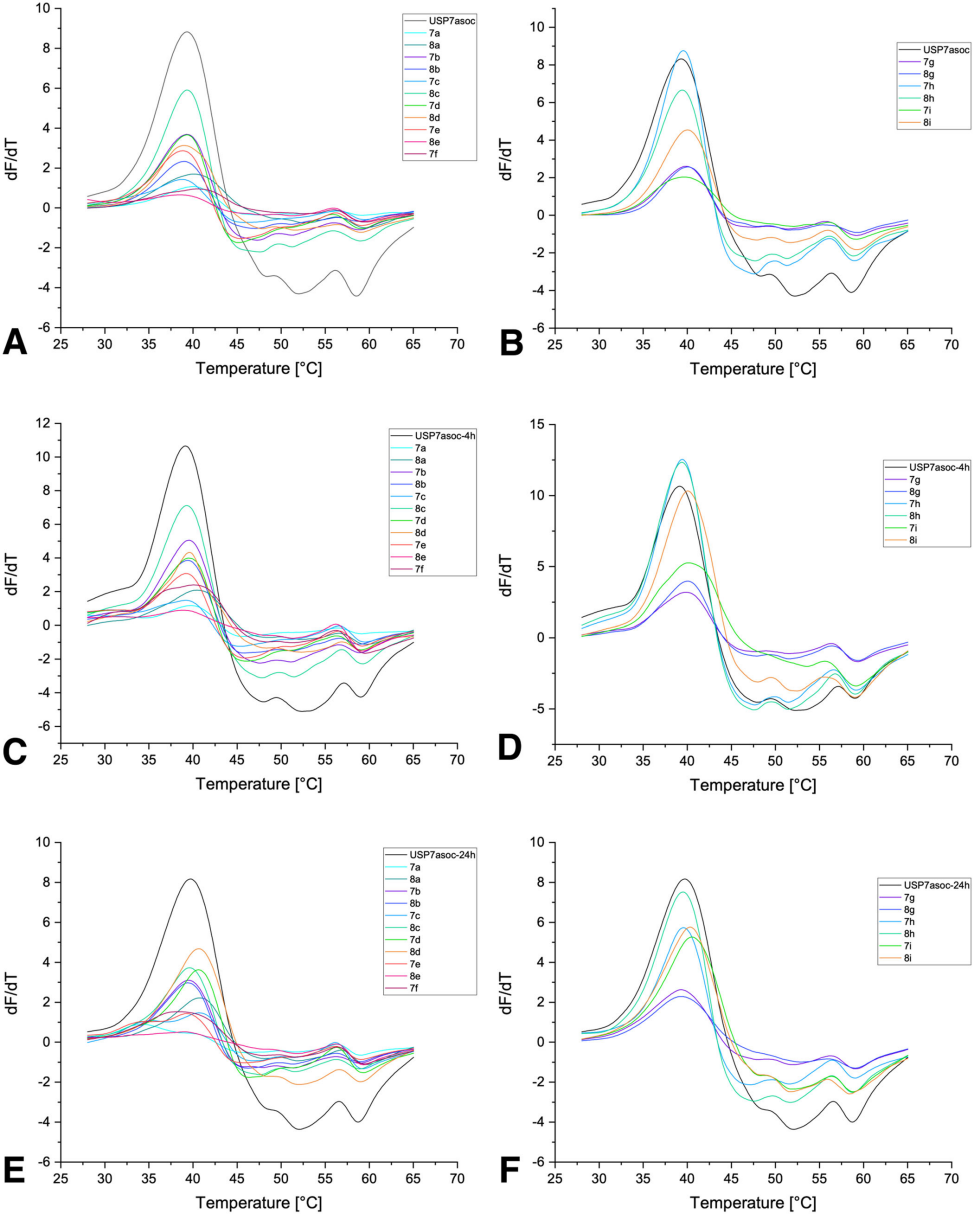
Overview of the first derivatives of the melting curves of USP7asoc after 30 min (A, B), 4 h (C, D), and 24 h (E, F) of incubation at 20°C with the synthesized compounds.

An important consideration when assessing covalent inhibitors is the time dependence of covalent target occupancy and thus potency, which is determined by the reaction kinetics of the covalent binding event [39]. To allow for drawing conclusions on compounds with slower reaction kinetics, incubation times of 30 min, 4 h, and 24 h at 20°C were chosen for the DSF screen with USP7 (Table 1 and Figures 2 and 3). The threshold at which compounds were considered (de‐)stabilizers was defined at 0.5°C. In parallel, the intrinsic reactivity of a set of representative compounds was evaluated in a glutathione reactivity assay. Here, compounds with a fluoride leaving group (*t*_1/2_ between 18 and 62 h) were generally more reactive than the respective chloro analogs (*t*_1/2_ typically > 100 h). This result aligns well with the commonly observed reactivity trends in S_N_Ar reactions, where the entry of the nucleophile (and not the loss of the leaving group) is the rate‐determining step [28]. Notably, we observed a few exceptions from this trend, which were probably a result of poor compound solubility and compound precipitation at early time points affecting our measurements. Data obtained from the GSH reactivity studies are shown in Supporting Information S2: Table S2.1 and Figures S2.1–2.3.

**Table 1:** Overview of ∆*T*_m_ ± SD of USP7 and USP7asoc (8 µM protein) incubated with 250 µM of the synthesized compounds (protein‐to‐compound ratio 1:31.25) for 30 min, 4 h, and 24 h at 20°C.

	USP7	USP7	USP7	USP7asoc	USP7asoc	USP7asoc
	30 min	4 h	24 h	30 min	4 h	24 h
compounds	∆*T*_m_ ± SD (°C)	∆*T*_m_ ± SD (°C)	∆*T*_m_ ± SD (°C)	∆*T*_m_ ± SD (°C)	∆*T*_m_ ± SD (°C)	∆*T*_m_ ± SD (°C)
**7a** (F)	–0.13 ± 0.08	–0.23 ± 0.06	–4.20 ± 0.10	–0.10 ± 0.09	–0.25 ± 0.09	–6.18 ± 0.04
**8a** (Cl)	0.20 ± 0.09	0.45 ± 0	0.20 ± 0.09	0.10 ± 0.27	0.10 ± 0.09	0.10 ± 0.09
**7b** (F)	–0.10 ± 0.09	0.00 ± 0	0.00 ± 0	0.05 ± 0.09	0.45 ± 0.12	0.38 ± 0.06
**8b** (Cl)	–0.65 ± 0.09	–0.10 ± 0.09	0.15 ± 0.09	–0.30 ± 0	0.35 ± 0.09	0.45 ± 0
**7c** (F)	–0.45 ± 0.12	–0.45 ± 0	–4.55 ± 0.09	–0.25 ± 0.23	0.20 ± 0.09	0.23 ± 0.08^b^
**8c** (Cl)	–0.15 ± 0.12	0.15 ± 0	0.15 ± 0	0.05 ± 0.09	0.25 ± 0.12	0.33 ± 0.08
**7d** (F)	–0.30 ± 0.06	–0.18 ± 0.03	–4.10 ± 0.09	0.00 ± 0	0.03 ± 0.08	0.35 ± 0.06
**8d** (Cl)	0.25 ± 0.09	0.30 ± 0	0.25 ± 0.09	–0.20 ± 0.09	0.45 ± 0.12	0.42 ± 0.16
**7e** (F)	–0.23 ± 0.06	0.02 ± 0.03	–4.20 ± 0	–0.35 ± 0.09	–0.13 ± 0.04	0.40 ± 0.09^b^
**8e** (Cl)	–0.40 ± 0.09	–0.15 ± 0	–0.15 ± 0	–0.70 ± 0.18	N/A^a^	N/A^a^
**7f** (F)	0.00 ± 0.12	–1.15 ± 0.04	–1.95 ± 0.19	0.48 ± 0.09	–0.23 ± 0.08	–1.58 ± 0.09
**7g** (F)	0.10 ± 0.12	0.30 ± 0	0.25 ± 0.09	0.63 ± 0.07	0.25 ± 0.10	0.38 ± 0.06
**8g** (Cl)	0.25 ± 0.12	0.30 ± 0	0.25 ± 0.09	0.63 ± 0.07	0.38 ± 0.08	0.40 ± 0.17
**7h** (F)	0.00 ± 0.12	0.15 ± 0	0.20 ± 0.09	0.30 ± 0	0.35 ± 0.09	0.35 ± 0.04
**8h** (Cl)	–0.25 ± 0.09	0.00 ± 0	0.05 ± 0.09	0.15 ± 0	0.35 ± 0.09	0.28 ± 0.04
**7i** (F)	–0.13 ± 0.09	–0.40 ± 0.08	–0.45 ± 0.09	–0.30 ± 0.12	–0.15 ± 0	0.05 ± 0.17
**8i** (Cl)	0.05 ± 0.09	–0.78 ± 0.08	–0.03 ± 0.11	0.10 ± 0.17	–0.15 ± 0.15	–0.15 ± 0.12

*Note:* Measurements were performed at least in triplicate. Hits with a Δ*T*_m_ of > 0.5°C are highlighted in red.

^a^ No evaluable melting curves were obtained.

^b^ Melting curve with a small shoulder corresponding to a ∆*T*_m_ of about –5°C

The pyrazole **7a**, which we identified previously to covalently modify USP7 [30], decreased the *T*_m_ of USP7 and USP7asoc exclusively after 24 h of incubation by about –4.20°C for USP7 and –6.18°C for USP7asoc (Figures 2E and 3E). Despite also binding USP7 covalently (vide infra), its chloro analog did not influence the *T*_m_ of both proteins across incubation times [30]. However, the amount of detected covalent bonding in the intact protein MS experiment was substantially lower than for **7a**, which could explain the difference in behavior in the DSF experiment (Section 2.3.2). Neither of the corresponding pyrrole derivates **7b** and **8b** influenced the *T*_m_ of USP7 across any incubation time except for the chloro derivative **8b** showing a small decrease after 30 min of incubation (Δ*T*_m_ –0.65°C) (Figure 2A). This difference can be attributed to the lack of the second nitrogen in the five‐membered ring potentially changing the binding mode or noncovalent binding affinity to USP7.

The following seven compounds only differed in the pyrazole ring's *N*‐substituent. Here, compounds **7c** and **8c** carry an ethyl group, compounds **7d** and **8d** carry a propyl group, compounds **7e** and **8e** carry a cyclopropyl group, and compound **7f** carries a 2‐hydroxyethyl group. All chloro analogs (**8c**, **8d**, and **8e**) did not show any effects on the melting temperatures of either USP7 or USP7asoc (Figures 2 and 3). However, while having only very weak effects on USP7, **8e** showed a decrease in the *T*_m_ of USP7asoc after 30 min of incubation by –0.70°C (Figure 3A). This result was surprising since no other chloride leaving group‐containing compound actually destabilized native or mutant USP7 to this extent. Moreover, this compound also appeared to be more reactive compared with the other chloro derivatives (*t*_1/2_ = 11 h in the GSH assay). However, it cannot be excluded that this behavior is linked to a low compound solubility. Unfortunately, the melting curves of **8e** after 4 h and 24 h of incubation with USP7asoc could not be evaluated, likely due to fluorescence quenching and low signal intensity [36, 40]. The fluoro analogs (**7c**, **7d**, and **7e**) destabilized USP7 only after 24 h of incubation (Figure 2E). The extent of destabilization was in the same range as for **7a** (i.e., Δ*T*_m_ around –4°C). However, **7d** showed a smaller second peak at a higher temperature, possibly corresponding to unmodified USP7. Interestingly, in contrast to **7a**, the aforementioned fluoro analogs (**7c**, **7d**, and **7e**) did not influence USP7asoc across any incubation time. Notably, a small shoulder was recognizable at lower melting temperatures for compounds **7c** and **7e** after 24 h of incubation with USP7asoc. The difference between the two peaks was about –5°C for both compounds and could represent a covalently modified USP7asoc variant (Figure 3E). The last fluoro derivative in this series was **7f**, bearing a hydroxyethyl moiety at the pyrazole. It already showed some destabilization of USP7 after 4 h of incubation, namely by about –1°C (Figure 2C). After 24 h of incubation, compound **7f** destabilized both native USP7 and USP7asoc by −1.95°C (Figure 2E) and by –1.58°C, respectively (Figure 3E). Despite already destabilizing native USP7 after 4 h, the extent of destabilization was smaller compared with **7a**.

The two naphthyl derivatives **7g** and **8g** exclusively stabilized USP7asoc but only after 30 min of incubation and to a small degree, namely by 0.63°C (Figure 3B). No other influences on the melting temperature of either USP7 or USP7asoc were visible.

The *T*_m_ shifts of the phenylpyridines **7h**, **8h**, **7i**, and **8i** on either USP7 or USP7asoc were weak across any incubation times and mostly below the 0.5°C threshold (Figures 2 and 3). Only the phenylpyridine analog **8i** (pyridine *N* at position 4 with chloride as leaving group) decreased the *T*_m_ of USP7 after 4 h of incubation by –0.78°C (Figure 3D). Since MS data showed no or only weak covalent modification in all cases (vide infra), we hypothesize that the larger naphthyl or biaryl moieties do not properly fit into the binding pocket, thereby lowering affinity and/or hampering the formation of a well‐aligned pre‐reactive complex.

In summary, the extent of destabilization observed for the *N*‐alkyl pyrazole analogs **7c**, **7d**, and **7e** was in the same range as for **7a**. However, and in contrast to **7a**, none of these compounds showed a significant influence on the *T*_m_ of USP7asoc after 24 h of incubation. Hydroxyethyl‐substituted analog **7f** was the only one among the new compounds showing a decrease in the *T*_m_ of both USP7 and USP7asoc after this incubation time, albeit to a lower extent as the parent molecule **7a**. Most other compounds (**8c**, **8d**, **8e**, **7g**, **8g**, **7h**, **8h**, **7i**, and **8i**) did not influence the melting temperature of any of the two proteins to a notable extent. Similarly, pyrrole analogs **7b** and **8b** did not notably influence the *T*_m_ of either USP7 or USP7asoc, which leads to the conclusion that the second nitrogen in the five‐membered ring has a contribution to binding to USP7.

#### 2.3.2 Intact Protein MS

The DSF results described above were validated by UHPLC‐ESI‐MS to verify possible covalent binding through a corresponding mass shift. Intact protein MS is an important screening method in irreversible‐covalent hit identification and is especially suitable for covalent fragment‐based drug discovery (FBDD). It allows for the identification of covalent binders by detecting covalent protein‐compound adducts, thus making use of the stability of the covalent bond [41, 42, 43].

The covalent modifications of USP7 and USP7asoc were confirmed by the deconvoluted protein MS spectra for 10 out of the 15 SAR compounds derived from **7a** and **8a**. The deconvoluted MS spectra of selected compounds indicated mostly single but also some additional modifications. All compounds reacted in a nucleophilic aromatic substitution reaction (S_N_Ar) with either fluoride or chloride as the leaving group.

**Figure 4:**
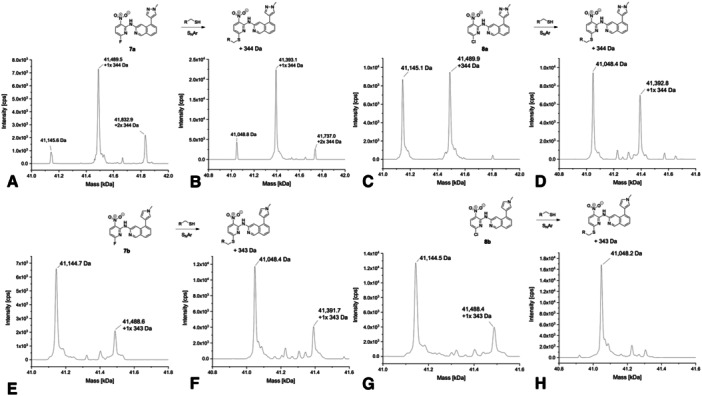
Deconvoluted mass spectra of **7a** (A with USP7 and B with USP7asoc), **8a** (C with USP7 and D with USP7asoc), **7b** (E with USP7 and F with USP7asoc), and **8b** (G with USP7 and H with USP7asoc) after 24 h of incubation at 20°C (protein‐to‐compound ratio 1:31.25, theoretical mass of unmodified USP7: 41,145.61 Da; USP7asoc: 41,049.22 Da).

The deconvoluted mass spectra of **7a** and **8a** have been reported previously [30]. However, for a better overview, they are shown and described here once again (Figure 4A–D). The spectrum of **7a** showed a mono‐arylated protein species as the main peak, with unmodified and double‐arylated USP7 as additional smaller peaks. The spectrum of USP7asoc incubated with **7a** looked nearly identical with a single covalent modification as the main peak, with two additional smaller peaks (unmodified and twofold arylated USP7asoc) also detectable. In the spectrum of the chloro analog **8a** with USP7, the unmodified and mono‐arylated protein species were visible with nearly the same intensity (Figure 4C). However, the spectrum with USP7asoc showed unmodified protein species as the main peak with an additional signal corresponding to onefold arylated protein species. This result is in line with the GSH assay, where **7a** (*t*_1/2_ = 35 h) appeared to be more reactive than **8a** (*t*_1/2_ > 100 h), which reflects the common reactivity order of S_N_Ar reactions (Supporting Information S2: Table S2.1 and Figure S2.1) [28, 29].

Corresponding MS spectra of the pyrrole analog **7b** showed unmodified protein species as a main peak for both USP7 and USP7asoc. An additional signal corresponding to mono‐arylated protein species was visible for both proteins as well (Figure 4E,F). Interestingly, the spectrum of the chloro analog **8b** only showed unmodified protein for USP7asoc. In the case of USP7, the unmodified protein species was dominant with an additional smaller signal of the mono‐arylated species (Figure 4G,H). This indicates that the chloro analog may have modified another cysteine in USP7. Another possible reason is that USP7asoc has a slightly different structure compared to USP7 due to the mutations, which could result in the compound being unable to adopt an orientation that enables an efficient reaction with the catalytic cysteine in USP7asoc. This result of the intact protein MS again reflects the differences in reactivity between fluoride and chloride acting as a leaving group in S_N_Ar reactions and was also confirmed by the reactivity measurements with GSH (Supporting Information S2: Table S2.1 and Figure S2.1). Here, compound **7b** proved to be approximately as reactive as **7a**, while compound **8b** showed equivalent stability as its analog **8a**.

**Figure 5:**
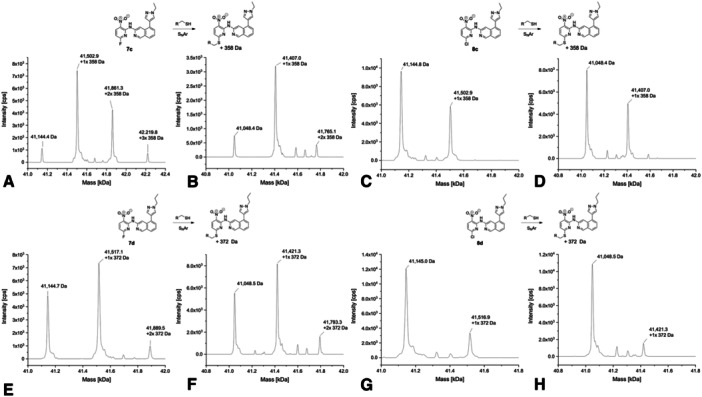
Deconvoluted mass spectra of **7c** (A with USP7 and B with USP7asoc), **8c** (C with USP7 and D with USP7asoc), **7d** (E with USP7 and F with USP7asoc), and **8d** (G with USP7 and H with USP7asoc) after 24 h of incubation at 20°C (protein‐to‐compound ratio 1:31.25, theoretical mass of unmodified USP7: 41,145.61 Da; USP7asoc: 41,049.22 Da).

The intact protein mass spectra of *N*‐ethyl pyrazole analog **7c** mainly showed the attachment of a single molecule to USP7 and USP7asoc (Figure 5A,B). Both proteins showed additional signals corresponding to unmodified and double‐arylated USP7 and USP7asoc protein species, respectively. This indicates that compound **7c** also binds to an amino acid other than cysteine in USP7asoc. However, the presence of the double‐arylated protein species should be interpreted with caution, as it was detected at a very low signal intensity. For the less reactive chloride analog **8c**, only unmodified USP7 and USP7asoc were detected with a smaller peak corresponding to the single‐arylated protein species (Figure 5C,D). In line with this observation, compound **8c** did not show a thermal shift in the DSF experiment.

Upon incubation with propyl‐substituted compound **7d**, which differs from **7c** only by the elongation of the pyrazole *N*‐alkyl by one methylene unit, the spectra showed a single modified protein as the main signal as well as two more peaks representing unmodified and double‐arylated USP7 and USP7asoc species, respectively, with the latter appearing clearly smaller (Figure 5E,F). In the spectra of the less reactive chloro analog **8d**, unmodified USP7 and USP7asoc were again dominant with an additional minor peak representing the mono‐arylated protein species (Figure 5G,H).

**Figure 6:**
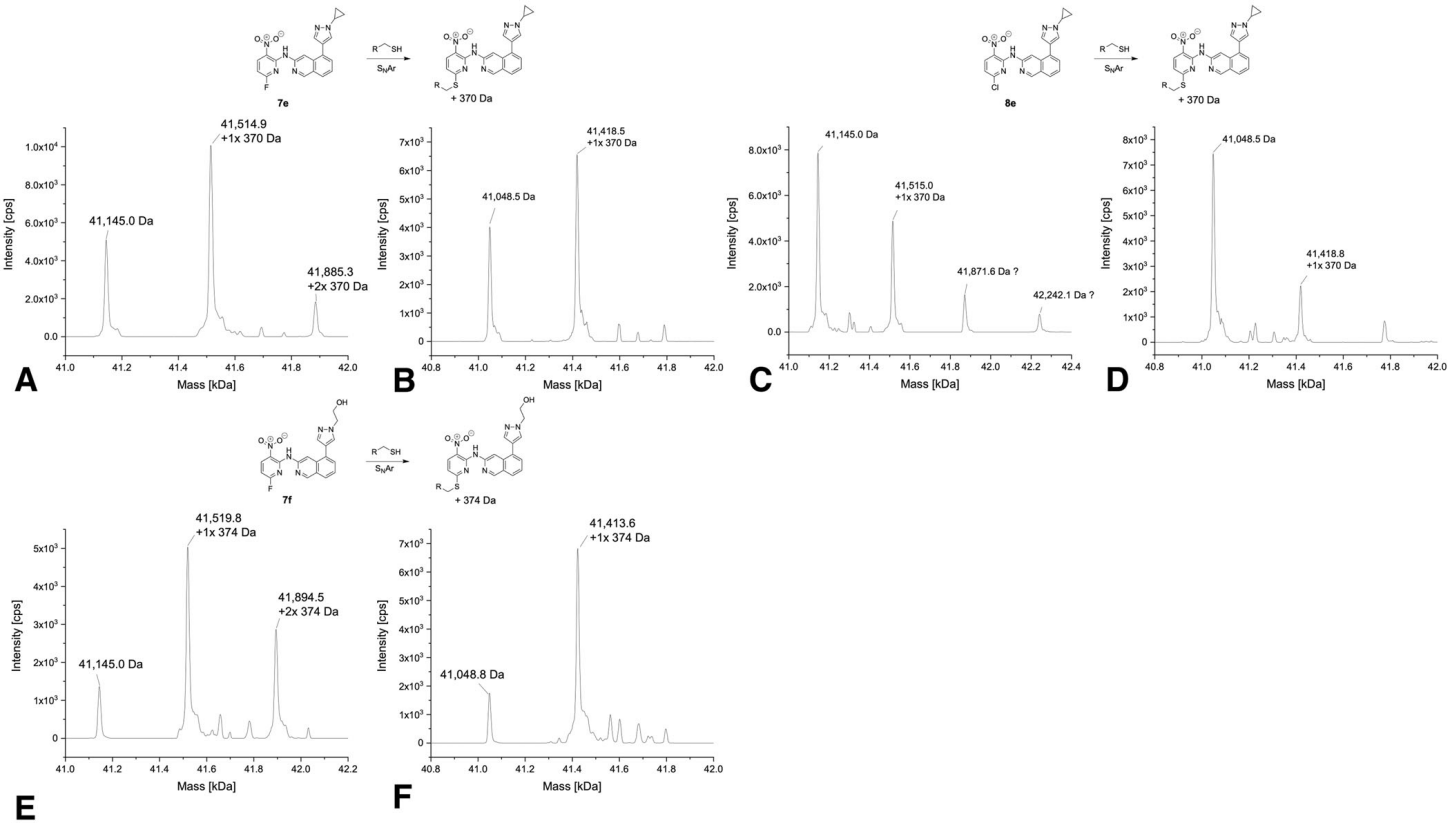
Deconvoluted mass spectra of **7e** (A with USP7 and B with USP7asoc), **8e** (C with USP7 and D with USP7asoc), and **7f** (E with USP7 and F with USP7asoc) after 24 h of incubation at 20°C (protein‐to‐compound ratio 1:31.25, theoretical mass of unmodified USP7: 41,145.61 Da; USP7asoc: 41,049.22 Da).

Covalent modification was also detected for the cyclopropyl analog **7e**, where the spectra showed mono‐arylated USP7 and USP7asoc species as main peaks (Figure 6A,B). Signals corresponding to unmodified USP7 and USP7asoc were visible as well. The spectrum of USP7 also displayed a double‐arylated protein species. In a similar fashion to all the other aforementioned chloro analogs, **8e** mainly showed signals for unmodified USP7 and USP7asoc along with additional smaller signals representing single‐arylated USP7 and USP7asoc species (Figure 6C,D). Notably, the spectrum of USP7 additionally showed signals corresponding to a protein mass that could not be assigned. A mass increase of 356 Da instead of 370 Da was observed and another 370 Da mass increase from the previous peak to the next signal. This behavior was unique compared with the other compounds, yet we cannot fully rationalize it. In addition, while the GSH half‐life of the fluoro analog **7e** could not be determined, **8e** seemed to be the most reactive compound in the GSH assay (*t*_1/2_ = 11 h). However, this result should be interpreted with caution as there were some inconsistencies in the data obtained, which may be linked to limited compound solubility. This may also explain why we could not determine a GSH half‐life for compound **7e** (Supporting Information S2: Table S2.1 and Figure S2.2).

The mass spectrum of the *N*‐hydroxyethyl substituted compound **7f** with a fluoride leaving group showed single‐arylated USP7 and USP7asoc as a main signal (Figure 6E,F) as well as additional signals for unmodified protein species. USP7 also displayed double‐arylated protein species as second highest signal. Consequently, it can be assumed that it was indeed the catalytic Cys223 that was addressed covalently because the mass spectrum of USP7asoc mainly showed single‐arylated protein species.

**Figure 7:**
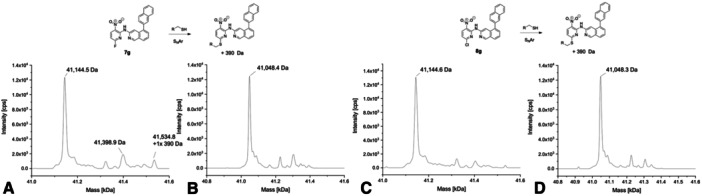
Deconvoluted mass spectra of **7g** (A with USP7 and B with USP7asoc) and **8g** (C with USP7 and D with USP7asoc) after 24 h of incubation at 20°C (protein‐to‐compound ratio 1:31.25, theoretical mass of unmodified USP7: 41,145.61 Da; USP7asoc: 41,049.22 Da).

The naphthyl analog **7g** mainly displayed the unmodified USP7 and USP7asoc species (Figure 7A,B). In the mass spectrum of USP7, additional signals were visible with one peak corresponding to a mono‐arylated protein species. However, this signal was not pronounced and thus should be interpreted with caution. For the chloro analog **8g**, only unmodified USP7 and USP7asoc were detected with no additional signals visible (Figure 7C,D). Notably, both compounds exclusively stabilized USP7asoc only after 30 min of incubation and to a small degree, namely by 0.63°C, and did not show any other thermal shift in the DSF experiment with USP7. Consequently, this observed stabilization might be considered an outlier. Compound **7g** showed nearly the same GSH reactivity as compound **7a**, suggesting that the bulky naphthyl substituent impedes the formation of the pre‐reaction complex (Supporting Information S2: Table S2.1 and Figure S2.3).

**Figure 8:**
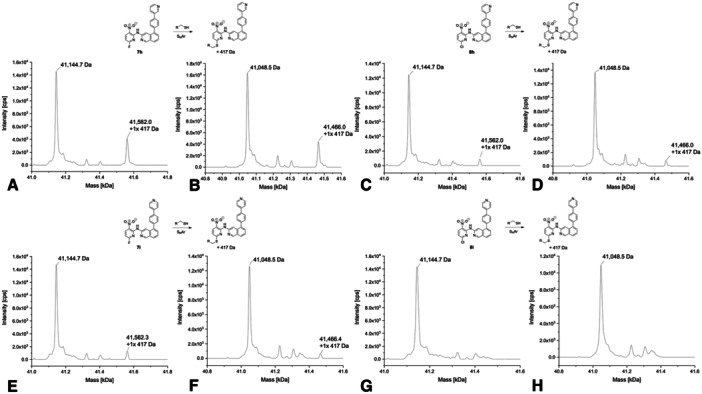
Deconvoluted mass spectra of **7h** (A with USP7 and B with USP7asoc), **8h** (C with USP7 and D with USP7asoc), **7i** (E with USP7 and F with USP7asoc), and **8i** (G with USP7 and H with USP7asoc) after 24 h of incubation at 20°C (protein‐to‐compound ratio 1:31.25, theoretical mass of unmodified USP7: 41,145.61 Da; USP7asoc: 41,049.22 Da).

In the spectra of USP7 and USP7asoc incubated with the phenylpyridine‐substituted derivative **7h**, the unmodified protein species was again dominant with one smaller signal corresponding to the mono‐arylated protein (Figure 8A,B). Expectedly, similar results were obtained for the less reactive chloro analog **8h**. However, here intensities for the smaller signal (corresponding to single‐arylated protein species) were close to various impurities or minor protein peaks and should therefore be interpreted with care (Figure 8C,D).

Compounds **7i** and **8i** also feature a phenylpyridine moiety instead of the pyrazole but with the pyridyl residue linked via the 4‐position compared to position 3 in compounds **7h** and **8h**. All four compounds depicted unmodified USP7 and USP7asoc as the main peak (Figure 8A–H). Interestingly, also here, only the fluoro analog, **7i**, showed a weak additional signal corresponding to mono‐arylated USP7 and USP7asoc, but similar considerations as for **7h** apply in terms of data interpretation (Figure 8A–D).

In summary, only the more reactive fluoro analogs bearing a pyrazole *N*‐(cyclo)alkyl substituent showed mono‐arylated forms of USP7 and USP7asoc as main peak in many of the mass spectra supporting the catalytic Cys223 as the primary covalent labeling site. Covalent modification was significantly reduced for the less reactive chloro analogs. Importantly, bigger moieties replacing the pyrazole (i.e., the phenylpyridine or naphthyl analogs **7/8g–i**) or the replacement by pyrrole impeded the formation of a covalent adduct. To confirm the functional relevance of the above results, interesting compounds were further evaluated in an Ub‐AMC enzyme activity assay (vide infra) to assess the inhibitory effect on USP7.

#### 2.3.3 IC_50_ Determination

**Figure 9:**
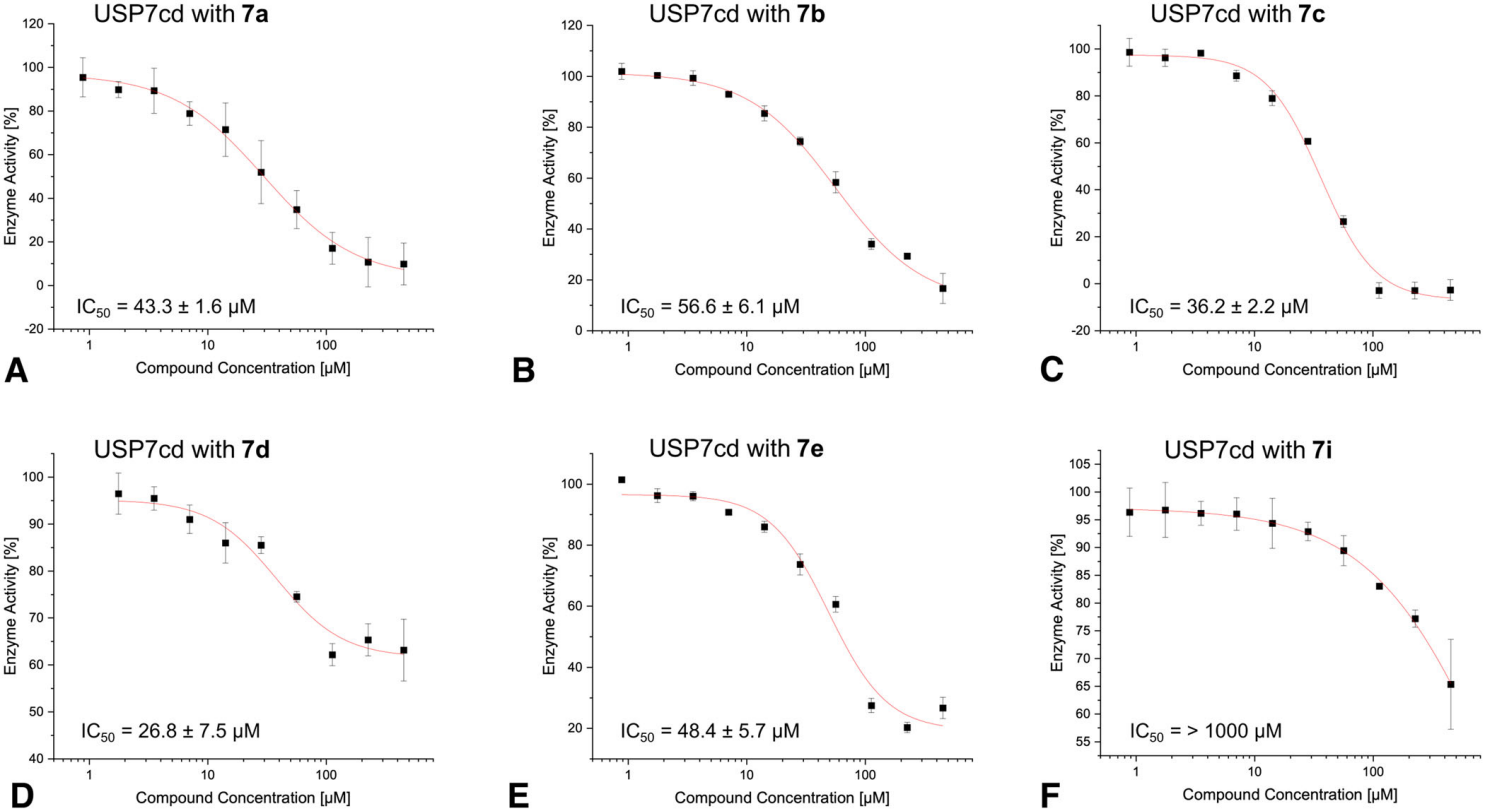
Compound (A **7a**; B **7b**; C **7c**; D **7d**; E **7e**; F **7i**) inhibition with 500 nM Ub‐AMC and USP7cd (10 nM) and the corresponding IC_50_ values after 24 h of incubation time.

Despite depending on incubation time [44, 45], half‐maximal inhibitory concentrations (IC_50_ values) obtained under standardized conditions have been shown to present a suitable measure to rank the potency of covalent inhibitors, especially within a structural series [46]. Therefore, USP7 was incubated for 24 h with ten different compound concentrations, and IC_50_ values were then obtained with a fluorescent DUB substrate (Ub‐AMC) (Figure 9A–F). The pre‐incubation times were chosen to account for the slow covalent binding kinetics observed in the previous experiments.

Fluoro derivative **7a** inhibited USP7 (cd; catalytic domain) at an IC_50_ value of 43 µM (Figure 9A). The structurally similar analog **7b** with a pyrrole ring instead of the pyrazole ring (IC_50_ = 57 µM) bound to USP7 with a lower affinity compared with the other compounds (Figure 9B), which is in line with the intact protein MS results discussed above. The closely related pyrazole *N*‐ethyl analog **7c** showed a slightly higher potency (IC_50_ = 36 µM) than compound **7a** (Figure 9C). *N*‐propyl analog **7d** displayed the highest potency with an IC_50_ value of 27 µM, thus being two times more potent than compound **7a** (Figure 9D). Nevertheless, the highest compound **7d** concentration (450 µM) was not enough to achieve an enzyme activity close to 0%. The flattening of the dose–response curves at enzyme activity levels significantly above zero was a feature observed for several compounds and may be related to limited compound solubility at high compound concentrations. The IC_50_ value of *N*‐cyclopropyl derivative **7e** (IC_50_ = 48 µM) was similar to compound **7a** (Figure 9E). Unfortunately, it was not possible to determine the IC_50_ values of the chloro analogs **8d** and **8e** due to solubility problems. Moreover, the fluoro analog **7f** with an *N*‐hydroxyethyl moiety at the pyrazole ring showed a fluorescence at the wavelength of detection, hampering the collection of conclusive data.

The chloro analogs of the identified hit compounds were not measured because they did not show strong modification of USP7asoc in the MS experiment. Similarly, IC_50_ values were not determined for the compounds with bulky substituents (**7g**, **8g**, **7h**, **8h**, and **8i**) since none of them showed a pronounced covalent modification of USP7 in the intact protein MS experiments. As an exception, the IC_50_ value was assessed for compound **7i** to gain more insights on how one of the larger moieties affects noncovalent binding to USP7. The IC_50_ value calculated for **7i** was over 1000 µM (Figure 9F), supporting our hypothesis that steric bulk at the isoquinoline 5‐position impairs the formation of the pre‐reaction complex.

In summary, the slight increase in the hydrophobicity and size of the side chain through elongation of the length of the pyrazole *N‐*alkyl substituent results in lower IC_50_ values, indicating higher affinity to USP7, while the effect of a cyclopropyl group replacing the methyl group in the parent **7a** was rather neutral. Removal of one of the pyrazole nitrogen atoms resulted in a slight loss in affinity. Nevertheless, the changes in inhibitory activity remain relatively small.

#### 2.3.4 Evaluation of the Obtained Results

**Figure 10:**
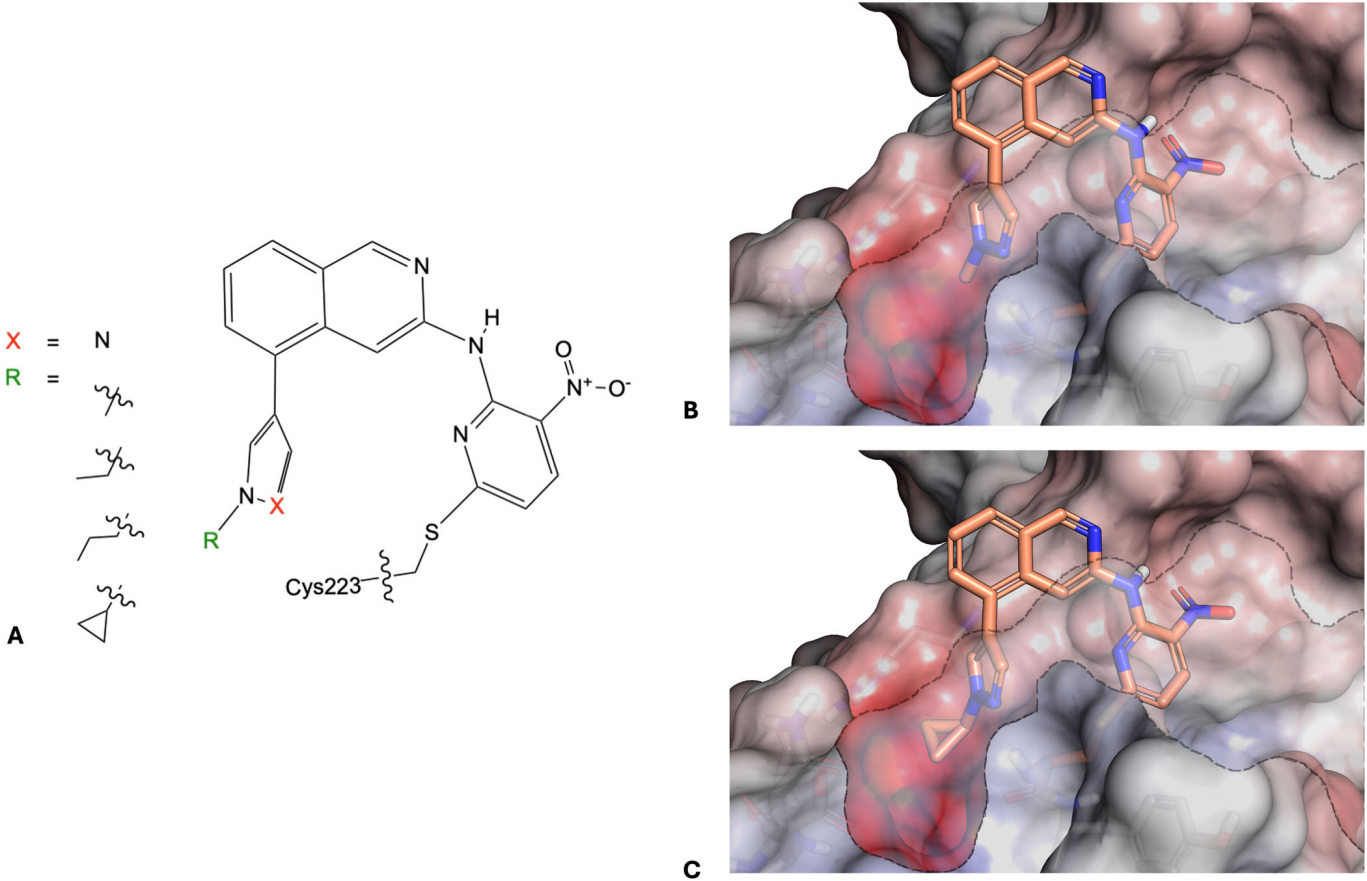
Overview of the obtained results: (A) Structures of the best SAR compounds and docking results of **7a** (B) and **7e** (C), respectively, in USP7 (PDB Code: 5KYD). Shown is the solvent‐excluded surface with a cutting plane revealing the addressable pocket toward the catalytic aspartate and histidine.

Figure 10 gives a summary of the results shown. First, all chloro analogs (**8a–i**) showed only a very slight influence on the melting temperature of USP7 and USP7asoc. Additionally, none of them clearly displayed modified protein species in the deconvoluted mass spectra. This is in line with the reactivity measurements using GSH, indicating a substantially reduced intrinsic reactivity arising from the fluoro‐to‐chloro replacement for most compounds.

The results also showed that the second nitrogen in the pyrazole ring had an effect since the pyrrole derivatives **7b** and **8b** showed a different behavior than prototype **7a** with respect to thermal shift of USP7 (no influence on the melting temperature in the DSF across any incubation time) and covalent bond formation (main MS peak now unmodified protein species). Interestingly, this did not cause a significant loss of affinity compared with the pyrazole analog **7a** in the activity assay (IC_50_ 43 µM for **7a** vs. 57 µM for **7b**). Furthermore, all newly synthesized *N*‐alkylpyrazole derivatives (**7c**, **7d**, **7e**, and **7f**) depicted similar spectra in the intact protein MS with single arylation representing the main peak for USP7 and USP7asoc. However, they differed somewhat in the DSF experiment with USP7asoc. They all showed a decrease in the melting temperature after 24 h of incubation with USP7 (∆*T*_m_ around –4°C), but only **7a** (∆*T*_m_ around –6°C) and **7f** (∆*T*_m_ around –2°C) maintained a decrease in melting temperature with USP7asoc, which was only apparent after 24 h of incubation and less pronounced for **7f**. However, it should be noted that compounds **7c** and **7e** displayed a second smaller peak at lower temperatures (Δ*T* approximately –5°C) in the melting curve of USP7asoc, which may arise from a partial covalent modification and the simultaneous presence of two protein populations in the experiment. Furthermore, the IC_50_ determination suggests that a small elongation of the alkyl moiety at the pyrazole nitrogen leads to a weak gain in affinity to USP7. However, as soon as the substituent becomes sterically more demanding and/or less flexible (e.g., cyclopropyl **7e**), the affinity decreases again. Yet, the differences are not very pronounced, indicating that small alkyl residues were generally well tolerated. In case of larger aryl residues, as for example in **7i**, a drastic loss of affinity and only little conversion in the intact protein MS was observed. Consequently, it can be hypothesized that the space within the binding cleft is rather limited, and the compound may no longer be able to position itself into a productive pre‐reactive orientation. Additionally, the size of the halogen and its interaction in the noncovalent complex may also influence the orientation and electrophilicity of the C–X carbon atom and thus the kinetics of the reaction. This assumption was supported by the docking poses of compounds **7a** and **7e**, indicating a spatially restricted pocket next to the pyrazole nitrogen atom (Figure 10B,C). For further validation, we determined the X‐ray crystal structure of the **7a**/USP7 complex.

#### 2.3.5 Determination of the Binding Mode of Compound 7a by X‐Ray Crystallography

To confirm the predicted binding modes involving the catalytic Cys223 in USP7, prototype compound **7a** was co‐crystalized with the catalytic domain of USP7 (PDB: 9QJE with a 2.26 Å resolution). This compound was chosen since it had the most pronounced effects in the DSF experiment decreasing the *T*_m_ of USP7 and USP7asoc by about −4°C and −6°C, respectively, and showed covalent modification in the intact protein MS experiment.

**Figure 11:**
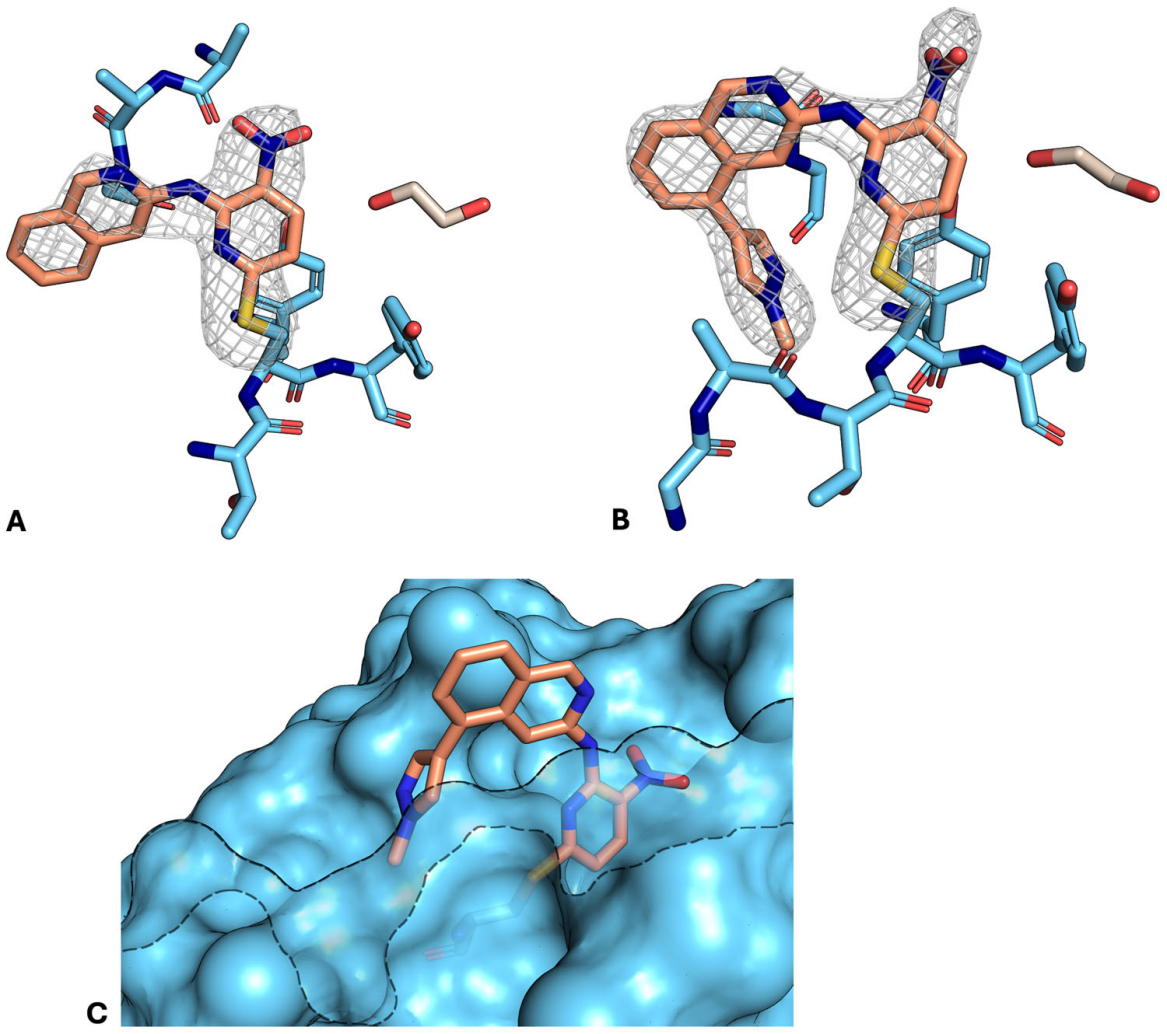
Overview of USP7 crystals (PDB: 9QJE) with a 2.26 Å resolution. (A) Binding mode of **7a** at chain A. The unbiased omit map is contoured at 2.5 sigma. (B) Binding mode of **7a** at chain B. The unbiased omit map is contoured at 2.5 sigma. (C) Pose of **7a** in USP7cd.

The crystal structure unambiguously confirmed the arylation of the catalytic Cys223 in the inactive state of USP7, as it is shown in Figure 11A–C. The compound could be modeled into the measured electron density with > 80% occupancy. The electron density of the new bond at Cys223 is clearly visible, whereas no additional density at any other cysteines or amino acids could be observed. As expected, the compound reacted in an S_N_Ar reaction with the fluorine atom being displaced. As mentioned before, the structure confirmed that the catalytic cysteine is modified in the inactive state of USP7, where it is less nucleophilic due to the disassembly of the catalytic triad. Compound **7a** generally shows an unconventional binding mode compared with all crystal structures, currently published in the PDB. It reaches deep into the region where the catalytic triad amino acids His464 and Asp481 are located (Figure 11C). Other published compounds are all oriented in the other direction of the ubiquitin binding channel [10, 17, 18, 20, 22, 47, 48, 49, 50, 51]. However, the exact binding mode must be interpreted with caution as compound **7a** is close to crystal contacts (Supporting Information S2: Figure S3.1). The omit map of compound **7a** is shown in Figure 11A,B. The binding mode determined experimentally is closer to the ubiquitin site than the docking pose, with less extension into the pocket near the catalytic aspartate and histidine. The beta sheet tightens the pocket, positioning the quinoline scaffold closer to the adjacent alpha‐helix. These discrepancies can be explained by the conformational differences in the structure used for docking (PDB: 5KYD) and the experimentally determined structure (PDB: 9QJE). Additionally, the observed crystal contact can also play a role in the final orientation of the ligand and slightly reposition it compared to a setting with the protein in solution.

The successful crystallization of **7a** with USP7, which revealed a new binding mode, provides a strong basis for further optimization to generate USP7 inhibitors with improved properties and high inhibitory potency.

## 3 Conclusion

In this study, 15 compounds derived from the initial hit **7a** with either fluoride or chloride as leaving group in an S_N_Ar reaction were successfully synthesized. Assessment by DSF and intact protein MS confirmed the covalent binding mode and provided a basis for SAR analysis. A covalent binding to USP7 and USP7asoc could be confirmed for 10 synthesized compounds. In line with common reactivity trends in S_N_Ar reactions [28, 29] and our GSH reactivity assays, the more reactive analogs with a fluoride leaving group showed increased binding to the catalytic Cys223. A fluorescence‐based enzyme activity assay with the fluorogenic substrate Ub‐AMC was used to determine the inhibitory effect of the compounds against the catalytic domain of USP7. Here, two compounds with elongated alkyl chains (**7c** and **7d**) showed a higher inhibitory activity on USP7 than **7a** (43 µM) with IC_50_ values of 36 and 27 µM, respectively, although the differences were not very pronounced. In another work on covalent USP7 inhibitors, Turnbull et al. report a lower IC_50_ for vinylsulfone‐derived compound FT827 but used another detector substrate in the enzymatic assay [10]. IC_50_ values for other covalent USP7 inhibitors were not completely comparable because they were often determined using full‐length USP7 [11, 13, 14, 19] or in cells [1, 12]. The examination of the electron density of compound **7a** with the covalent bond to Cys223 in a crystal structure gave evidence of an unconventional covalent binding mode and confirmed the predicted pose of **7a**. Compared with the published structures in the PDB, compound **7a** is the only covalently acting compound to point in the direction of the catalytic triad rather than in the common ubiquitin tunnel. As a next step, the complex structures of some additional compounds could be solved to determine how the alkyl chains influence the compound's orientation in the binding pocket. In combination with kinetic and computational studies, this could help to disentangle contributions of noncovalent binding affinity, pre‐orientation, and intrinsic reactivity to further exploit this unconventional binding mode for the development of potent and selective covalent USP7 inhibitors.

## 4 Experimental

### 4.1 Chemistry

#### 4.1.1 General

All chemicals and reagents used in the synthesis were of commercial quality and were used without additional purification. Reactions were monitored using thin‐layer chromatography (TLC) carried out on Merck Kieselgel 60 F_254_ plates (Merck KGaA, Darmstadt, Germany), and spots were detected under UV light at 254 and 366 nm. The compounds were purified with a preparative puriFlash XS520Plus system (Interchim S.A., Montluçon, France) equipped with a normal phase Grace Davison Davisil LC60A 20‐45 μm column (W.R. Grace and Company, Columbia, MD, USA). Merck Geduran Si 60–200 μm silica (Merck KGaA, Darmstadt, Germany) was used as a pre‐column.

The ^1^H and ^13^C NMR spectra were acquired with a Bruker Avance III HDX 400 or for compound **7f** (^13^C NMR) with a Bruker Avance III HDX 600 spectrometer (Bruker Corporation, Billerica, MA, USA). Deuterated chlorofrom (Merck KGaA, Darmstadt, Germany) was used to dissolve the samples. The residual solvent shift was utilized to calibrate the chemical shift in relation to tetramethylsilane (TMS) as δ [ppm] = 0 [52]. The following abbreviations were utilized to describe multiplicities: s (singlet), d (doublet), q (quartet), m(multiplet), and bs (broad singlet). NMR spectra were analyzed using MestReNova v6.0.2 (Mestrelab Research S.L., Santiago de Compostela, Spain).

MS was performed as TLC‐MS and was recorded on an Advion TLC‐MS interface (Advion, Ithaca, NY, USA) based on electrospray ionization in positive or negative mode for most of the intermediates. The additional settings were 3.50 kV for the ESI voltage, the capillary voltage was 187 V, the source voltage was 44 V, the capillary temperature was 250°C, the desolvation gas temperature was also 250°C, and the gas flow was 5.0 L/min nitrogen. High‐resolution MS experiments were carried out on a Bruker maXIs 4 G ESI‐TOF (Bruker Corporation, Billerica, MA, USA) using positive electrospray ionization (HR‐ESI‐TOF) coupled with an UltiMate 3000 HPLC system (Thermo Fisher Scientific Inc, Waltham, MA, USA). The additional settings were 200°C for the dry heater, 1.2 bar for the nebulizer, the dry gas flow was 6.0 L/min, the capillary voltage was 4500 V, and the endplate voltage Offset was −500 V. Compounds **7a**, **7h**, and **8i** were measured as previously described without any dilution [53].

The purity of the final compounds was determined by high‐performance liquid chromatography (HPLC) on an Agilent 1100 series, including injection module, ColCom setup, degasser, and binary pump (Agilent Technologies Inc., Santa Clara, CA, USA) coupled to a 1260 DAD detector module. The system was equipped with a Phenomenex Kinetex 2.6 µm C8 100 Å 150 × 4.6 mm column (Phenomenex Inc., Torrance, CA, USA). A flow rate of either 0.5 mL/min or 1.5 mL/min at 23°C was used with an injection volume of 5 or 10 µL. Elution was performed with the following gradients: 0.01 M KH_2_PO_4_ pH 2.3 (solvent A) and MeOH (solvent B).

Method A = 0 min: 40% B/60% A, 9 min: 95% B/5% A, 10 min: 95% B/5% A, 11 min: 40% B/60% A, 16 min: 40% B/60% A.

Method B = 0 min: 40% B/60% A, 15 min: 85% B/15% A, 20 min: 85% B/15% A, 22 min: 40% B/60% A, 28 min: 40% B/60% A.

Method C = 0 min: 40% B/60 A, 8 min: 85% B/15% A, 13 min: 85% B/15% A, 14 min: 40% B/60% A, 16 min: 40% B/60% A.

Absorptions were detected at 254 and 280 nm. Unless otherwise stated, the final compounds all showed a purity above 95% according to the peak areas at the two different wavelengths.

The InChl codes of the newly investigated compounds are provided in the Supporting Information.

Unless otherwise stated, all synthetic procedures were taken and adapted from either Innocenti et al. [34], Wolfe et al. [35], or Wydra et al. [33], who had also synthesized compound **8a** as a potential MPS1 inhibitor.

#### 4.1.2 General Synthesis of the Suzuki Coupling for 3‐Chloro‐Isoquinoline Derivatives (Compound 3)

This synthesis was performed after a modified protocol of Innocenti et al. [34]. 3‐Chloro‐5‐bromoisoquinoline (1 eq.) was stirred with the boronic acid ester (1.2 equivalent (eq.)), Pd(dppf)Cl_2_ (0.03 eq.) and Na_2_CO_3_ (2 eq.) in 3 mL of a 1,4‐dioxane/water mixture (3:1) at 105°C for 18 h. Argon was bubbled through the solvent mixture before stirring. The reaction was transferred to a separatory funnel, and ammonium chloride was added. The aqueous phase was extracted with EtOAc (3×), and the organic phases were dried over Na_2_SO_4_. The crude product was concentrated under reduced pressure and purified using flash chromatography (silica gel, EtOAc/hexane), resulting in yields of 37%–93%.

#### 4.1.3 General Synthesis of Buchwald–Hartwig Aryl Amination and Hydrolysis for Isoquinoline‐3‐Amine Derivatives (Compound 5)

The amino group in position 3 of the isoquinoline core was introduced following a modified protocol of Wolfe et al. [35]. Various 3‐chloro‐isoquinoline derivatives (**3a–i**) (1 eq.), Pd(OAc)_2_ (0.1 eq.), BINAP (0.3 eq.), *t*‐BuONa (3 eq.) were suspended in 5 mL dry toluene and purged with argon. Afterward, benzophenone imine (2 eq. φ = 1.08 g/mL) was added, and the reaction vessel was capped and stirred at 120°C overnight (18 h). The next day, the solution was transferred to a 50 mL flask with toluene. The solvent was removed, and 12.5 mL of a 2 M HCl solution were added, and the mixture was stirred for 1 h at 75°C. The resulting suspension was washed twice with DCM, and then the aqueous phase was adjusted to pH = 8 with NaHCO_3_ and extracted three times with EtOAc. The organic phases were combined, dried over Na_2_SO_4_, and concentrated under reduced pressure. The raw product was purified using flash chromatography (silica gel, EtOAc/MeOH or hexane/EtOAc), resulting in yields of 5%–90%.

#### 4.1.4 General Synthesis of *N*‐(6‐Fluoro‐3‐Nitropyridin‐2‐yl)‐Isoquinolin‐3‐Amine (7a–i) and *N*‐(6‐Chloro‐3‐Nitropyridin‐2‐yl)‐Isoquinolin‐3‐Amine Derivatives (8a–e, 8g–i) by Nucleophilic Aromatic Substitution

The nucleophilic aromatic substitution was performed after the protocol of Wydra et al. [33]. The corresponding arylamines (**5a–i**) (1 eq.) and the commercially available warhead, namely 2,6‐difluoro‐3‐nitropyridine or 2,6‐dichloro‐3‐nitropyridin (2 eq.), were dissolved in 3 mL of dry 1,4‐dioxane. Subsequently, DIPEA (7 eq., φ = 0.76 g/mL) was added to the solution, and the mixture was stirred for 20 h at 101°C. The solvent was removed under vacuum. The resulting crude product (**7a–i** and **8a–e**, **8g–i**) was purified via flash column chromatography (silica gel, Hexane/EtOAc), and the obtained solid was dried under high vacuum, resulting in a yield of 3%–53%.

*N*‐(6‐Fluoro‐3‐nitropyridin‐2‐yl)‐5‐(1‐methyl‐1*H*‐pyrazol‐4‐yl)isoquinolin‐3‐amine (**7a**): Compound **7a** was prepared according to all named general synthesis procedures. The crude product (**7a**) was purified via flash chromatography (silica gel, EtOAc/hexane; 30:70 to 100:0) to yield 60 mg (82.1%) of an orange‐red solid, which was further washed with pentane and dried in high vacuum. ^1^H NMR (400 MHz, CDCl_3_) *δ* 10.93 (s, 1H, NH), 9.11 (s, 1H, Ar‐H), 9.08 (s, 1H, Ar‐H), 8.73 (dd, *J* = 8.6, 7.3 Hz, 1H, Ar‐H), 7.99 (s, 1H, Ar‐H), 7.89–7.84 (m, 2H, Ar‐H), 7.73 (d, *J* = 6.9 Hz, 1H), 7.54 (t, *J* = 7.6 Hz, 1H), 6.52 (dd, *J* = 8.8, 3.7 Hz, 1H, Ar‐H), 4.08 (s, 3H, CH_3_); ^13^C NMR (101 MHz, CDCl_3_) *δ* 164.2 (d, *J* = 248.9 Hz), 152.1, 148.5 (d, *J* = 21.1 Hz), 146.4, 141.7 (d, *J* = 11.7 Hz), 139.2, 135.6, 130.4, 130.3, 129.7, 127.2, 127.0 (d, *J* = 4.5 Hz), 126.5, 126.2, 119.9, 107.7, 99.9 (d, *J* = 40.3 Hz), 39.2; SWATH‐MS: *m/z* calcd for C_18_H_13_FN_6_O_2_ [M+H]^+^: 365.1162. Found: 365.1152 m/z; HPLC: *t*_ret_ = 8.722 min, purity: 96.53% (254 nm), 96.31% (230 nm) (method C).

*N*‐(6‐Chloro‐3‐nitropyridin‐2‐yl)‐5‐(1‐methyl‐1*H*‐pyrazol‐4‐yl)isoquinolin‐3‐amine (**8a**): Compound **8a** was the same compound as Wydra et al. published [33]. The resulting crude product (**8a**) was purified via flash column chromatography (silica gel, EtOAc/hexane, gradient elution from 40:60 to 100:0) and further washed with pentane and dried in high vacuum. The yield was 47 mg (69%) as a carmine‐colored powder. ^1^H NMR (400 MHz, CDCl3) *δ* 10.84 (s, 1H, Ar‐H), 9.06 (s, 1H, Ar‐H), 9.01 (s, 1H, Ar‐H), 8.52 (d, *J* = 8.6 Hz, 1H, Ar‐H), 7.89 (s, 1H, Ar‐H), 7.88 (s, 1H, Ar‐H), 7.86 (d, *J* = 8.5 Hz, 1H, Ar‐H), 7.69 (d, *J* = 7.0 Hz, 1H, Ar‐H), 7.52 (t, *J* = 7.6 Hz, 1H, Ar‐H), 6.90 (d, *J* = 8.6 Hz, 1H, Ar‐H), 4.05 (s, 3H, CH_3_); ^13^C NMR (101 MHz, CDCl_3_) *δ* 155.6, 151.7, 147.9, 146.2, 139.4, 138.1, 135.9, 131.1, 130.3, 129.6, 128.1, 127.0, 126.8, 126.1, 119.9, 114.9, 107.5, 39.4. HRMS ESI‐TOF: *m/z* calcd for C_18_H_13_ClN_6_O_2_ [M+Na]^+^: 403.06807. Found: 403.06856 m/z; HPLC: *t*_ret_ = 9.050 min, purity: 98.2% (254 nm), 98.2% (230 nm) (method C).

*N*‐(6‐Fluoro‐3‐nitropyridin‐2‐yl)‐5‐(1‐methyl‐1*H*‐pyrrol‐3‐yl)isoquinolin‐3‐amine (**7b**): Compound **7b** was prepared according to all named general synthesis procedures. The crude product (**7b**) was purified using flash chromatography (silica gel, EtOAc/hexane from 0:100 to 35:65) to yield 29.5 mg (40%) as a red solid. ^1^H NMR (400 MHz, CDCl_3_) δ 10.92 (s, 1H, NH), 9.27 (s, 1H, Ar‐H), 9.06 (s, 1H, Ar‐H), 8.71 (dd, *J* = 9, 7 Hz, 1H, Ar‐H), 7.80 (d, *J* = 8 Hz, 1H, Ar‐H), 7.74 (dd, *J* = 7, 1 Hz, 1H, Ar‐H), 7.52 (dd, *J* = 8, 7 Hz, 1H, Ar‐H), 7.21 (t, *J* = 2 Hz, 1H, Ar‐H), 6.79 (t, *J* = 2 Hz, 1H, Ar‐H), 6.57 (dd, *J* = 3, 2 Hz, 1H, Ar‐H), 6.48 (dd, *J* = 9, 4 Hz, 1H, Ar‐H), 3.82 (s, 3H, CH_3_). ^13^C NMR (101 MHz, CDCl_3_) δ 164.2 (d, *J* = 248 Hz), 151.8, 148.4 (d, *J* = 21 Hz), 145.9, 141.5 (d, *J* = 12 Hz), 135.6, 134.0, 130.0, 127.4, 126.8, 126.3, 125.3, 122.5, 121.9, 121.6, 109.5, 108.6, 99.6 (d, *J* = 41 Hz), 36.4. HRMS ESI‐TOF: *m/z* calcd for C_19_H_15_FN_5_O_2_ [M+H]^+^: 364.12043. Found: 364.12117. HPLC *t*_ret_: 14.51 min, purity: 99.7% (254 nm), 97.8% (230 nm) (method A); *t*_ret_: 20.51 min (method B).

*N*‐(6‐Chloro‐3‐nitropyridin‐2‐yl)‐5‐(1‐methyl‐1*H*‐pyrrol‐3‐yl)isoquinolin‐3‐amine (**8b**): Compound **8b** was prepared according to all named general synthesis procedures. The crude product (**8b**) was purified using flash chromatography (silica gel, EtOAc/hexane from 0:100 to 35:65) to yield 31.4 mg(41%) as a light red solid. ^1^H NMR (400 MHz, CDCl_3_) *δ* 10.83 (s, 1H, NH), 9.19 (s, 1H, Ar‐H), 9.07 (s, 1H, Ar‐H), 8.52 (d, *J* = 9 Hz, 1H, Ar‐H), 7.81 (d, *J* = 8 Hz, 1H, Ar‐H), 7.71 (dd, *J* = 7, 1 Hz, 1H, Ar‐H), 7.51 (dd, *J* = 8, 7 Hz, 1H, Ar‐H), 7.09 (t, *J* = 2 Hz, 1H, Ar‐H), 6.88 (d, *J* = 9 Hz, 1H, Ar‐H), 6.77 (t, *J* = 2 Hz, 1H, Ar‐H), 6.62 (dd, *J* = 3, 2 Hz, 1H, Ar‐H), 3.79 (s, 3H, CH_3_). ^13^C NMR (101 MHz, CDCl_3_) δ 155.8, 151.9, 148.1, 146.1, 138.0, 135.9, 134.1, 130.3, 127.9, 127.4, 126.1, 125.6, 122.6, 122.1, 121.3, 114.6, 110.1, 108.3, 36.6. HRMS ESI‐TOF: *m/z* calcd for C_19_H_15_ClN_5_O_2_ [M+H]^+^: 380.09088. Found: 380.09150. HPLC *t*_ret_: 13.71 min (method A); *t*_ret_: 21.03 min, purity: 99.9% (254 nm), 99.9% (230 nm) (method B).

5‐(1‐Ethyl‐1*H*‐pyrazol‐4‐yl)‐*N*‐(6‐fluoro‐3‐nitropyridin‐2‐yl)isoquinolin‐3‐amine (**7c**): Compound **7c** was prepared according to all named general synthesis procedures. The crude product (**7c**) was purified using flash chromatography (silica gel, EtOAc/hexane 10:90 to 70:30) to provide 12 mg (38%) as a brick red solid. ^1^H NMR (400 MHz, CDCl_3_) *δ* 10.91 (s, 1H, NH), 9.07 (s, 2H, Ar‐H), 8.72 (dd, *J* = 9, 7 Hz, 1H, Ar‐H), 7.99 (s, 1H, Ar‐H), 7.89–7.84 (m, 2H, Ar‐H), 7.73 (dd, *J* = 7, 1 Hz, 1H, Ar‐H), 7.53 (dd, *J* = 8, 7 Hz, 1H, Ar‐H), 6.50 (dd, *J* = 9, 4 Hz, 1H, Ar‐H), 4.34 (q, *J* = 7 Hz, 2H, CH_2_), 1.61 (t, *J* = 7 Hz, 3H, CH_3_). ^13^C NMR (101 MHz, CDCl_3_) δ 164.2 (d, *J* = 249 Hz), 152.1, 148.5 (d, *J* = 21 Hz), 146.4, 141.7 (d, *J* = 12 Hz), 139.1, 135.6, 130.5, 130.4, 128.1, 127.2, 127.0 (d, *J* = 5 Hz), 126.5, 126.2, 119.6, 107.7, 99.9 (d, *J* = 40 Hz), 47.4, 15.7. HRMS ESI‐TOF: *m/z* calcd for C_19_H_16_FN_6_O_2_ [M+H]^+^: 379.13133. Found: 379.13182. HPLC *t*_ret_: 12.83 min (method A); *t*_ret_: 18.94 min, purity: 98.8% (254 nm), 95.9% (230 nm) (method B).

*N*‐(6‐Chloro‐3‐nitropyridin‐2‐yl)‐5‐(1‐ethyl‐1*H*‐pyrazol‐4‐yl)isoquinolin‐3‐amine (**8c**): Compound **8c** was prepared according to all named general synthesis procedures. The crude product (**8c**) was purified using flash chromatography (silica gel, EtOAc/hexane 10:90 to 50:50) to provide 14 mg (43%) as a red solid. ^1^H NMR (400 MHz, CDCl_3_) *δ* 10.81 (s, 1H, NH), 9.07 (s, 1H, Ar‐H), 9.02 (s, 1H, Ar‐H), 8.52 (d, *J* = 9 Hz, 1H, Ar‐H), 7.92 (s, 1H, Ar‐H), 7.89–7.85 (m, 2H, Ar‐H), 7.69 (dd, *J* = 7, 1 Hz, 1H, Ar‐H), 7.55– 7.49 (m, 1H, Ar‐H), 6.90 (d, *J* = 9 Hz, 1H, Ar‐H), 4.31 (q, *J* = 7 Hz, 2H, CH_2_), 1.59 (t, *J* = 7 Hz, 3H, CH_3_). ^13^C NMR (101 MHz, CDCl_3_) *δ* 155.8, 152.1, 148.1, 146.5, 139.3, 138.1, 135.9, 130.9, 130.5, 128.1, 128.0, 127.2, 126.7, 126.0, 119.7, 114.8, 107.5, 47.4, 15.8. HRMS ESI‐TOF: *m/z* calcd for C_19_H_16_ClN_6_O_2_ [M+H]^+^: 395.10178. Found: 395.10240. HPLC *t*_ret_: 13.07 min, purity: 100.0% (254 nm), 100.0% (230 nm) (method A); *t*_ret_: 19.63 min (method B).

*N*‐(6‐Fluoro‐3‐nitropyridin‐2‐yl)‐5‐(1‐propyl‐1*H*‐pyrazol‐4‐yl)isoquinolin‐3‐amine (**7d**): Compound **7d** was prepared according to all named general synthesis procedures. The crude product (**7d**) was purified using flash chromatography (silica gel, EtOAc/hexane 10:90 to 60:40) to provide 9 mg (13%) as a dark orange solid. ^1^H NMR (400 MHz, CDCl_3_) *δ* 10.94 (s, 1H, NH), 9.09 (d, *J* = 3 Hz, 2H, Ar‐H), 8.74 (dd, *J* = 9, 7 Hz, 1H, Ar‐H), 7.99 (s, 1H, Ar‐H), 7.91–7.86 (m, 2H, Ar‐H), 7.75 (d, *J* = 6 Hz, 1H, Ar‐H), 7.57–7.51 (m, 1H, Ar‐H), 6.52 (dd, *J* = 9, 4 Hz, 1H, Ar‐H), 4.24 (t, *J* = 7 Hz, 2H, CH_2_), 2.07–1.96 (m, 2H, CH_2_), 1.00 (t, *J* = 7 Hz, 3H, CH_3_). ^13^C NMR (101 MHz, CDCl_3_) *δ* 163.0, 152.1, 148.5 (d, *J* = 21 Hz), 146.4, 141.7 (d, *J* = 12 Hz), 139.1, 135.6, 130.5, 130.4, 128.8, 127.3, 127.1, 126.5, 126.2, 119.5, 107.9, 99.9 (d, *J* = 40 Hz), 54.2, 24.0, 11.4. HRMS ESI‐TOF: *m/z* calcd for C_20_H_18_FN_6_O_2_ [M+H]^+^: 393.14698. Found: 393.14722. HPLC *t*_ret_: 13.17 min, purity: 95.3% (254 nm), 96.9% (230 nm) (method A); *t*_ret_: 19.78 min (method B).

*N*‐(6‐Chloro‐3‐nitropyridin‐2‐yl)‐5‐(1‐propyl‐1*H*‐pyrazol‐4‐yl)isoquinolin‐3‐amine (**8d**): Compound **8d** was prepared according to all named general synthesis procedures. The crude product (**8d**) was purified using flash chromatography (silica gel, EtOAc/hexane 10:90 to 40:60) to provide 16 mg (22%) as a dark yellow solid. ^1^H NMR (400 MHz, CDCl_3_) *δ* 10.82 (s, 1H, NH), 9.08 (s, 1H, Ar‐H), 9.02 (s, 1H, Ar‐H), 8.53 (d, *J* = 9 Hz, 1H, Ar‐H), 7.93 (s, 1H, Ar‐H), 7.87 (d, *J* = 9 Hz, 2H, Ar‐H), 7.70 (d, *J* = 7 Hz, 1H, Ar‐H), 7.53 (t, *J* = 8 Hz, 1H, Ar‐H), 6.91 (d, *J* = 9 Hz, 1H, Ar‐H), 4.22 (t, *J* = 7 Hz, 2H, CH_2_), 2.06–1.94 (m, 2H, CH_2_), 0.99 (t, *J* = 7 Hz, 3H, CH_3_). ^13^C NMR (101 MHz, CDCl_3_) *δ* 155.8, 152.1, 148.1, 146.5, 139.3, 138.1, 135.9, 130.9, 130.5, 128.6, 128.0, 127.2, 126.6, 126.0, 119.6, 114.8, 107.6, 54.3, 24.1, 11.4. HRMS ESI‐TOF: *m/z* calcd for C_20_H_18_ClN_6_O_2_ [M+H]^+^: 409.11743. Found: 409.11784. HPLC *t*_ret_: 13.37 min, purity: 97.8% (254 nm), 97.6% (230 nm) (method A); *t*_ret_: 20.14 min (method B).

5‐(1‐Cyclopropyl‐1*H*‐pyrazol‐4‐yl)‐*N*‐(6‐fluoro‐3‐nitropyridin‐2‐yl)isoquinolin‐3‐amine (**7e**): Compound **7e** was prepared according to all named general synthesis procedures. The crude product (**7e**) was purified using flash chromatography (silica gel, EtOAc/hexane 10:90 to 65:35) to provide 18 mg (23%) as a red solid. ^1^H NMR (400 MHz, CDCl_3_) *δ* 0.93 (s, 1H, NH), 9.08 (s, 1H, Ar‐H), 9.06 (s, 1H, Ar‐H), 8.73 (dd, *J* = 9, 7 Hz, 1H, Ar‐H), 8.03 (s, 1H, Ar‐H), 7.89–7.84 (m, 2H, Ar‐H), 7.71 (dd, *J* = 7, 1 Hz, 1H, Ar‐H), 7.53 (dd, *J* = 8, 7 Hz, 1H, Ar‐H), 6.51 (dd, *J* = 9, 4 Hz, 1H, Ar‐H), 3.79–3.73 (m, 1H, CH), 1.29–1.26 (m, 2H, CH_2_), 1.13–1.08 (m, 2H, CH_2_). ^13^C NMR (101 MHz, CDCl_3_) δ 164.3 (d, *J* = 250 Hz), 152.1, 148.5 (d, *J* = 21 Hz), 146.4, 141.7 (d, *J* = 12 Hz), 139.2, 135.7, 130.6, 130.3, 129.3, 127.2, 127.0 (d, *J* = 5 Hz), 126.6, 126.1, 119.6, 107.7, 99.9 (d, *J* = 40 Hz), 33.1, 6.7. HRMS ESI‐TOF: *m/z* calcd for C_20_H_16_FN_6_O_2_ [M+H]^+^: 391.13133. Found: 391.13166. HPLC *t*_ret_: 12.98 min, purity: 98.4% (254 nm), 100.0% (230 nm) (method A); *t*_ret_: 19.41 min (method B).

*N*‐(6‐Chloro‐3‐nitropyridin‐2‐yl)‐5‐(1‐cyclopropyl‐1*H*‐pyrazol‐4‐yl)isoquinolin‐3‐amine (**8e**): Compound **8e** was prepared according to all named general synthesis procedures. The crude product (**8e**) was purified using flash chromatography (silica gel, EtOAc/hexane 10:90 to 60:40) to provide 43 mg (53%) as an orange solid. ^1^H NMR (400 MHz, CDCl_3_) *δ* 10.82 (s, 1H, NH), 9.07 (s, 1H, Ar‐H), 9.03 (s, 1H, Ar‐H), 8.52 (d, *J* = 9 Hz, 1H, Ar‐H), 7.95 (s, 1H, Ar‐H), 7.89–7.85 (m, 2H, Ar‐H), 7.67 (dd, *J* = 7, 1 Hz, 1H, Ar‐H), 7.54–7.48 (m, 1H, Ar‐H), 6.90 (d, *J* = 9 Hz, 1H, Ar‐H), 3.76–3.69 (m, 1H, CH), 1.27–1.23 (m, 2H, CH_2_), 1.11–1.05 (m, 2H, CH_2_). ^13^C NMR (101 MHz, CDCl_3_) *δ* 155.9, 152.1, 148.0, 146.6, 139.4, 138.1, 136.0, 131.0, 130.4, 129.3, 128.0, 127.2, 126.7, 126.0, 119.7, 114.8, 107.5, 33.2, 6.9. HRMS ESI‐TOF: *m/z* calcd for C_20_H_16_ClN_6_O_2_ [M+H]^+^: 407.10178. Found: 407.10208. HPLC *t*_ret_: 13.25 min (method A); *t*_ret_: 19.87 min, purity: 98.8% (254 nm), 100.0% (230 nm) (method B).

2‐(4‐{3‐[(6‐Fluoro‐3‐nitropyridin‐2‐yl)amino]isoquinolin‐5‐yl}‐1*H*‐pyrazol‐1‐yl)ethan‐1‐ol (**7f**): Compound **7f** was prepared according to all named general synthesis procedures. The crude product (**7f**) was purified using flash chromatography (silica gel, EtOAc/hexane 10:90 to 60:40) to provide 3 mg (28%) as a carmine red solid. ^1^H NMR (400 MHz, CDCl_3_) *δ* 10.95 (s, 1H, NH), 9.10 (s, 2H, Ar‐H), 8.74 (dd, *J* = 9, 7 Hz, 1H, Ar‐H), 8.04 (s, 1H, Ar‐H), 7.92 (s, 1H, Ar‐H), 7.89 (d, *J* = 8 Hz, 1H, Ar‐H), 7.74 (d, *J* = 7 Hz, 1H, Ar‐H), 7.56 (d, *J* = 7 Hz, 1H, Ar‐H), 6.52 (dd, *J* = 9, 4 Hz, 1H, Ar‐H), 4.44–4.41 (m, 2H, CH_2_), 4.15–4.12 (m, 2H, CH_2_). ^13^C NMR (151 MHz, CDCl_3_) *δ* 164.1 (d, *J* = 244 Hz), 152.1, 148.3 (d, *J* = 18 Hz), 146.3, 141.8 (d, *J* = 11 Hz), 139.7, 135.5, 130.6, 129.9, 129.8, 127.1, 126.9, 126.7, 126.2, 119.7, 107.6, 100.0 (d, *J* = 40 Hz), 62.2, 54.0. HRMS ESI‐TOF: *m/z* calcd for C_19_H_16_FN_6_O_3_ [M+H]^+^: 395.12624. Found: 395.12677. HPLC *t*_ret_: 11.54 min, purity: 91.5% (254 nm), 93.8% (230 nm) (method A); *t*_ret_: 16.33 min (method B).

*N*‐(6‐Fluoro‐3‐nitropyridin‐2‐yl)‐5‐(naphthalen‐2‐yl)isoquinolin‐3‐amine (**7g**): Compound **7g** was prepared according to all named general synthesis procedures. The crude product (**7g**) was purified using flash chromatography (silica gel, EtOAc/hexane 0:100 to 35:65) to provide 47.2 mg (52%) as a brick red solid. ^1^H NMR (400 MHz, CDCl_3_) *δ* 10.82 (s, 1H, NH), 9.14 (s, 1H, Ar‐H), 8.86 (s, 1H, Ar‐H), 8.63 (dd, *J* = 9, 7 Hz, 1H, Ar‐H), 8.07 (s, 1H, Ar‐H), 8.04 (d, *J* = 8 Hz, 1H, Ar‐H), 8.00–7.93 (m, 3H, Ar‐H), 7.81–7.76 (m, 2H, Ar‐H), 7.65–7.60 (m, 1H, Ar‐H), 7.56 (dd, *J* = 6, 3 Hz, 2H, Ar‐H), 6.37 (dd, *J* = 9, 4 Hz, 1H, Ar‐H). ^13^C NMR (101 MHz, CDCl_3_) *δ* 164.2 (d, *J* = 251 Hz), 152.0, 148.6 (d, *J* = 21 Hz), 146.3, 141.4 (d, *J* = 12 Hz), 139.4, 136.7, 136.2, 133.7, 133.0, 131.9, 129.0, 128.5, 128.3, 128.2, 127.9, 127.3, 127.2, 126.7 (d, *J* = 5 Hz), 126.4, 126.4, 126.1, 108.6, 100.1 (d, *J* = 40 Hz). HRMS ESI‐TOF: *m/z* calcd for C_24_H_16_FN_4_O_2_ [M+H]^+^: 411.12518. Found: 411.12555. HPLC *t*_ret_: 14.51 min (method A); *t*_ret_: 24.47 min, purity: 99.1% (254 nm), 98.6% (230 nm) (method B).

*N*‐(6‐Chloro‐3‐nitropyridin‐2‐yl)‐5‐(naphthalen‐2‐yl)isoquinolin‐3‐amine (**8g**): Compound **8g** was prepared according to all named general synthesis procedures. The crude product (**8g**) was purified using flash chromatography (silica gel, EtOAc/hexane 0:100 to 35:65) to provide 45.1 mg (48%) as a brick red solid. ^1^H NMR (400 MHz, CDCl_3_) *δ* 10.77 (s, 1H, NH), 9.14 (s, 1H, Ar‐H), 8.85 (s, 1H, Ar‐H), 8.43 (d, *J* = 9 Hz, 1H, Ar‐H), 8.00 (t, *J* = 10 Hz, 3H, Ar‐H), 7.93 (d, *J* = 6 Hz, 2H, Ar‐H), 7.78–7.71 (m, 2H, Ar‐H), 7.61 (t, *J* = 8 Hz, 1H, Ar‐H), 7.54 (dd, *J* = 9, 5 Hz, 2H, Ar‐H), 6.73 (d, *J* = 9 Hz, 1H, Ar‐H). ^13^C NMR (101 MHz, CDCl_3_) *δ* 156.0, 152.0, 147.9, 146.7, 139.7, 137.8, 137.0, 136.6, 133.7, 133.0, 131.8, 128.9, 128.5, 128.0, 127.8, 127.3, 127.0, 126.4, 125.8, 114.7, 107.9. HRMS ESI‐TOF: *m/z* calcd for C_24_H_16_ClN_4_O_2_ [M+H]^+^: 427.09563. Found: 427.09635. HPLC *t*_ret_: 14.73 min (method A); *t*_ret_: 25.17 min, purity: 96.8% (254 nm), 97.6% (230 nm) (method B).

*N*‐(6‐Fluoro‐3‐nitropyridin‐2‐yl)‐5‐[4‐(pyridin‐3‐yl)phenyl]isoquinolin‐3‐amine (**7h**): Compound **7h** was prepared according to all named general synthesis procedures. The crude product (**7h**) was purified twice using flash chromatography (silica gel, EtOAc/hexane 10:90 to 60:40 and DCM/MeOH 100:0 to 90:10) to provide 3 mg (3%) as a yellow beige solid. ^1^H NMR (400 MHz, CDCl_3_) *δ* 10.88 (s, 1H, NH), 9.14 (s, 1H, Ar‐H), 8.98 (s, 1H, Ar‐H), 8.94 (s, 1H, Ar‐H), 8.73–8.63 (m, 2H, Ar‐H), 7.99 (dd, *J* = 8.1, 1.2 Hz, 2H, Ar‐H), 7.78 (d, *J* = 6.3 Hz, 4H, Ar‐H), 7.62 (dd, *J* = 8.1, 7.2 Hz, 1H, Ar‐H), 7.53 (dd, *J* = 5.3, 2.9 Hz, 1H, Ar‐H), 7.44 (dd, *J* = 7.7, 4.9 Hz, 1H, Ar‐H), 6.46 (dd, *J* = 8.9, 3.8 Hz, 1H, Ar‐H). ^13^C NMR (101 MHz, CDCl_3_) N/A due to less amount; SWATH‐MS: *m/z* calcd for C_25_H_16_FN_5_O_2_ [M+H]^+^: 438.1361. Found: 438.1375. HPLC *t*_ret_: 14.09 min, purity: 98.6% (254 nm), 88.3% (230 nm) (method A) *t*_ret_: 21.68 min (method B).

*N*‐(6‐Chloro‐3‐nitropyridin‐2‐yl)‐5‐[4‐(pyridin‐3‐yl)phenyl]isoquinolin‐3‐amine (**8h**): Compound **8h** was prepared according to all named general synthesis procedures. The crude product (**8h**) was purified using flash chromatography (silica gel, EtOAc/hexane 10:90 to 35:65) to provide 10 mg (11%) as a pale yellow solid. ^1^H NMR (400 MHz, CDCl_3_) *δ* 10.80 (s, 1H, NH), 9.15 (s, 1H, Ar‐H), 8.95 (s, 1H, Ar‐H), 8.88 (s, 1H, Ar‐H), 8.65 (d, *J* = 4.9 Hz, 1H, Ar‐H), 8.49 (d, *J* = 8.6 Hz, 1H, Ar‐H), 8.00–7.95 (m, 2H, Ar‐H), 7.79 (d, *J* = 8.3 Hz, 2H, Ar‐H), 7.75–7.71 (m, 3H, Ar‐H), 7.61 (s, *J* = 8.0 Hz, 1H, Ar‐H), 7.45–7.42 (m, 1H, Ar‐H), 6.84 (d, *J* = 8.6 Hz, 1H, Ar‐H). ^13^C NMR (101 MHz, CDCl_3_) N/A due to less amount; HRMS ESI‐TOF: *m/z* calcd for C_25_H_16_ClN_5_O_2_ [M+H]^+^: 454.10653. Found: 454.10691. HPLC *t*_ret_: 14.09 min, purity: 95.9% (254 nm), 92.9% (230 nm) (method A) *t*_ret_: 22.72 min (method B).

*N*‐(6‐Fluoro‐3‐nitropyridin‐2‐yl)‐5‐[4‐(pyridin‐4‐yl)phenyl]isoquinolin‐3‐amine (**7i**): Compound **7i** was prepared according to all named general synthesis procedures. The crude product (**7i**) was purified using flash chromatography (silica gel, EtOAc/hexane 10:90 to 40:60) to provide 5 mg (16%) as a dark red solid. ^1^H NMR (400 MHz, CDCl3) *δ* 10.88 (s, 1H, NH), 9.14 (d, *J* = 0.7 Hz, 1H, Ar‐H), 8.92 (s, 1H, Ar‐H), 8.73 (d, *J* = 6.0 Hz, 2H, Ar‐H), 8.69–8.66 (m, 1H, Ar‐H), 7.99 (t, *J* = 6.9 Hz, 1H, Ar‐H), 7.86 (d, *J* = 8.2 Hz, 2H, Ar‐H), 7.79 (s, 1H, Ar‐H), 7.76 (d, *J* = 1.4 Hz, 1H, Ar‐H), 7.63 (dd, *J* = 4.5, 1.6 Hz, 4H, Ar‐H), 6.47 (dd, *J* = 8.9, 3.8 Hz, 1H, Ar‐H). ^13^C NMR (101 MHz, CDCl_3_) N/A due to less amount; HRMS ESI‐TOF: *m/z* calcd for C_25_H_16_FN_5_O_2_ [M+H]^+^: 438.13608 Found: 438.13646. HPLC *t*_ret_: 13.99 min, purity: 98.6% (254 nm), 100.0% (230 nm) (Method A); *t*_ret_: 21.52 min (method B).

*N*‐(6‐Chloro‐3‐nitropyridin‐2‐yl)‐5‐[4‐(pyridin‐4‐yl)phenyl]isoquinolin‐3‐amine (**8i**): Compound **8i** was prepared according to all named general synthesis procedures. The crude product (**8i**) was purified using flash chromatography (silica gel, EtOAc/hexane 10:90 to 65:35) to provide 7 mg (24%) as a red solid. ^1^H NMR (400 MHz, CDCl3) *δ* 10.81 (s, 1H, NH), 9.15 (s, 1H, Ar‐H), 8.87 (s, 1H, Ar‐H), 8.72 (d, *J* = 5.9 Hz, 2H, Ar‐H), 8.50 (d, *J* = 8.6 Hz, 1H, Ar‐H), 8.00 (d, *J* = 8.2 Hz, 1H, Ar‐H), 7.84 (d, *J* = 8.3 Hz, 2H, Ar‐H), 7.74 (s, 1H, Ar‐H), 7.72 (s, 1H, Ar‐H), 7.64–7.58 (m, 4H, Ar‐H), 6.84 (d, *J* = 8.6 Hz, 1H, Ar‐H). ^13^C NMR (101 MHz, CDCl_3_) N/A due to less amount SWATH‐MS: *m/z* calcd for C_25_H_16_ClN_5_O_2_ [M+H]^+^: 454.1065 Found: 454.1049. HPLC *t*_ret_: 14.05 min (Method A); *t*_ret_: 22.30 min, purity: 98.4% (254 nm), 95.2% (230 nm) (method B).

### 4.2 Biophysical Methods

#### 4.2.1 Molecular Biology

The expression and purification of USP7 catalytic domain (USP7_CD_) (208‐560) and USP7asoc (active site only cysteine) by using a pET24a(+)_HLT_USP7 construct were performed as previously described [30, 31]. The purity and the correct protein mass of USP7 were confirmed by sodium dodecyl sulfate polyacrylamide gel electrophoresis (SDS‐PAGE) and by ultrahigh performance liquid chromatography electrospray ionization mass spectrometry (UHPLC‐ESI‐MS) (Supporting Information S2: Figure S3.1). For the USP7asoc, six of the seven cysteines are mutated to serines (208‐560, C300/315/334/448/478/510S). All utilized protein sequences are depicted in Supporting Information S2: Table S.3.1.

#### 4.2.2 DSF

The melting temperatures of USP7 and USP7asoc in the presence or absence of fragments were determined by DSF on a Qiagen Rotor‐Q Model‐5‐Plex HRM real‐time PCR instrument (Qiagen, Hilden, Germany). SYPRO Orange (Life Technologies Corporation, Eugene, OR, USA) was used as a fluorescent dye at a final concentration of 5x. The protein concentration was 8 µM, and the final compound concentration was 250 µM, corresponding to a protein‐to‐compound ratio of 1:31.25. All compounds were dissolved in dimethyl sulfoxide (DMSO) and the proteins in Tris buffer (25 mM Tris pH 8.0, 150 mM NaCl, 5 mM TCEP, 5% (v/v) DMSO). All measurements were performed after 30 min, 4 h, and 24 h of incubation and at least in triplicate. DSF experiments were carried out with a constant heating rate of 270°C/h [54]. The temperature was ramped from 28°C to 60°C–70°C with excitation and emission filters set at 470 nm and 610 nm, respectively [31]. The melting temperatures (*T*_m_) of USP7 and USP7asoc were determined from the maxima of the first derivatives of the melting curves in OriginPro2020 (OriginLab, Northampton, MA, USA). The ∆*T*_m_ was calculated by subtraction of the *T*_m_ of the protein sample with pure DMSO from the *T*_m_ of the protein sample mixed with compound. Error propagation was used to average multiple runs for a compound. A compound was considered to be a hit if the shift in the ∆*T*_m_ was at least 0.50°C [55].

#### 4.2.3 Intact Protein MS

USP7 was prepared in Tris buffer (25 mM Tris pH 8.0, 150 mM NaCl, 5 mM TCEP) with a protein‐to‐compound ratio of 1:31.25 and 5% (v/v) DMSO. The protein‐compound mixtures were all incubated at 20°C for 24 h on a rotating shaker. The analysis of intact protein mass was carried out using UHPLC‐ESI‐MS measurements, with data acquisition and data analysis as previously described [56].

#### 4.2.4 Protein Crystallization

For co‐crystallization experiments, purified USP7cd (17.5 mg/mL) was incubated with compound **7a** (500 µM in reservoir solution) overnight at 20°C in a rotating shaker. The next day, the protein‐compound solution was mixed with the reservoir solution (0.1 M HEPES pH 7.5, 0.2 M sodium bromide, 22% PEG3350) in an MRC Maxi 48‐Well Crystallization Plate (Jena Bioscience, Jena, Germany) and crystallized by using the sitting drop vapor‐diffusion technique and by streak seeding at 19°C within 1–2 days. Crystals were cryoprotected in reservoir solution supplemented with 20% glycerol before being flash‐frozen in liquid nitrogen for data collection.

#### 4.2.5 Data Collection and Refinement

Diffraction images were collected at the Deutsches Elektronen‐Synchrotron (DESY) (Hamburg, Germany) at the beamline PETRA III P11 with the detector Dectris EIGER2 Si 16 M. XDS (X‐ray Detector Software) was used for processing and reducing the data [57]. Molecular replacement was performed to get initial phases. The crystal structure with PDB ID 4M5X served as a search model for PHASER [58] as part of the CCP4 suite [59]. Multiple rounds of manual model building using Coot [60] and PHENIX [61] were performed for phase improvement. Covalently bound compound restraints were generated using JLigand [62] in ACEdrg mode [63]. The unbiased omit map was created by removing the covalently bound compound **7a** and all cysteine sidechain atoms, followed by refinement with simulated annealing.

#### 4.2.6 IC_50_ Determination With Ub‐AMC

The cleavage activity of USP7cd in the presence or absence of inhibitors was measured using Ubiquitin‐Aminomethylcoumarin (Ub‐AMC) (Enzo Life Sciences GmbH, Lörrach, Germany) as a fluorogenic substrate. The fluorescence was monitored using the CLARIOstar plate reader (BMG Labtech GmbH, Ortenberg, Germany) at 25°C at 360 nm excitation and 460 nm emission wavelengths. The components for the measurements were pipetted in a black nonbinding polystyrene 384‐well microplate (Greiner Bio One, Frickenhausen, Germany). Each measurement was performed at least twice in duplicate. For IC_50_ determination, USP7cd (10 nM) was pre‐incubated for 24 h at 20°C in a rotating shaker with different concentrations of inhibitors (with increasing inhibitor concentrations ranging from 450 to 0.439 µM) or DMSO as a control in 25 mM Tris pH 8.0, 150 mM NaCl, 5 mM TCEP supplemented with 0.2 mg/mL bovine serum albumin (BSA). Ub‐AMC was added to a final concentration of 500 nM, and the fluorescence was measured after 1 h. The normalized dose‐response semi‐logarithmic curves were fitted using OriginPro2021 and the four‐parameter logistic function.

#### 4.2.7 Glutathion (GSH) Assay

The stability of the compounds toward nucleophiles was evaluated through GSH reactivity studies. The protocol was adapted from Keeley et al. [64] and was slightly modified [31]. The conditions were PBS pH 7.4, 50% acetonitrile, 100 µM ketoprofen as internal standard, 100 µM fragment, and 5 mM GSH excess at 37°C. Measurement times were after 0, 1, 2, 4, 8, 12, and 24 h and as duplicates. The mixture was measured with an UltiMate 3000 HPLC‐System (Thermo Fisher Scientific, Dreieich, Germany) with UV‐detection (Thermo Fisher Scientific, Dreieich, Germany). As column served a ReproSil‐XR 120 C18, 5 µm, 150 mm × 4.6 mm column (Dr. Maisch GmbH, Ammerbuch‐Entringen, Germany) at 25°C. Mobile phase A was 0.01 M KH_2_PO_4_ pH 2.3, and mobile phase B was methanol. The injection volume was 5 µL and the flow rate was set to 0.5 mL/min with the following gradient: 0–9 min: 10%–85% B, 9–13 min: 85% B, 13–14 min: 85%–10% B, 14–18 min: 10% B. Absorptions were detected at 218, 254, and 280 nm. The reaction of the compounds was detected by measuring the decreasing area under the curve (AUC) of the fragment relative to the internal standard. The relative declining area was fitted in OriginPro2020 (OriginLab, Northampton, MA, USA) to the integrated rate equation of pseudo‐first order kinetics:

$$relative\;AUC=e^{-kt}.$$

Half‐life *t*_1/2_ was calculated from the pseudo‐first order rate constant k following the equation:

$$t_{1/2}=\frac{\ln2}{k}.$$

The GSH *t*_1/2_ values are given as the mean value of the duplicate determination with the respective standard deviation, which was calculated according to the rules of error propagation.

### 4.3 Molecular Docking

Docking was performed using the covalent docking protocol from AutoDock4 [65, 66]. Compound **7a** as well as SAR compounds **7b–7i** were docked against a high‐resolution X‐ray diffraction crystal structure of USP7 in complex with ubiquitin (PDB Code 5KYD) [32]. The receptor was prepared using the Protein Preparation Wizard of the Schrödinger Software Suite (Schrödinger Release 2024‐4) [67], reassigning bond orders, replacing hydrogens, and determining sensible protonation states at pH 7.4 using PROPKA [68]. Docking was run with 4 million maximum score evaluations per Lamarckian genetic algorithm (LGA) run, a population size of 200, a maximum of 100.000 generations per LGA run, and an AutoStop energy standard deviation tolerance of 0.1 kcal/mol. Otherwise default parameters were used.

## Conflicts of Interest

The authors declare no conflicts of interest.

## Supporting information

ArchPharm SupplMat InChI.

Synthese paper SI.

## Data Availability

The data that support the findings of this study are available in the supporting material of this article.
