# Supplementary material for: Design, Synthesis, and Molecular Evaluation of SNAr‐Reactive N‐(6‐Fluoro‐3‐Nitropyridin‐2‐yl)Isoquinolin‐3‐Amines as Covalent USP7 Inhibitors Reveals an Unconventional Binding Mode
Source: Arch Pharm (Weinheim). 2025 Aug 10;358(8):e70053. doi: 10.1002/ardp.70053 (PMC12336439; doi:10.1002/ardp.70053)
Supplement: Supplementary file 1 — ArchPharm SupplMat InChI. [file ARDP-358-e70053-s002.doc]

**Supplemental Material: Novel Compounds and Biological Screening Results**

**Design, Synthesis and Molecular Evaluation of S_N_Ar-reactive *N*-(6-Fluoro-3-nitropyridin-2-yl)isoquinolin-3-amine Derivatives as Covalent Inhibitors of the Cysteine Protease USP7 Reveals an Unconventional Binding Mode**

Larissa N. Ernst^1^, Jason Stahlecker^1^, Finn Mier^1^, Ricardo A. M. Serafim^2#^, Valentin R. Wydra^2^, Benedikt Masberg^3^, Simon J. Jaag^3^, Cornelius Knappe^3^, Michael Lämmerhofer^3^, Thilo Stehle^4^, Matthias Gehringer^2,5,6‡*^, Frank M. Boeckler^1,7‡*^

1 Department of Pharmacy and Biochemistry, Eberhard Karls Universität Tübingen, Laboratory for Molecular Design & Pharmaceutical Biophysics, Institute of Pharmaceutical Sciences, 72076 Tübingen, Germany

2 Department of Medicinal Chemistry, Eberhard Karls Universität Tübingen, Faculty of Medicine, Institute for Biomedical Engineering, 72076 Tübingen, Germany

3 Department of Pharmacy and Biochemistry, Eberhard Karls Universität, Pharmaceutical Chemistry, Institute of Pharmaceutical Sciences, 72076 Tübingen, Germany

4 Department of Pharmacy and Biochemistry, Eberhard Karls Universität, Pharmaceutical (Bio‑)Analysis, Institute of Pharmaceutical Sciences, 72076 Tübingen, Germany

5 Interfaculty Institute of Biochemistry, Eberhard Karls Universität Tübingen, 72076 Tübingen, Germany

6 Cluster of Excellence iFIT (EXC 2180) ‘Image-Guided & Functionally Instructed Tumor Therapies’, Eberhard Karls Universität Tübingen, 72076 Tübingen, Germany

7 Interfaculty Institute for Biomedical Informatics (IBMI), Eberhard Karls Universität Tübingen, 72076 Tübingen, Germany

# present address: Department of Organic and Pharmaceutical Chemistry, School of Engineering, Institut Químic de Sarrià (IQS), Universitat Ramon Llull (URL), 08017 Barcelona, Spain

‡ Shared last author

*Correspondence:

Prof. Dr. Frank M. Boeckler, Department of Pharmacy and Biochemistry, Eberhard Karls Universität Tübingen, Laboratory for Molecular Design & Pharmaceutical Biophysics, Institute of Pharmaceutical Sciences, 72076 Tübingen, Germany

Auf der Morgenstelle 8 (Haus B)

D – 72076 Tübingen

Tel +49 7071 29 74567

Email frank.boeckler@uni-tuebingen.de

Prof. Dr. Matthias Gehringer, Department of Medicinal Chemistry, Eberhard Karls Universität Tübingen, Faculty of Medicine, Institute for Biomedical Engineering, 72076 Tübingen, Germany

Auf der Morgenstelle 8 (Haus B)

D – 72076 Tübingen

Tel +49 7071 29 74582

Email matthias.gehringer@uni-tuebingen.de

| **Compound No.** | **InChI** | **Biological Activity (IC_50_)^a^** |
| --- | --- | --- |
| 7a | 1S/C18H13FN6O2/c1-24-10-12(9-21-24)13-4-2-3-11-8-20-17(7-14(11)13)23-18-15(25(26)27)5-6-16(19)22-18/h2-10H,1H3,(H,20,22,23) | 48.20 ± 6.894 |
| 8a | 1S/C18H13ClN6O2/c1-24-10-12(9-21-24)13-4-2-3-11-8-20-17(7-14(11)13)23-18-15(25(26)27)5-6-16(19)22-18/h2-10H,1H3,(H,20,22,23) | N/D |
| 7b | 1S/C19H14FN5O2/c1-24-8-7-13(11-24)14-4-2-3-12-10-21-18(9-15(12)14)23-19-16(25(26)27)5-6-17(20)22-19/h2-11H,1H3,(H,21,22,23) | 56.64 ± 6.112 |
| 8b | 1S/C19H14ClN5O2/c1-24-8-7-13(11-24)14-4-2-3-12-10-21-18(9-15(12)14)23-19-16(25(26)27)5-6-17(20)22-19/h2-11H,1H3,(H,21,22,23) | N/D |
| 7c | 1S/C19H15FN6O2/c1-2-25-11-13(10-22-25)14-5-3-4-12-9-21-18(8-15(12)14)24-19-16(26(27)28)6-7-17(20)23-19/h3-11H,2H2,1H3,(H,21,23,24) | 36.17 ± 2.206 |
| 8c | 1S/C19H15ClN6O2/c1-2-25-11-13(10-22-25)14-5-3-4-12-9-21-18(8-15(12)14)24-19-16(26(27)28)6-7-17(20)23-19/h3-11H,2H2,1H3,(H,21,23,24) | N/D |
| 7d | 1S/C20H17FN6O2/c1-2-8-26-12-14(11-23-26)15-5-3-4-13-10-22-19(9-16(13)15)25-20-17(27(28)29)6-7-18(21)24-20/h3-7,9-12H,2,8H2,1H3,(H,22,24,25) | 26.77 ± 7.544 |
| 8d | 1S/C20H17ClN6O2/c1-2-8-26-12-14(11-23-26)15-5-3-4-13-10-22-19(9-16(13)15)25-20-17(27(28)29)6-7-18(21)24-20/h3-7,9-12H,2,8H2,1H3,(H,22,24,25) | N/D |
| 7e | 1S/C20H15FN6O2/c21-18-7-6-17(27(28)29)20(24-18)25-19-8-16-12(9-22-19)2-1-3-15(16)13-10-23-26(11-13)14-4-5-14/h1-3,6-11,14H,4-5H2,(H,22,24,25) | 48.35 ± 5.677 |
| 8e | 1S/C20H15ClN6O2/c21-18-7-6-17(27(28)29)20(24-18)25-19-8-16-12(9-22-19)2-1-3-15(16)13-10-23-26(11-13)14-4-5-14/h1-3,6-11,14H,4-5H2,(H,22,24,25) | N/D |
| 7f | 1S/C19H15FN6O3/c20-17-5-4-16(26(28)29)19(23-17)24-18-8-15-12(9-21-18)2-1-3-14(15)13-10-22-25(11-13)6-7-27/h1-5,8-11,27H,6-7H2,(H,21,23,24) | N/A^b^ |
| 7g | 1S/C24H15FN4O2/c25-22-11-10-21(29(30)31)24(27-22)28-23-13-20-18(14-26-23)6-3-7-19(20)17-9-8-15-4-1-2-5-16(15)12-17/h1-14H,(H,26,27,28) | N/D |
| 8g | 1S/C24H15ClN4O2/c25-22-11-10-21(29(30)31)24(27-22)28-23-13-20-18(14-26-23)6-3-7-19(20)17-9-8-15-4-1-2-5-16(15)12-17/h1-14H,(H,26,27,28) | N/D |
| 7h | 1S/C25H16FN5O2/c26-23-11-10-22(31(32)33)25(29-23)30-24-13-21-19(15-28-24)3-1-5-20(21)17-8-6-16(7-9-17)18-4-2-12-27-14-18/h1-15H,(H,28,29,30) | N/D |
| 8h | 1S/C25H16ClN5O2/c26-23-11-10-22(31(32)33)25(29-23)30-24-13-21-19(15-28-24)3-1-5-20(21)17-8-6-16(7-9-17)18-4-2-12-27-14-18/h1-15H,(H,28,29,30) | N/D |
| 7i | 1S/C25H16FN5O2/c26-23-9-8-22(31(32)33)25(29-23)30-24-14-21-19(15-28-24)2-1-3-20(21)18-6-4-16(5-7-18)17-10-12-27-13-11-17/h1-15H,(H,28,29,30) | > 1000 |
| 8i | 1S/C25H16ClN5O2/c26-23-9-8-22(31(32)33)25(29-23)30-24-14-21-19(15-28-24)2-1-3-20(21)18-6-4-16(5-7-18)17-10-12-27-13-11-17/h1-15H,(H,28,29,30) | N/D |

^a^ IC_50_ determination with USP7cd (10 nM) was carried out with Ub-AMC (500 nM) in a black non-binding polystyrene 384-well microplate (Greiner Bio One, Frickenhausen, Germany). Different concentrations of compounds were pre-incubated with USP7 for 24 h at 20 °C in a rotating shaker in 25 mM Tris pH 8.0, 150mM NaCl, 5 mM TCEP supplemented with 0.2 mg/mL bovine serum albumin (BSA). Fluorescence was measured after 1 h of the addition of Ub-AMC. The dose response semi-logarithmic curves were fitted using OriginPro2021 and the four-parameter logistic function.

^b^ no evaluable data points were obtained due to intrinsic fluorescence
