# Supplementary material for: Design, Synthesis, and Molecular Evaluation of SNAr‐Reactive N‐(6‐Fluoro‐3‐Nitropyridin‐2‐yl)Isoquinolin‐3‐Amines as Covalent USP7 Inhibitors Reveals an Unconventional Binding Mode
Source: Arch Pharm (Weinheim). 2025 Aug 10;358(8):e70053. doi: 10.1002/ardp.70053 (PMC12336439; doi:10.1002/ardp.70053)
Supplement: Supplementary file 2 — Synthese paper SI. [file ARDP-358-e70053-s001.docx]

**Supporting Information**

**Design, Synthesis and Molecular Evaluation of S_N_Ar-reactive *N*-(6-Fluoro-3-nitropyridin-2‑yl)isoquinolin-3-amine Derivatives as Covalent Inhibitors of the Cysteine Protease USP7 Reveals an Unconventional Binding Mode**

Larissa N. Ernst^1^, Jason Stahlecker^1^, Finn Mier^1^, Ricardo A. M. Serafim^2,3#^, Valentin R. Wydra^3^, Benedikt Masberg^4^, Simon J. Jaag^4^, Cornelius Knappe^4^, Michael Lämmerhofer^4^, Thilo Stehle^5^, Matthias Gehringer^2,3,6‡*^, Frank M. Boeckler^1,7‡*^

1 Department of Pharmacy and Biochemistry, Eberhard Karls Universität Tübingen, Laboratory for Molecular Design & Pharmaceutical Biophysics, Institute of Pharmaceutical Sciences, 72076 Tübingen, Germany

2 Department of Medicinal Chemistry, Eberhard Karls Universität Tübingen, Faculty of Medicine, Institute for Biomedical Engineering, 72076 Tübingen, Germany

3 Department of Pharmacy and Biochemistry, Eberhard Karls Universität, Pharmaceutical Chemistry, Institute of Pharmaceutical Sciences, 72076 Tübingen, Germany

4 Department of Pharmacy and Biochemistry, Eberhard Karls Universität, Pharmaceutical (Bio‑)Analysis, Institute of Pharmaceutical Sciences, 72076 Tübingen, Germany

5 Interfaculty Institute of Biochemistry, Eberhard Karls Universität Tübingen, 72076 Tübingen, Germany

6 Cluster of Excellence iFIT (EXC 2180) ‘Image-Guided & Functionally Instructed Tumor Therapies’, Eberhard Karls Universität Tübingen, 72076 Tübingen, Germany

7 Interfaculty Institute for Biomedical Informatics (IBMI), Eberhard Karls Universität Tübingen, 72076 Tübingen, Germany

# present address: Department of Organic and Pharmaceutical Chemistry, School of Engineering, Institut Químic de Sarrià (IQS), Universitat Ramon Llull (URL), 08017 Barcelona, Spain

‡ Shared last author

*Correspondence:

Prof. Dr. Frank M. Boeckler, Department of Pharmacy and Biochemistry, Eberhard Karls Universität Tübingen, Laboratory for Molecular Design & Pharmaceutical Biophysics, Institute of Pharmaceutical Sciences, 72076 Tübingen, Germany

Auf der Morgenstelle 8 (Haus B)

D – 72076 Tübingen

Tel +49 7071 29 74567

Email frank.boeckler@uni-tuebingen.de

Prof. Dr. Matthias Gehringer, Department of Medicinal Chemistry, Eberhard Karls Universität Tübingen, Faculty of Medicine, Institute for Biomedical Engineering, 72076 Tübingen, Germany

Auf der Morgenstelle 8 (Haus B)

D – 72076 Tübingen

Tel +49 7071 29 74582

Email matthias.gehringer@uni-tuebingen.de

**Content**

[1. NMR Spectra, HRMS and Purity Data 3](#_Toc195044955)

[2. Reactivity measurements with Glutathione (GSH) 49](#_Toc195044956)

[3. X-ray Crystallography 52](#_Toc195044957)

[4. Protein Sequences 55](#_Toc195044958)

[5. Intact Protein Mass Spectrometry 55](#_Toc195044959)

# NMR Spectra, HRMS and Purity Data

**7a**

**
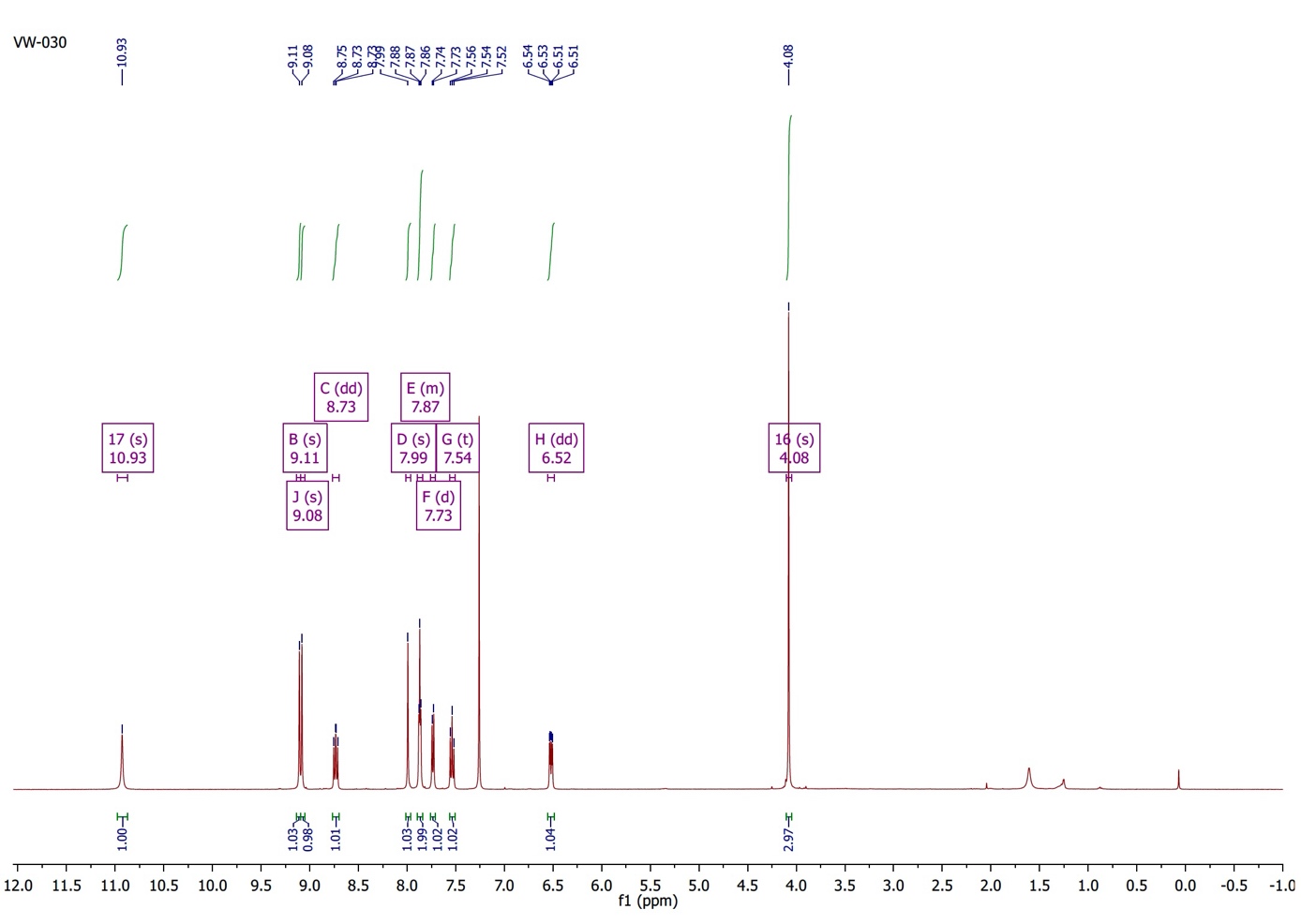
**

**Figure S1.1:** ^1^H NMR (400 MHz, CDCl_3_) **7a**.


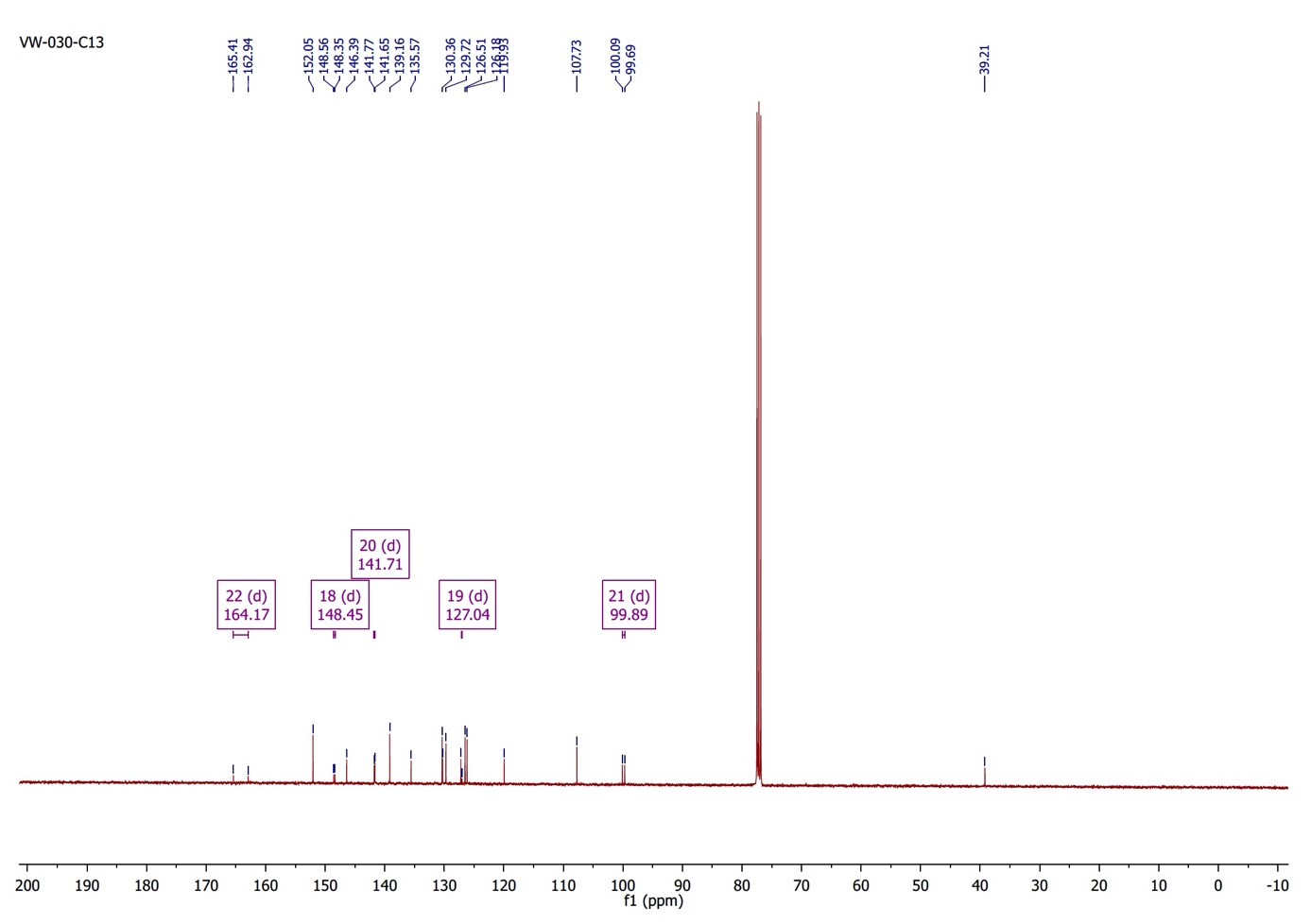


**Figure S1.2:** ^13^C NMR (101 MHz, CDCl3) **7a**.

**
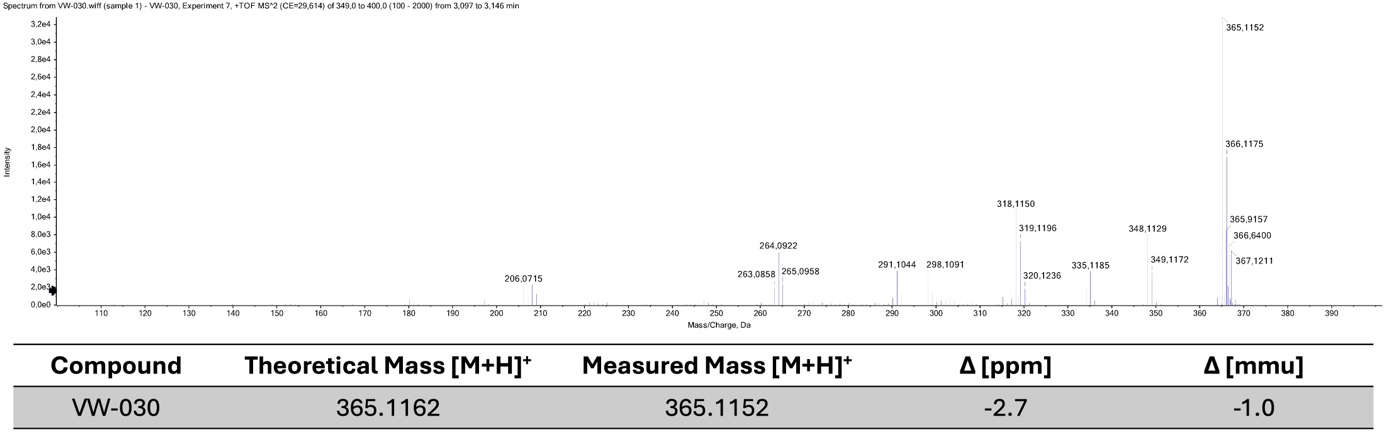
**

**Figure S1.3:** SWATH-MS results of **7a**.


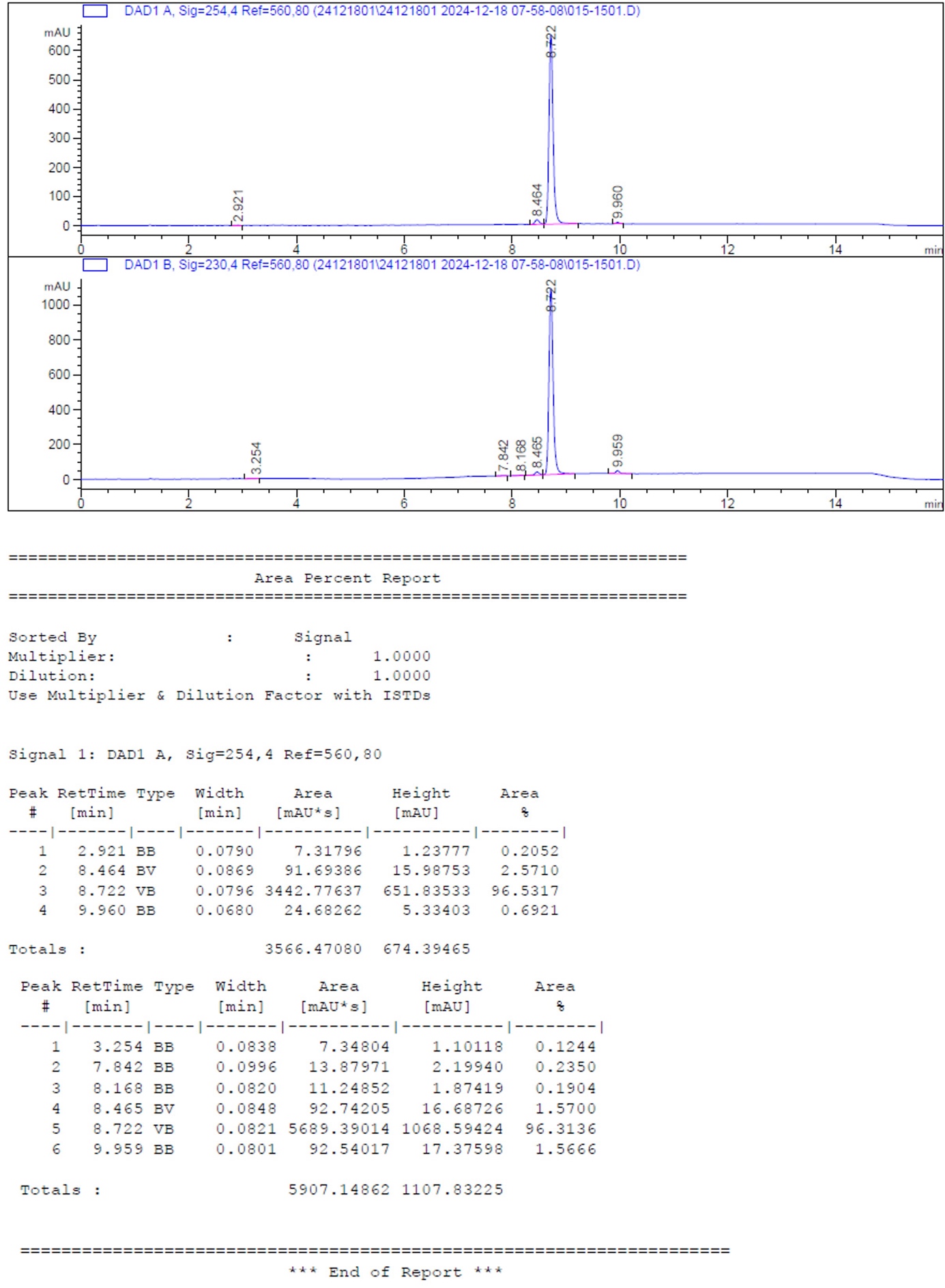


**Figure S1.4:** HPLC traces of compound **7a**.

**8a**

Compound **8a** was the same compound as Wydra et al. published (V Wydra, S Gerstenecker, D Schollmeyer, S Andreev, T Dimitrov, RA Massarico Serafim, S Laufer, M Gehringer. *Molbank*. 2021;2021(1):M1181.)

**7b**

**Figure S1.5:** ^1^H NMR (400 MHz, CDCl_3_) **7b**.

**Figure S1.6:** ^13^C NMR (101 MHz, CDCl3) **7b**.


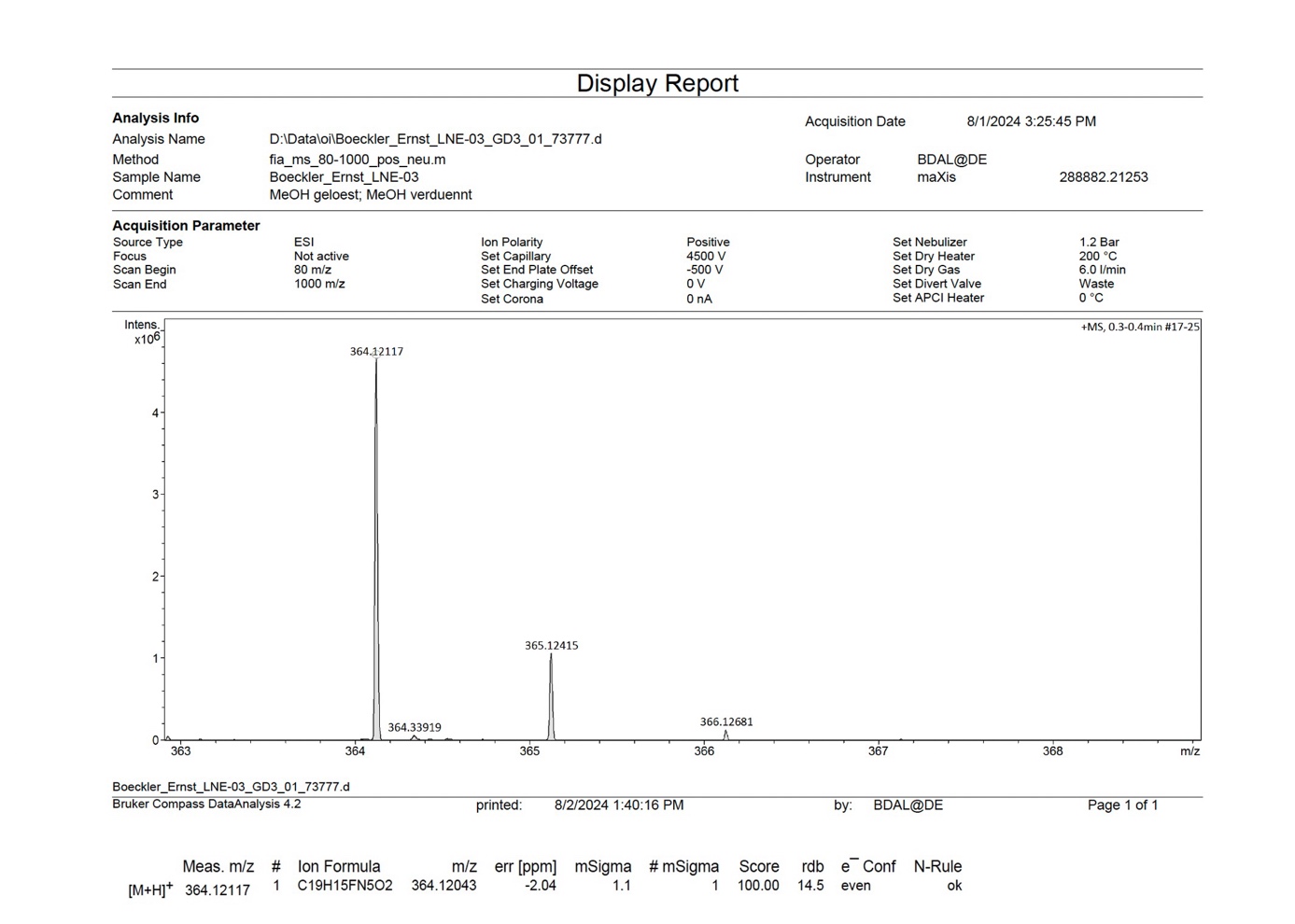


**Figure S1.7:** HRMS ESI-TOF results of **7b**.


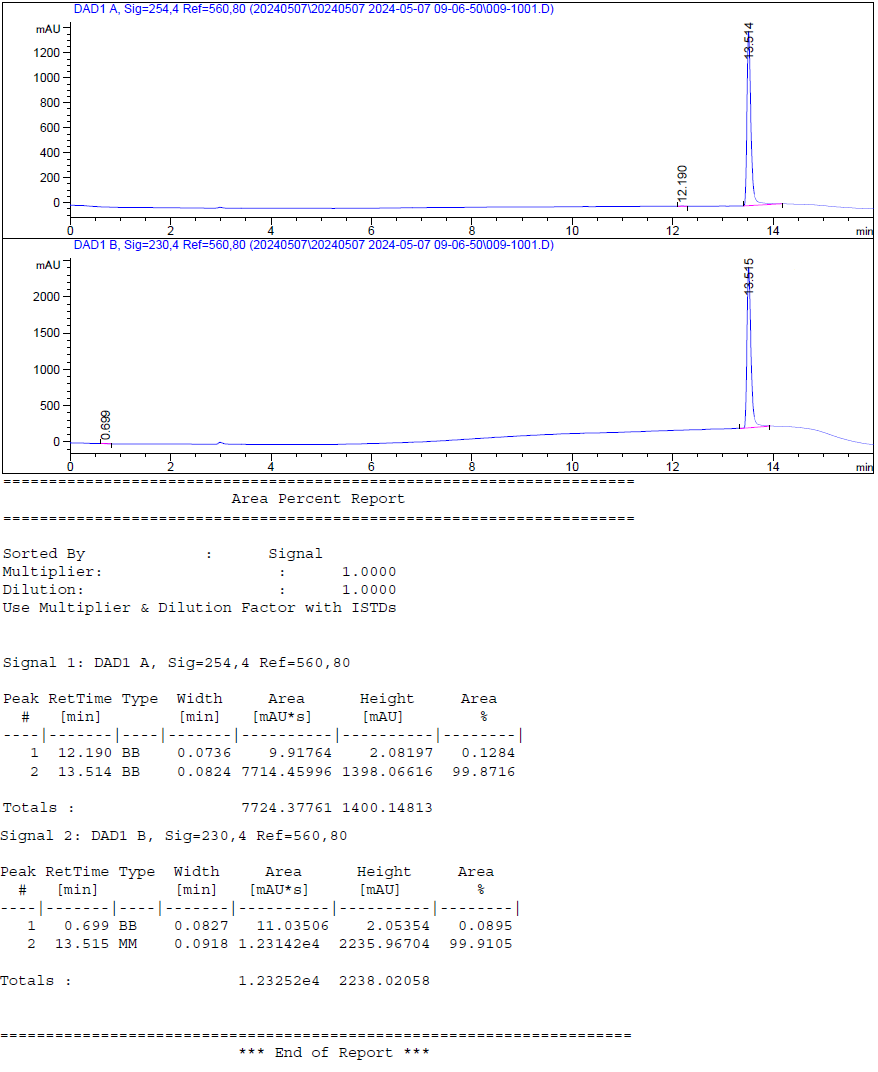


**Figure S1.8:** HPLC traces of compound **7b**.

**8b**

**Figure S1.9:** ^1^H NMR (400 MHz, CDCl_3_) **8b**.

**Figure S1.10:** ^13^C NMR (101 MHz, CDCl3) **8b**.


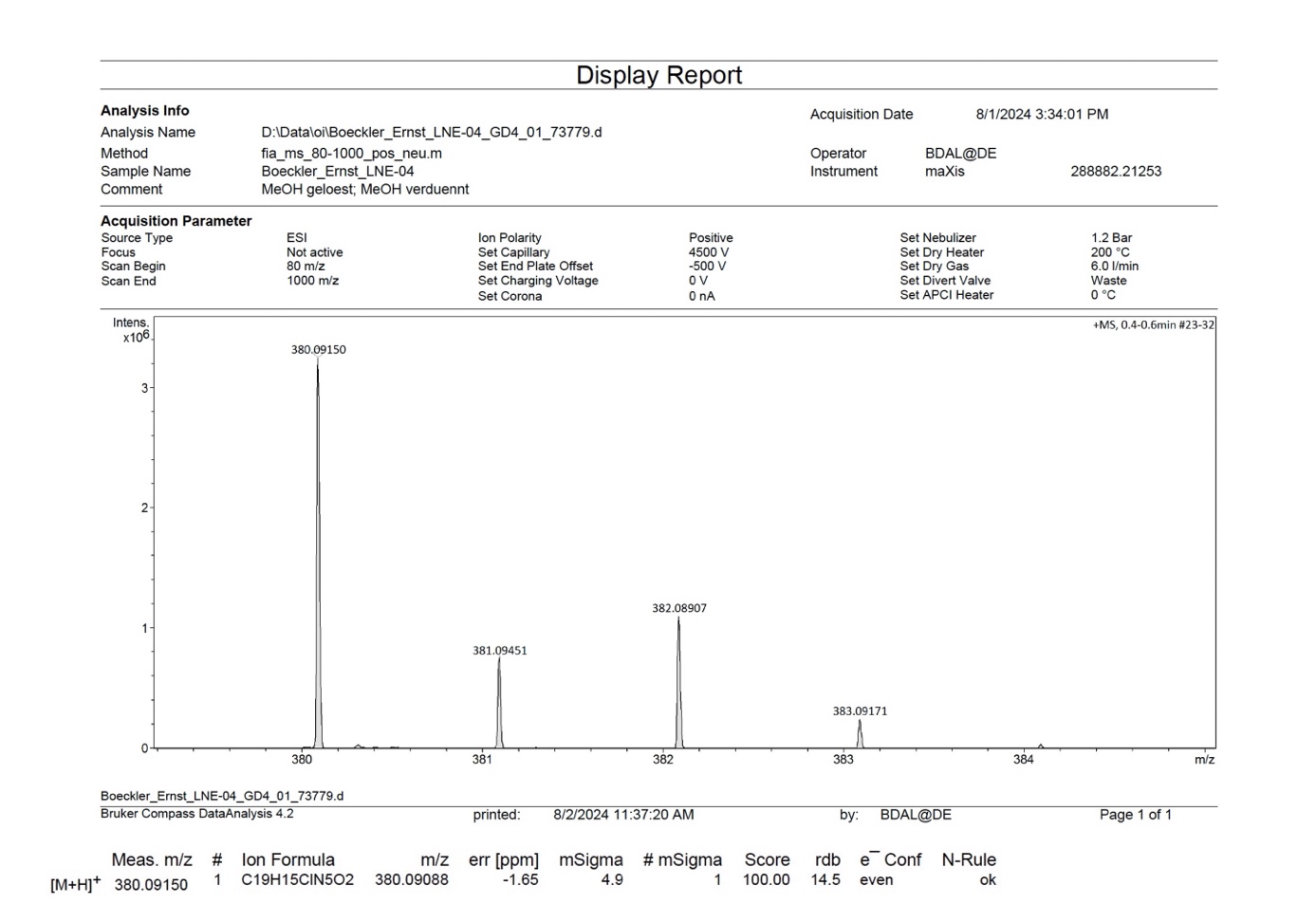


**Figure S1.11:** HRMS ESI-TOF results of **8b**.


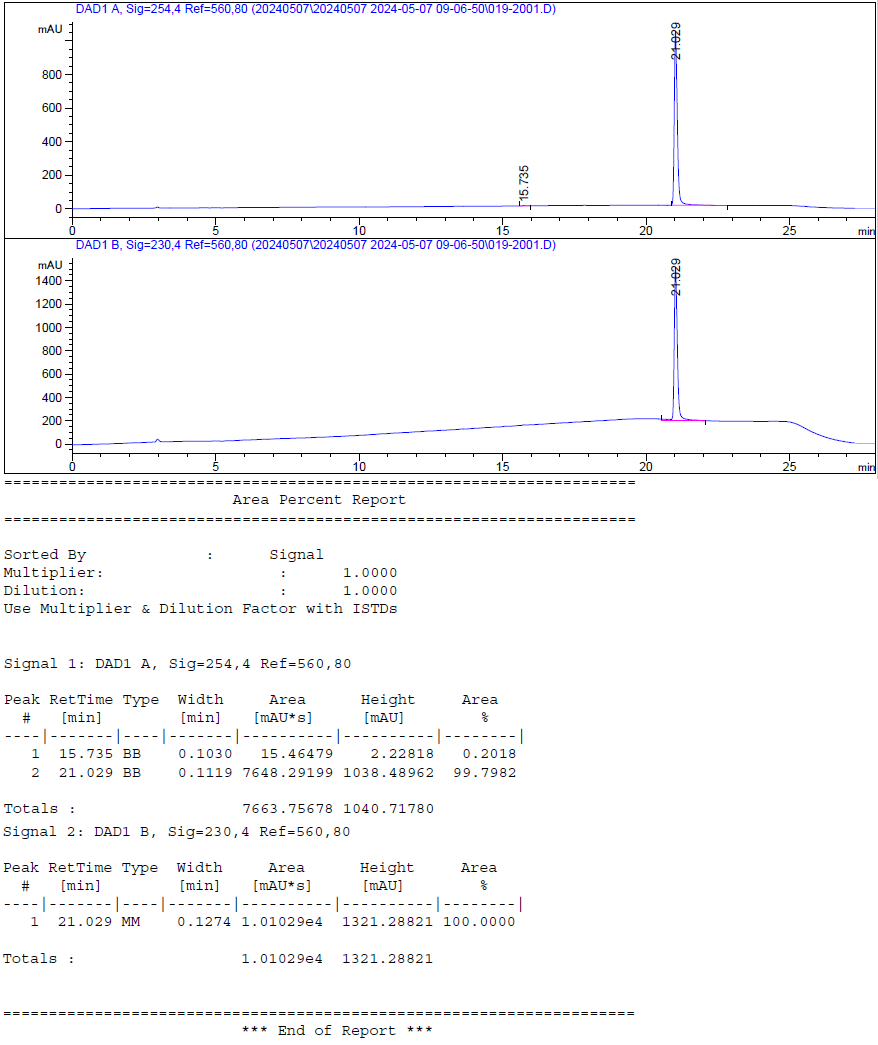


**Figure S1.12:** HPLC traces of compound **8b**.

**7c**

**Figure S1.13:** ^1^H NMR (400 MHz, CDCl_3_) **7c**.

**Figure S1.14:** ^13^C NMR (101 MHz, CDCl3) **7c**.


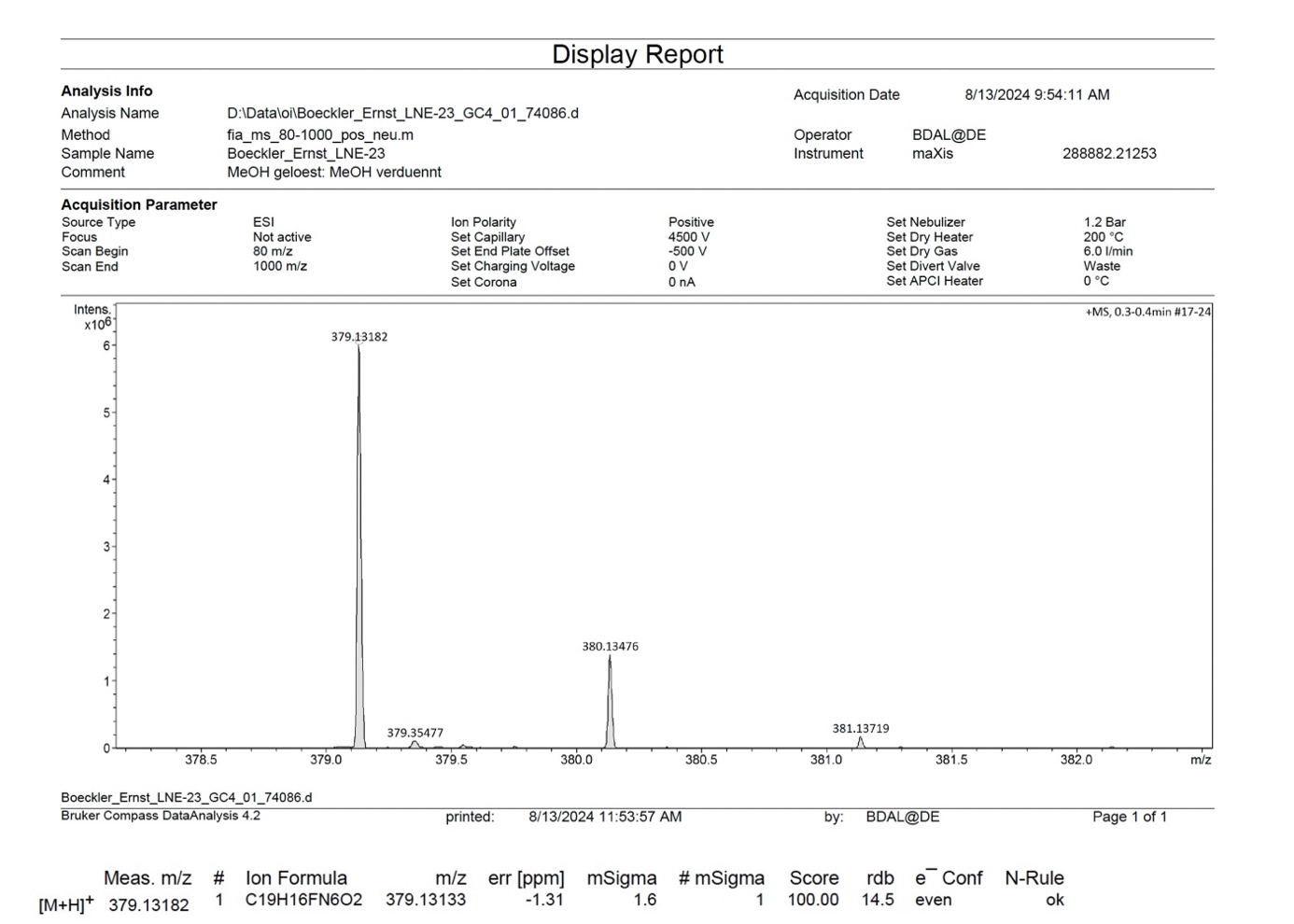


**Figure S1.15:** HRMS ESI-TOF results of **7c**.


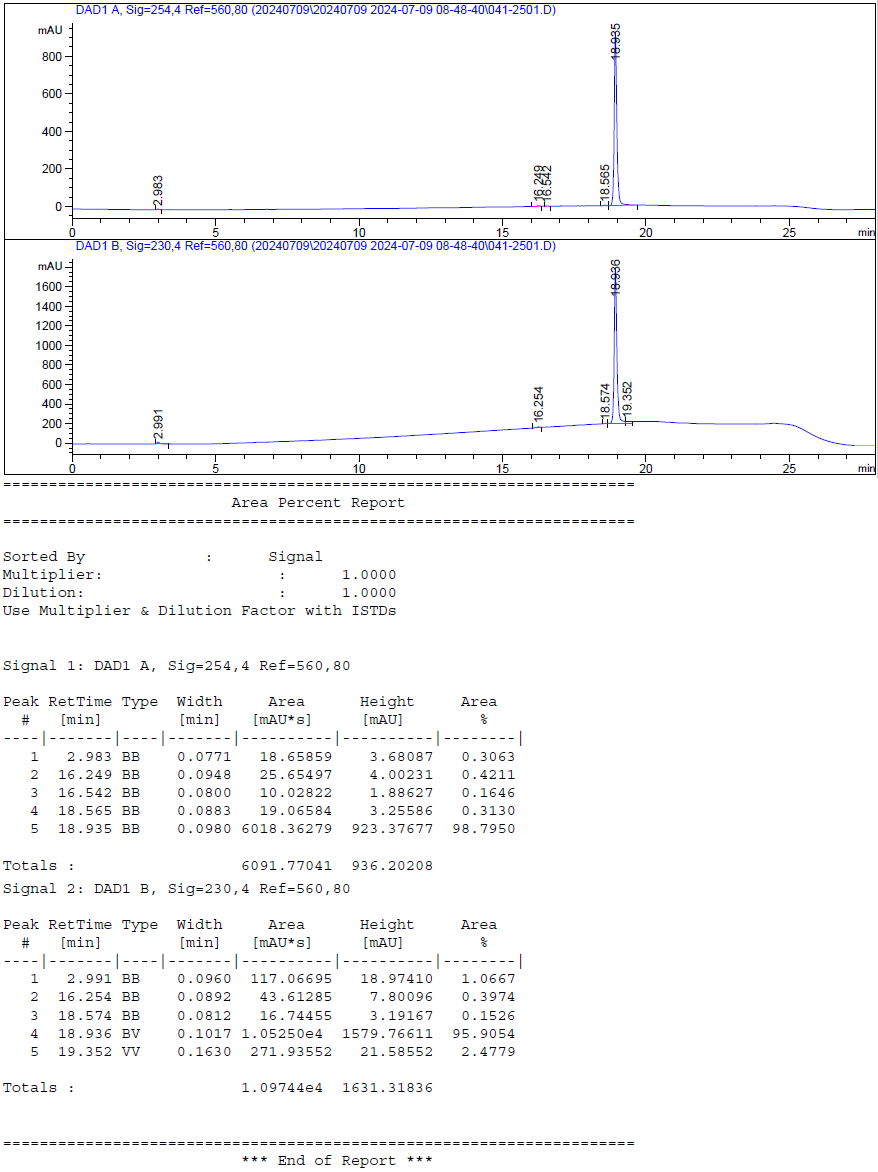


**Figure S1.16:** HPLC traces of compound **7c.**

**8c**

**Figure S1.17:** ^1^H NMR (400 MHz, CDCl_3_) **8c**.

**Figure S1.18:** ^13^C NMR (101 MHz, CDCl3) **8c.**


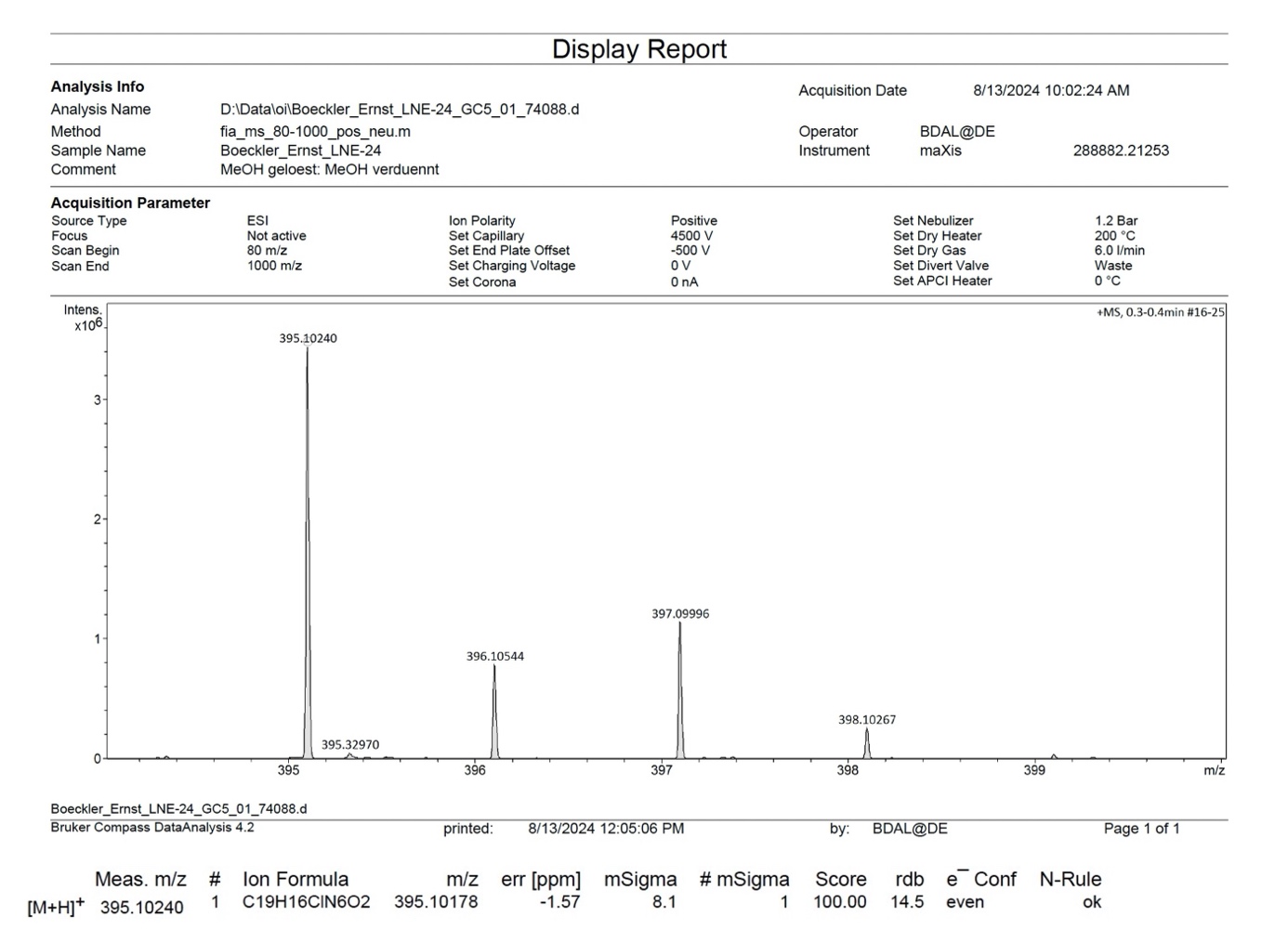


**Figure S1.19:** HRMS ESI-TOF results of **8c**.


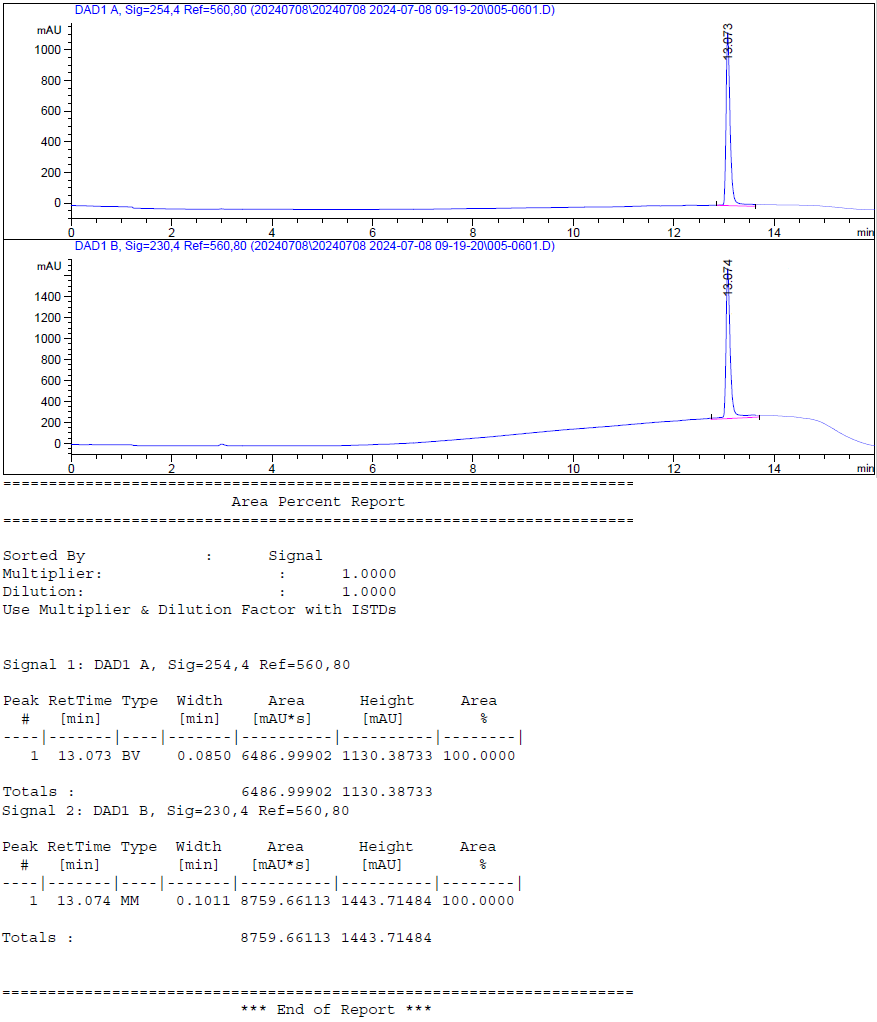


**Figure S1.20:** HPLC traces of compound **8c**.

**7d**

**Figure S1.21:**^1^H NMR (400 MHz, CDCl_3_) **7d**.

**Figure S1.22:**^13^C NMR (101 MHz, CDCl3) **7d**.


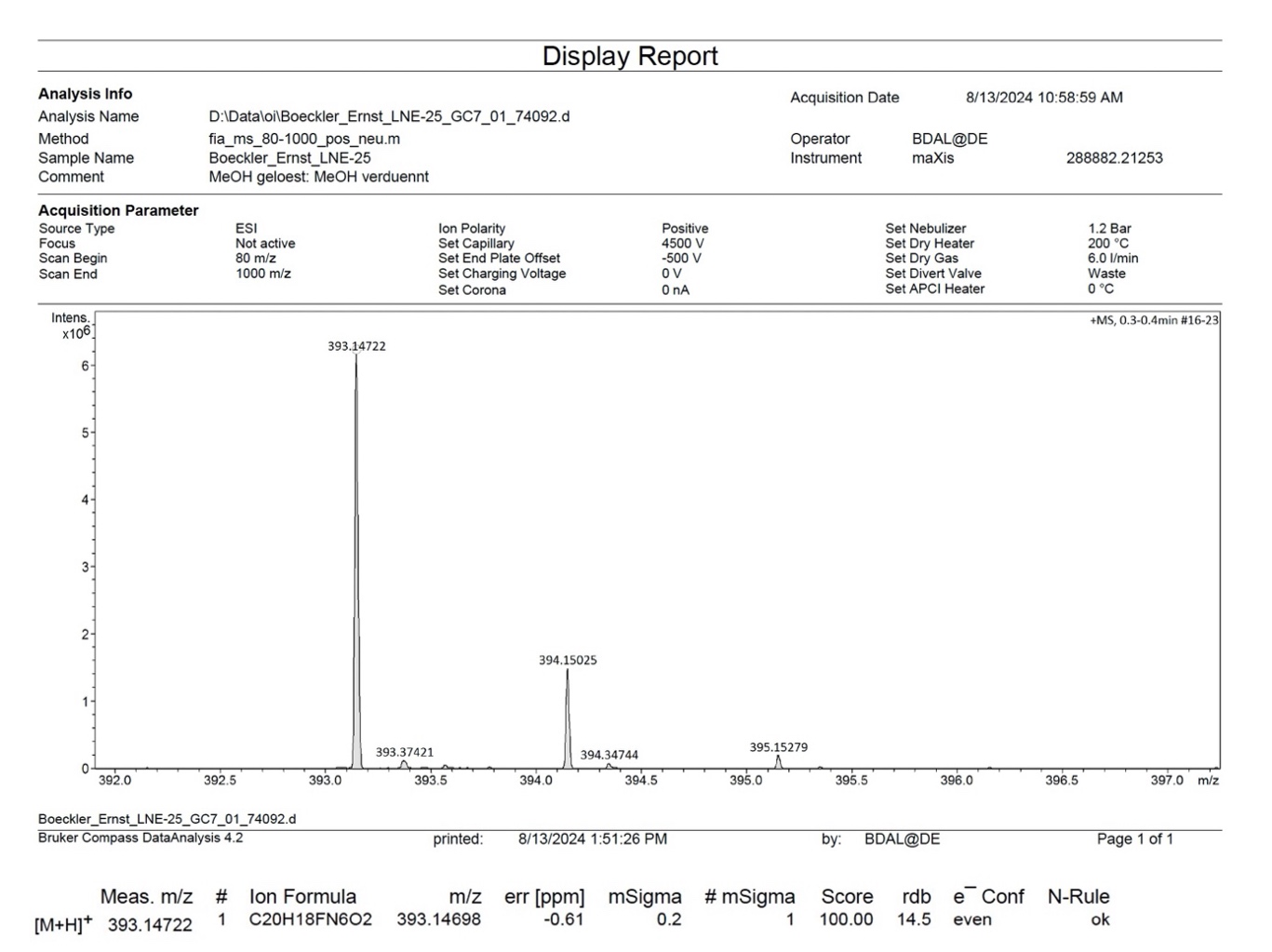


**Figure S1.23:** HRMS ESI-TOF results of **7d**.


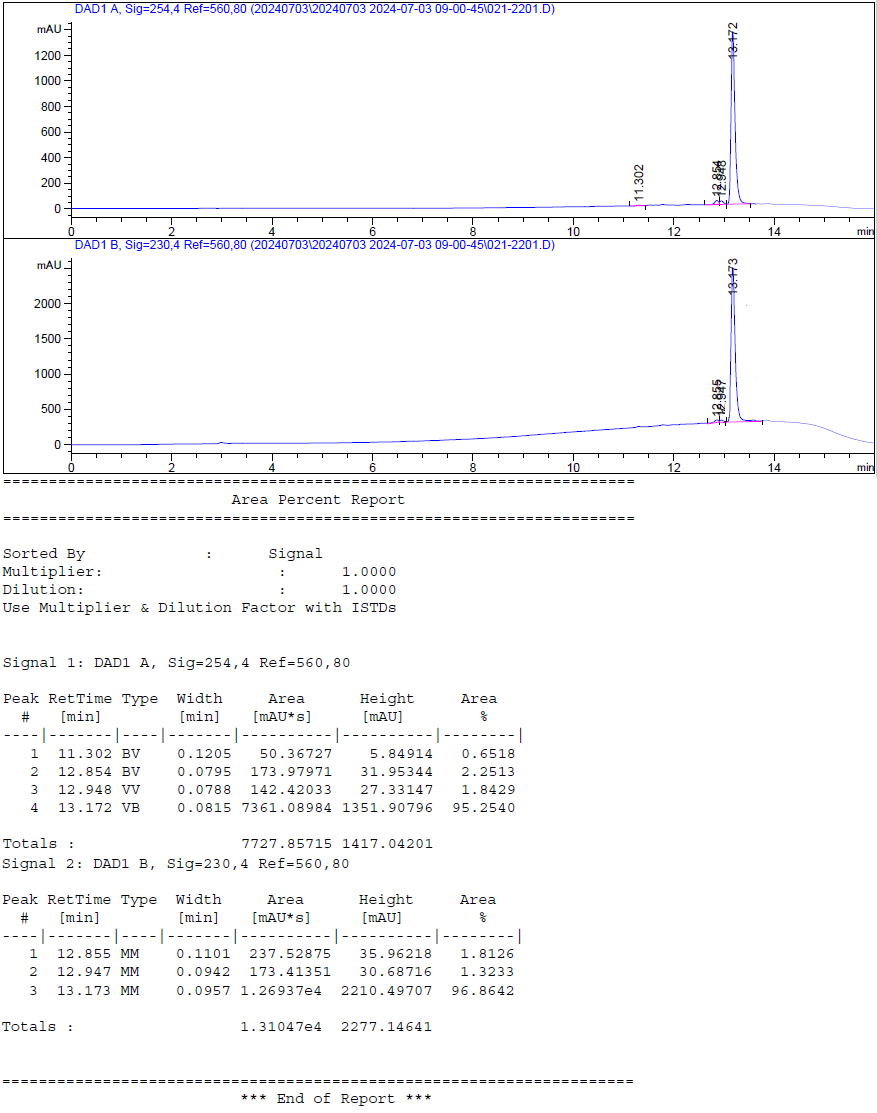


**Figure S1.24:**HPLC traces of compound **7d**.

**8d**

**Figure S1.25:**^1^H NMR (400 MHz, CDCl_3_) **8d**.

**Figure S1.26:**^13^C NMR (101 MHz, CDCl3) **8d**.


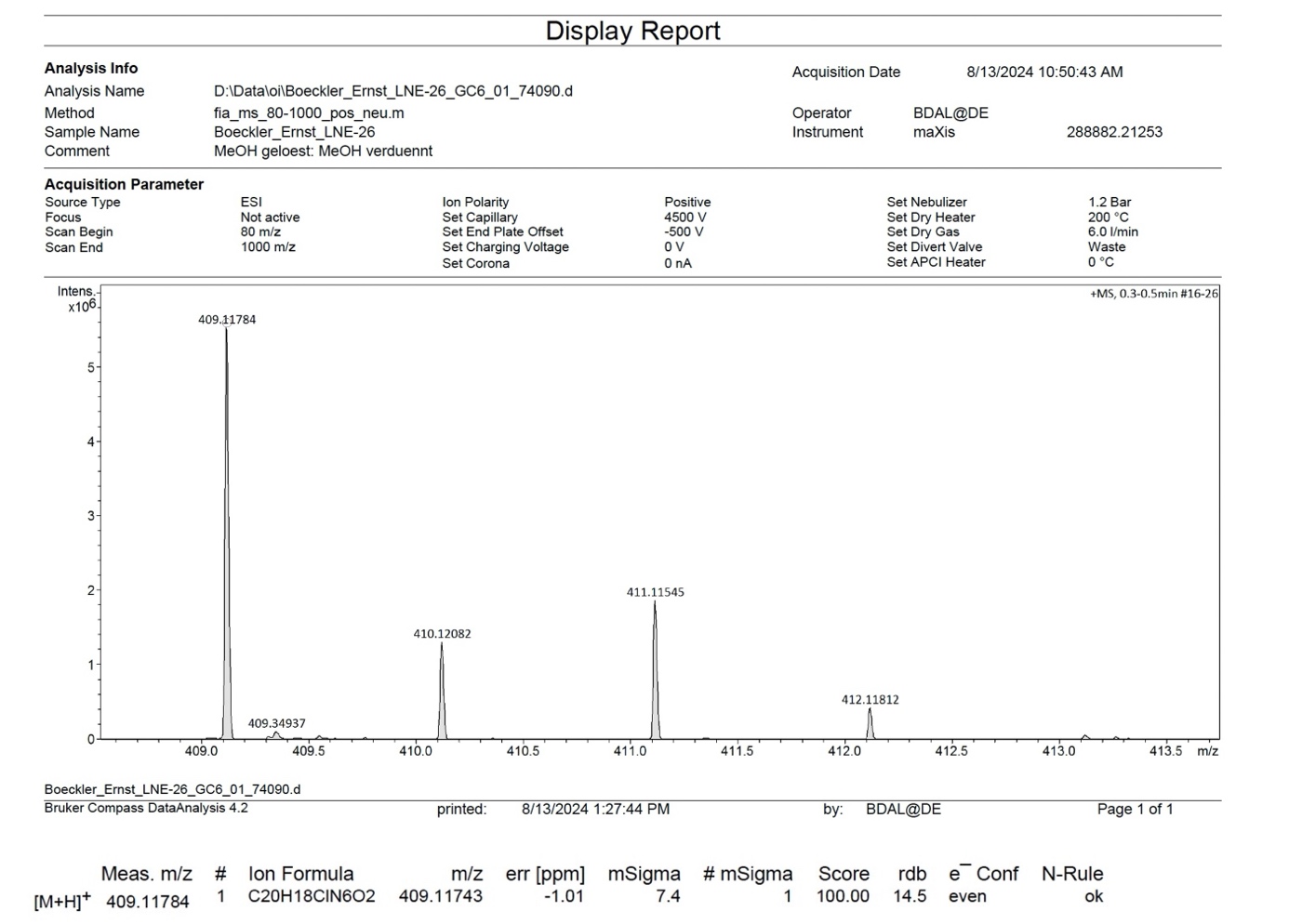


**Figure S1.27:** HRMS ESI-TOF results of **8d**.


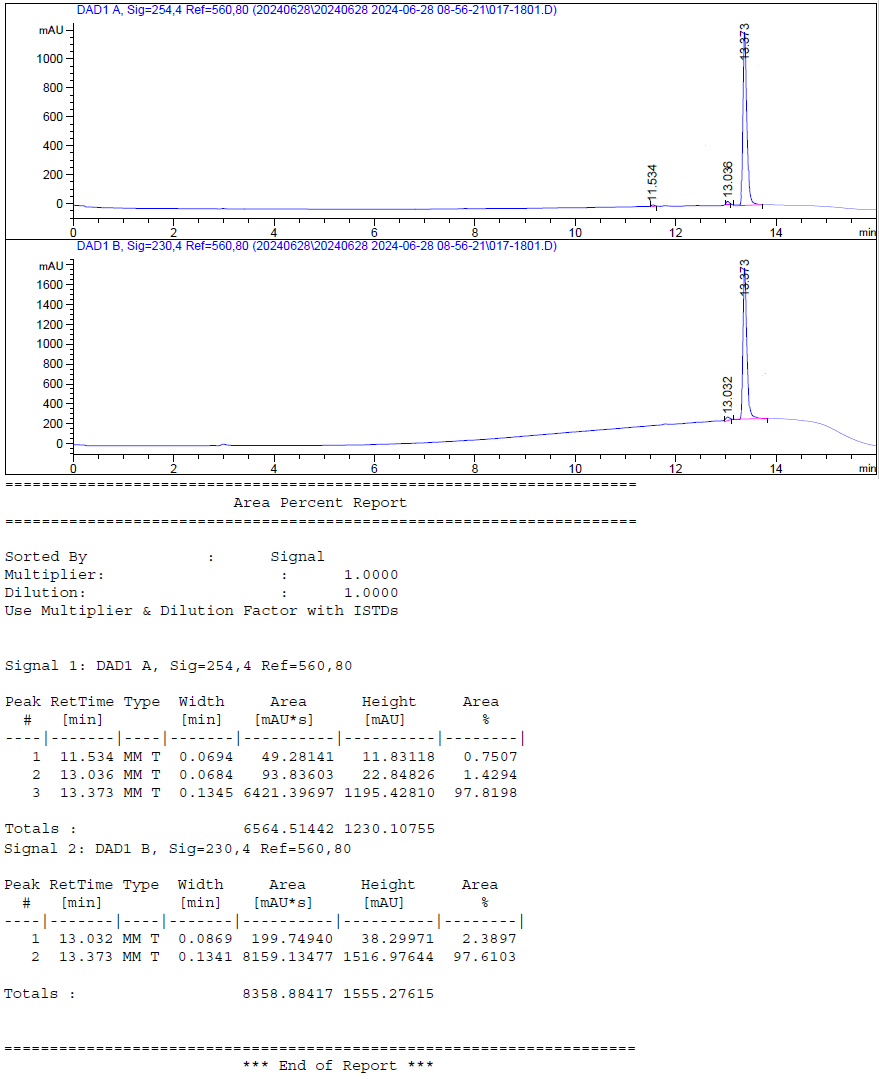


**Figure S1.28:** HPLC traces of compound **8d**.

**7e**

**Figure S1.29:** ^1^H NMR (400 MHz, CDCl_3_) **7e**.

**Figure S1.30:** ^13^C NMR (101 MHz, CDCl3) **7e**.


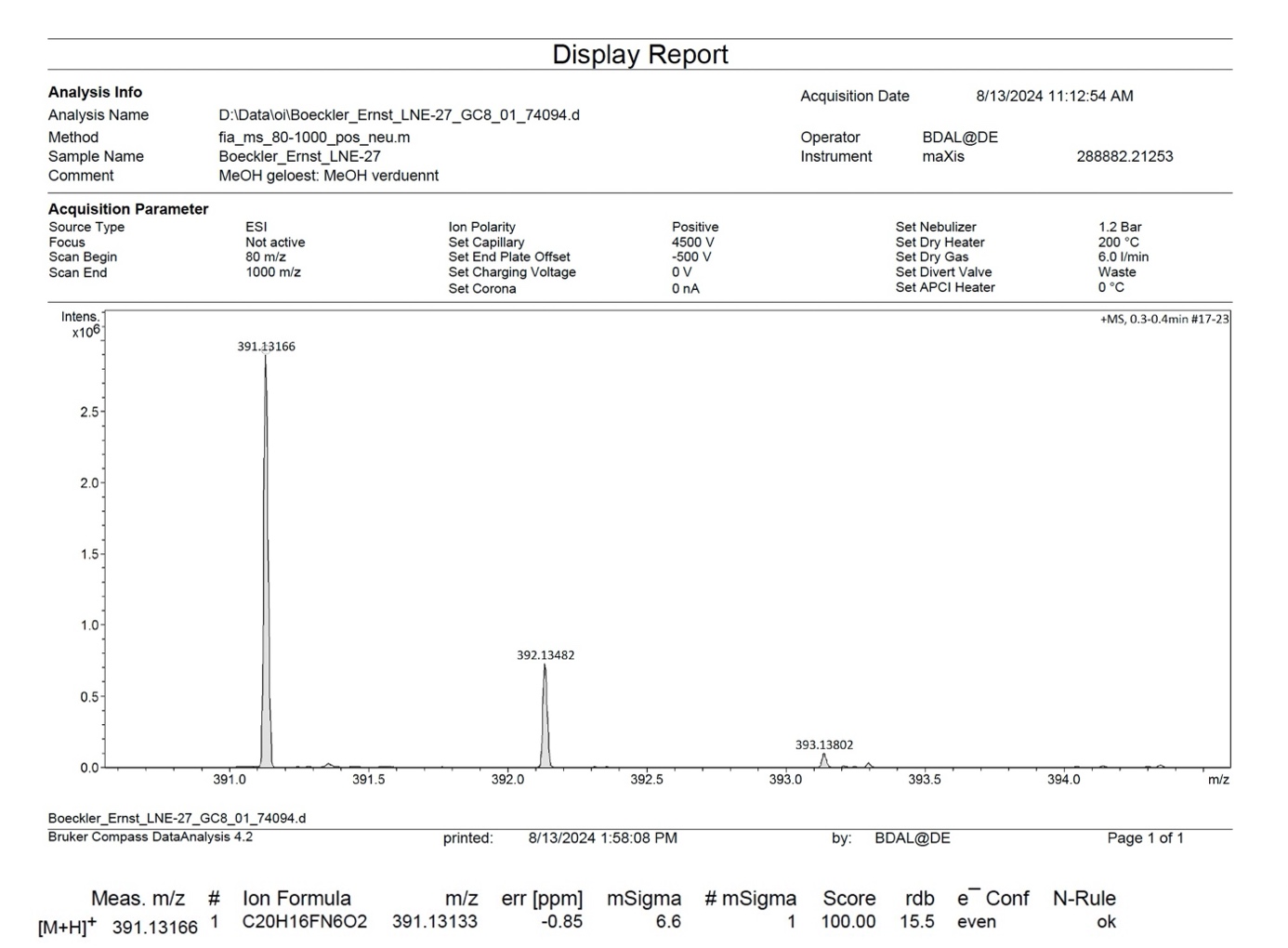


**Figure S1.31:** HRMS ESI-TOF results of **7e**.


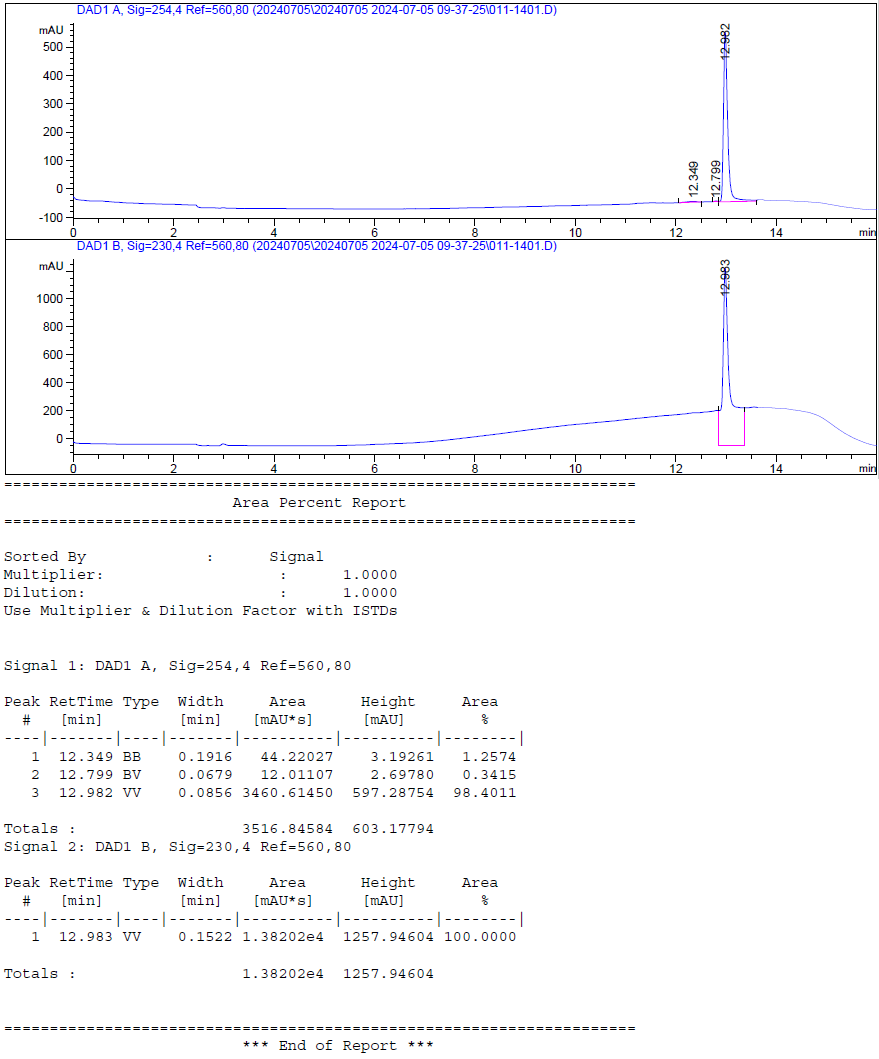


**Figure S1.32:** HPLC traces of compound **7e**.

**8e**

**Figure S1.33:** ^1^H NMR (400 MHz, CDCl_3_) **8e**.

**Figure S1.34:** ^13^C NMR (101 MHz, CDCl3) **8e**.


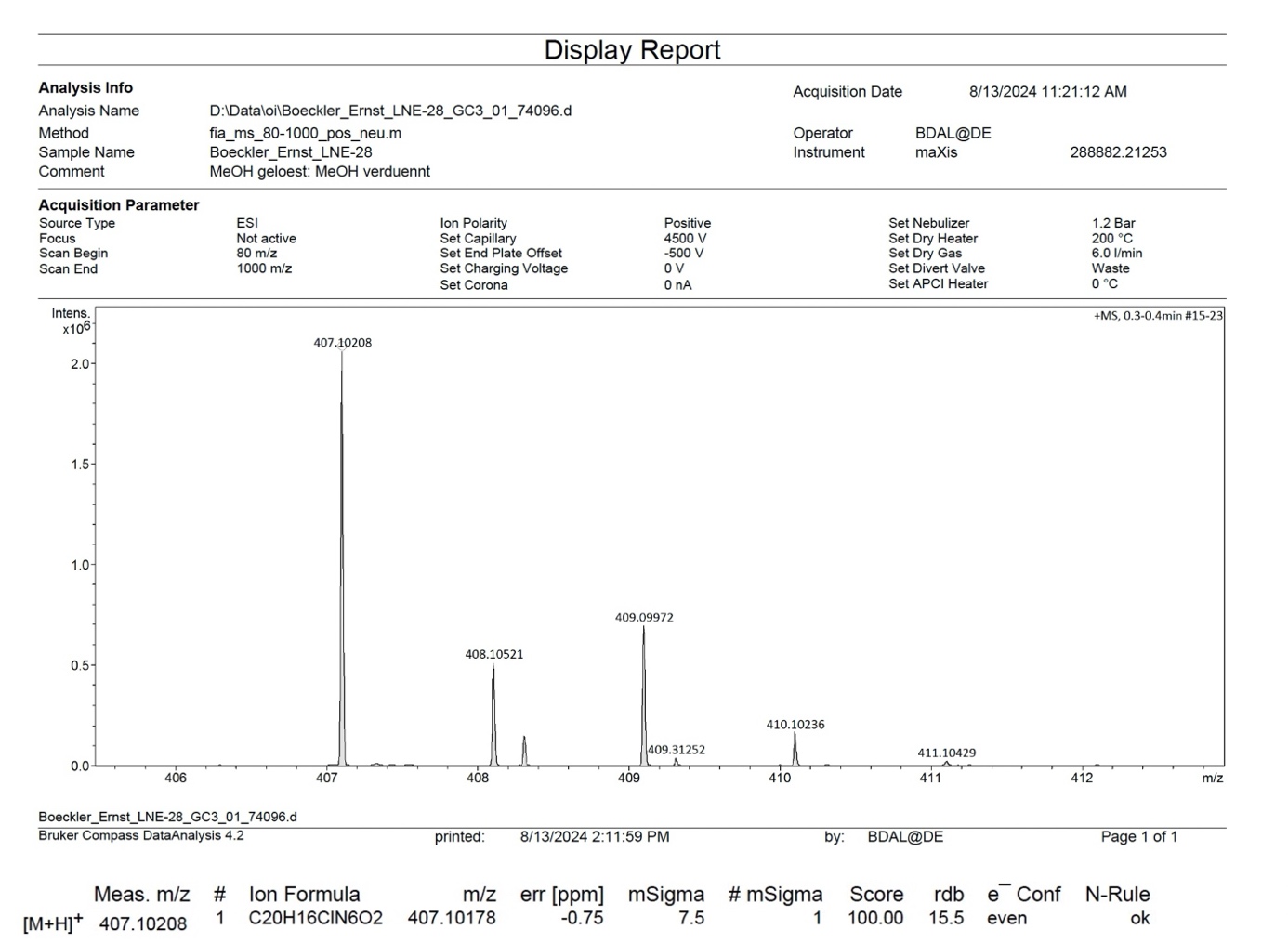


**Figure S1.35:** HRMS ESI-TOF results of **8e**.


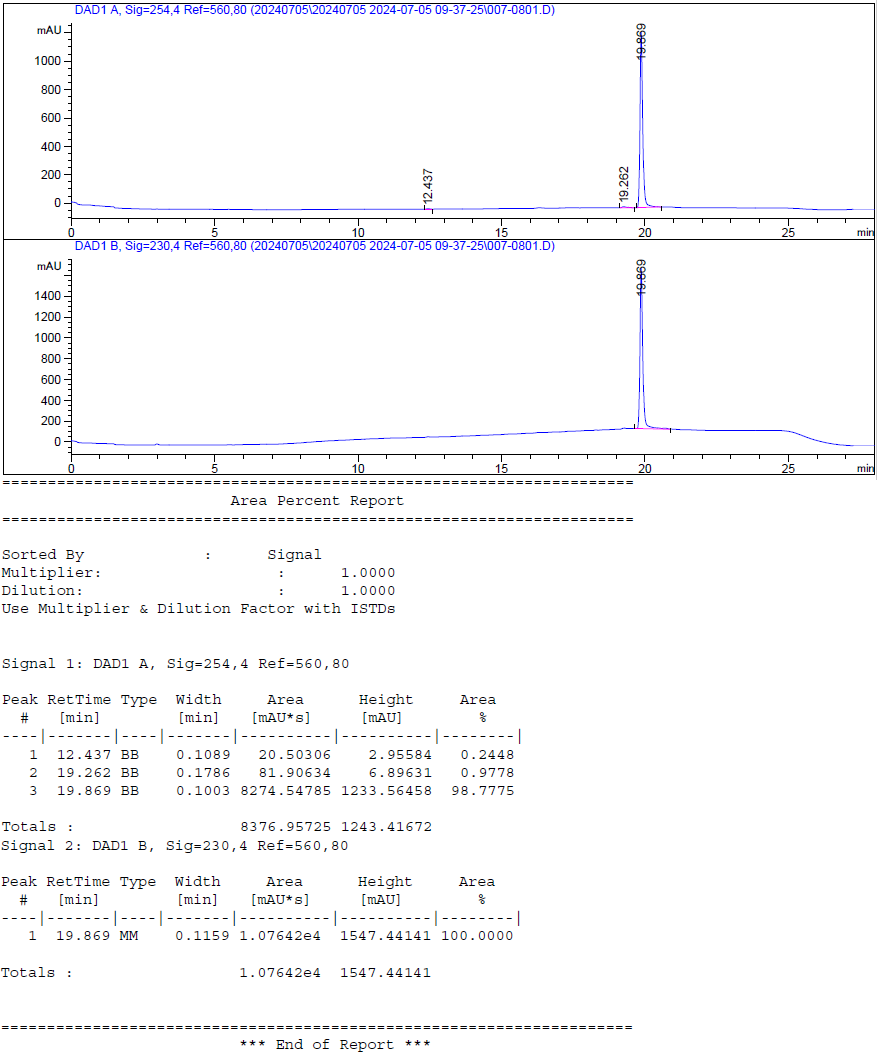


**Figure S1.36:** HPLC traces of compound **8e**.

**7f**

**Figure S1.37:** ^1^H NMR (400 MHz, CDCl_3_) **7f**.

**Figure S1.38:** ^13^C NMR (151 MHz, CDCl3) **7f**.


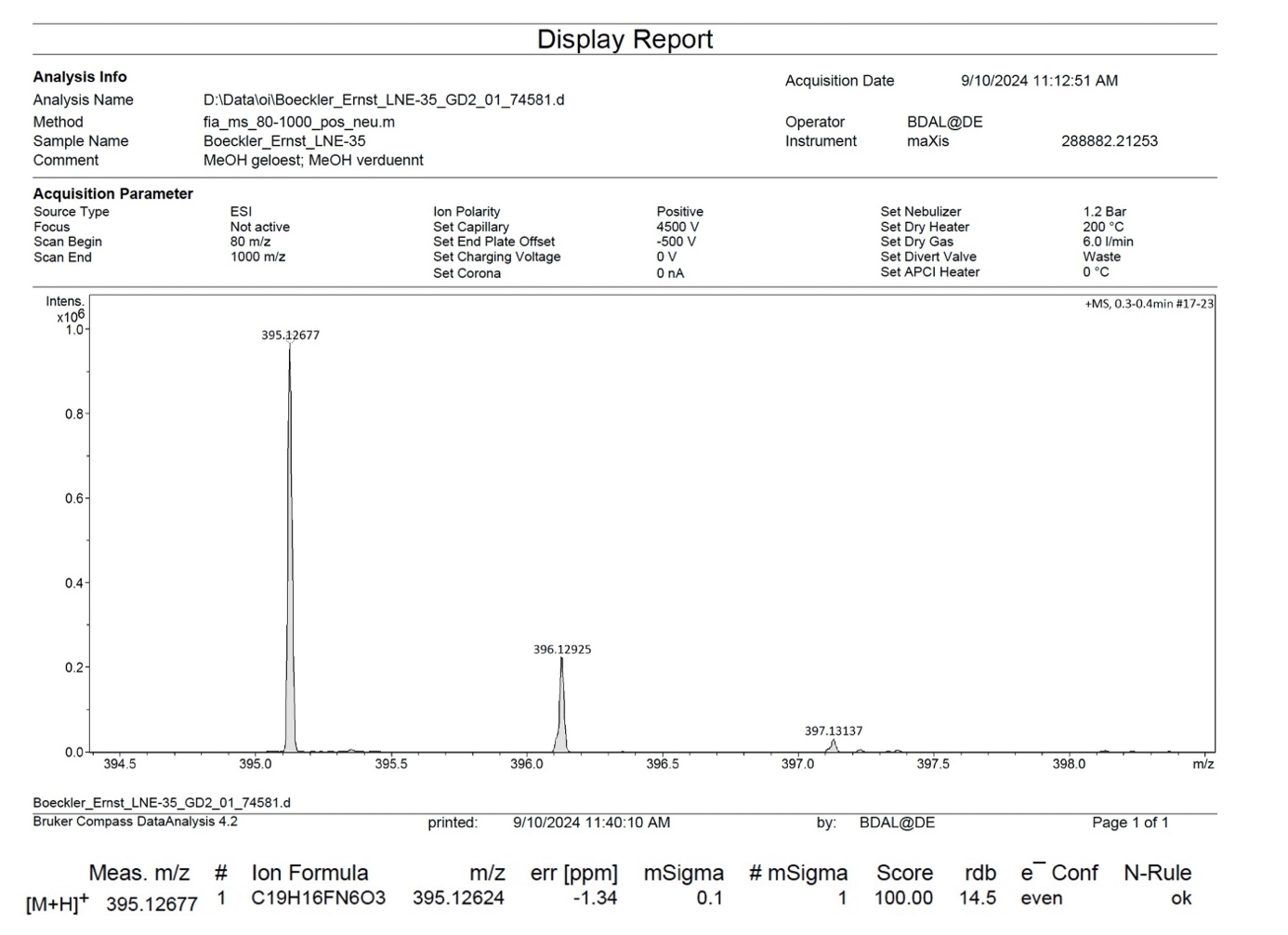


**Figure S1.39:** HRMS ESI-TOF results of **7f**.


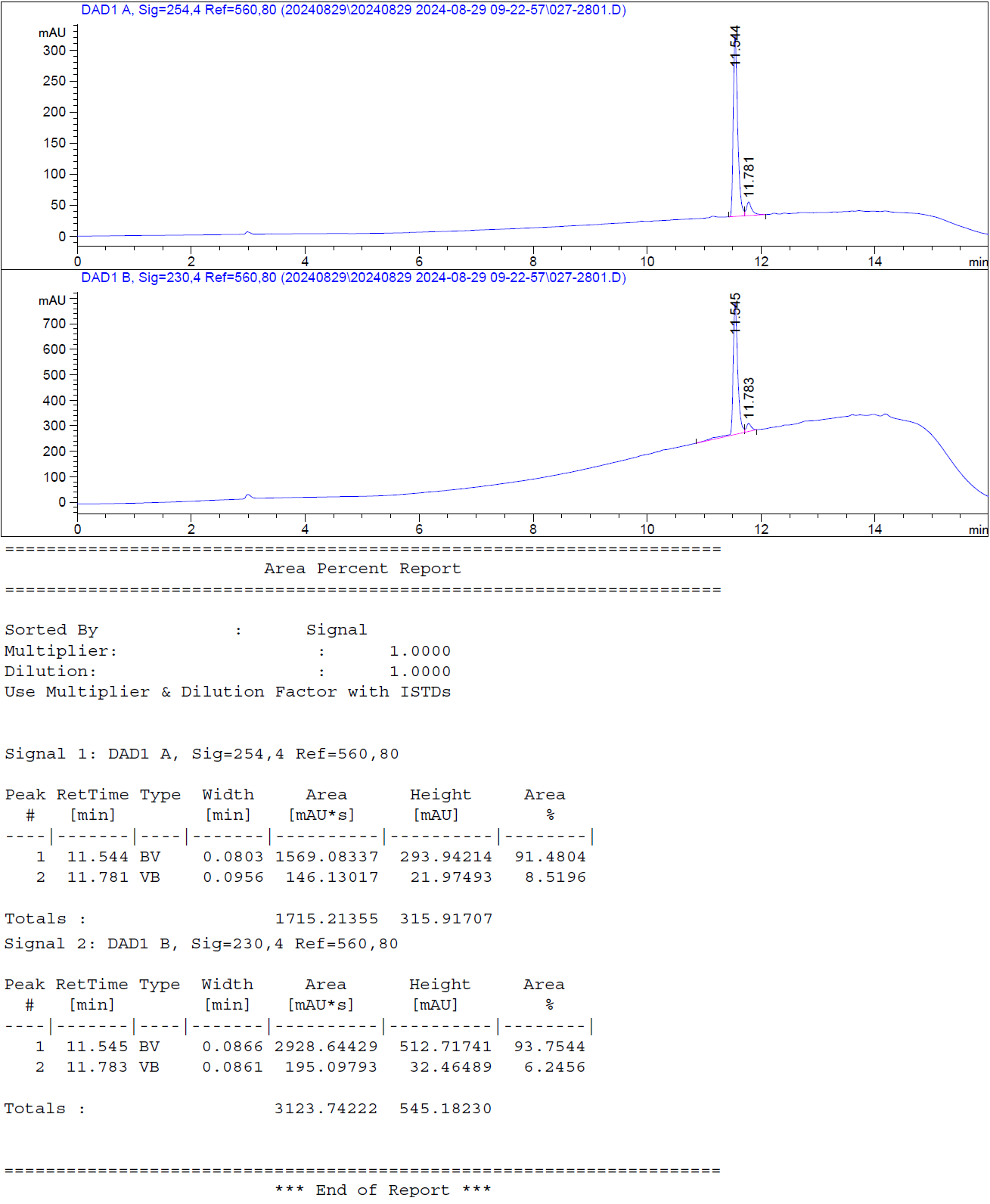


**Figure S1.40:** HPLC traces of compound **7f**.

**7g**

**Figure S1.41:** ^1^H NMR (400 MHz, CDCl_3_) **7g**.

**Figure S1.42:** ^13^C NMR (101 MHz, CDCl3) **7g**.


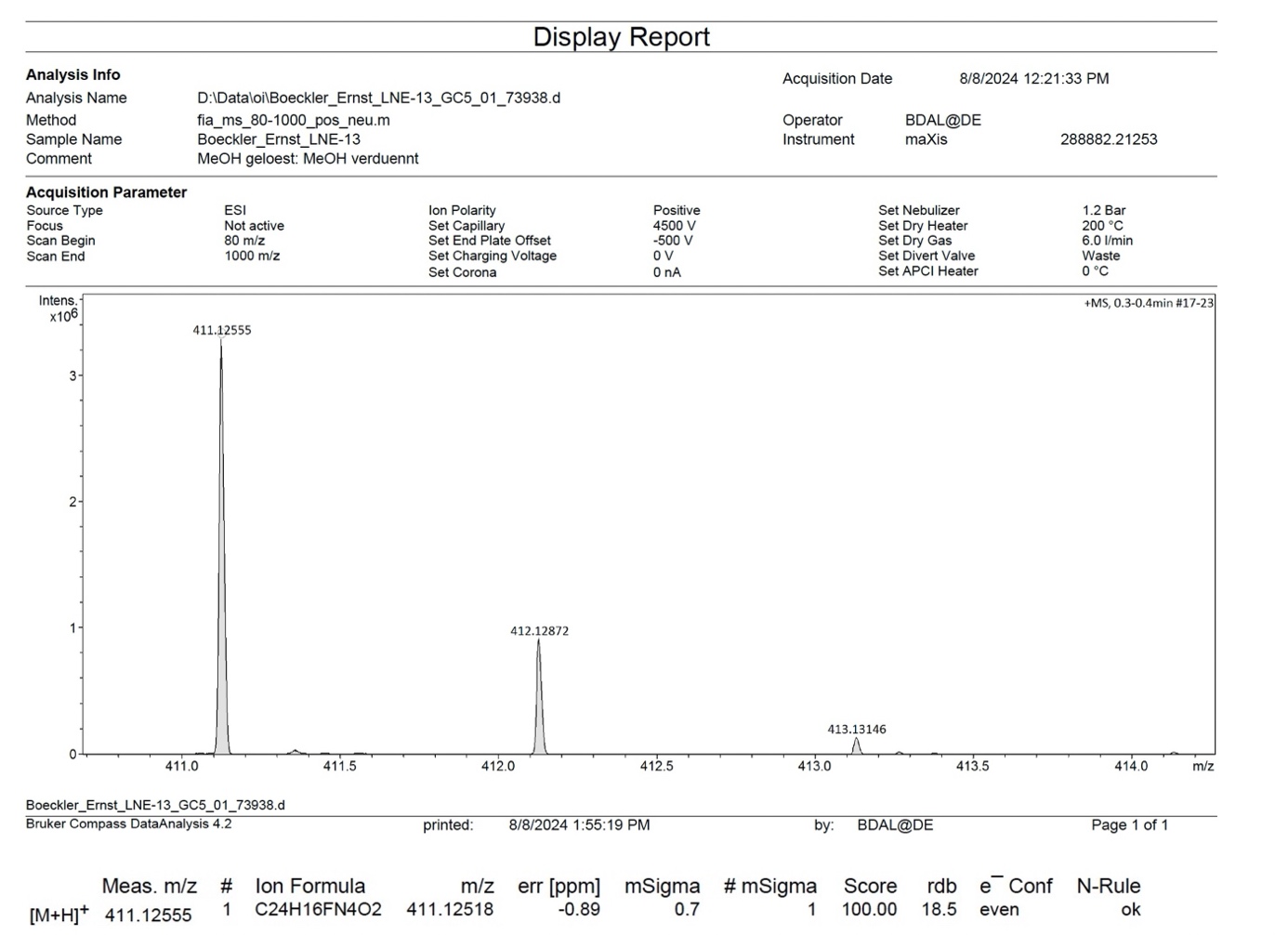


**Figure S1.43:** HRMS ESI-TOF results of **7g**.


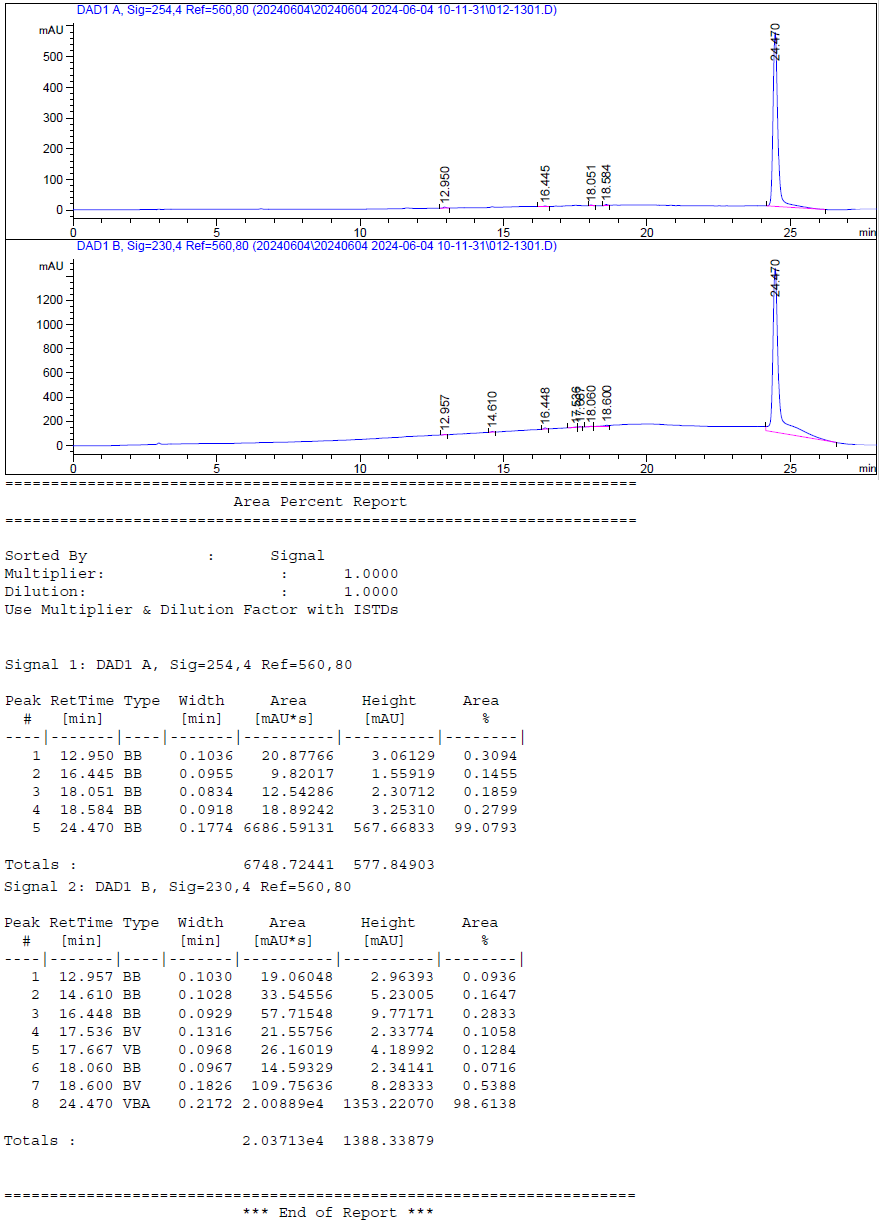


**Figure S1.44:** HPLC traces of compound **7g**.

**8g**

**Figure S1.45:** ^1^H NMR (400 MHz, CDCl_3_) **8g**.

**Figure S1.46:** ^13^C NMR (101 MHz, CDCl3) **8g**.


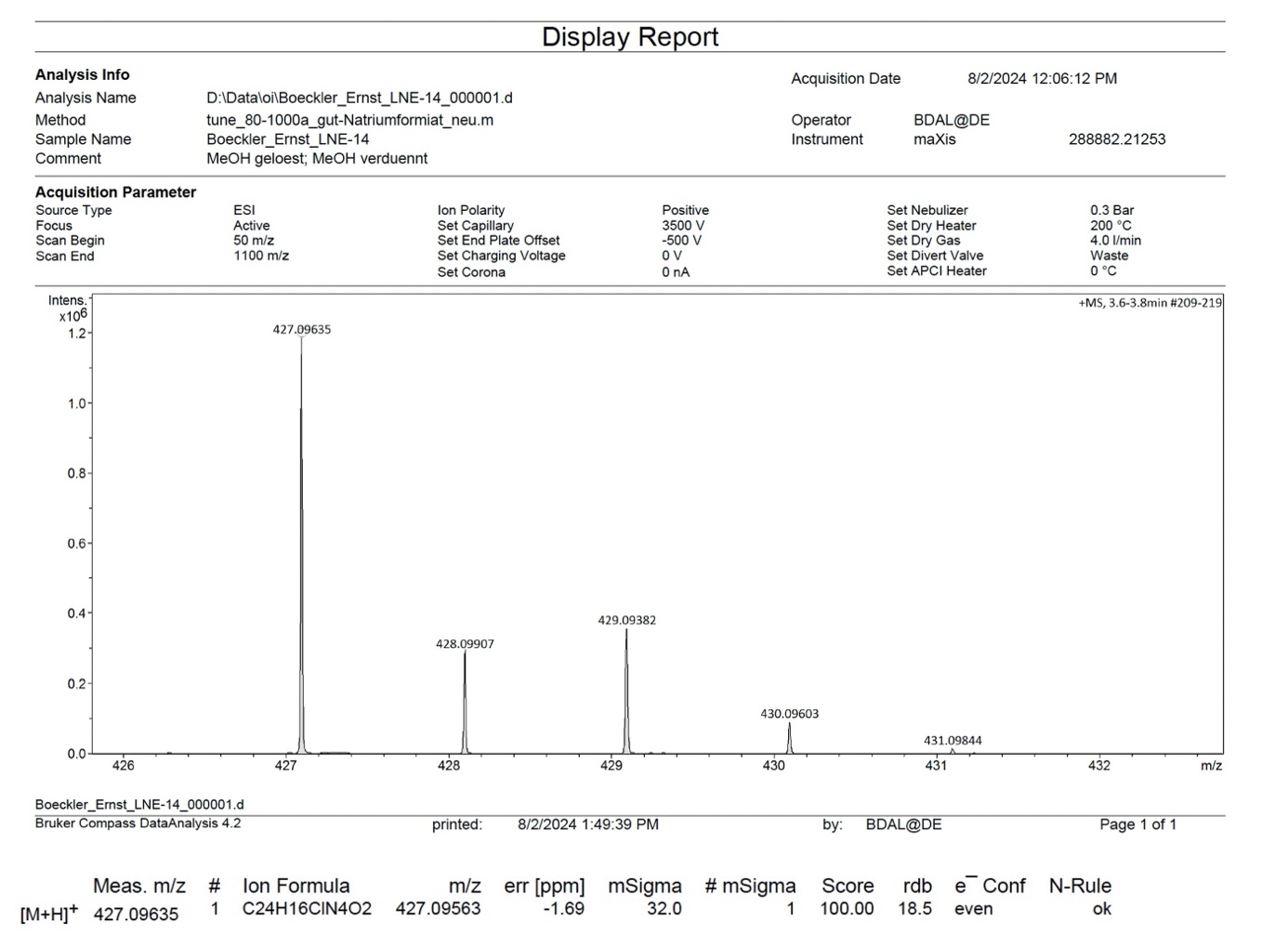


**Figure S1.47:** HRMS ESI-TOF results of **8g**.


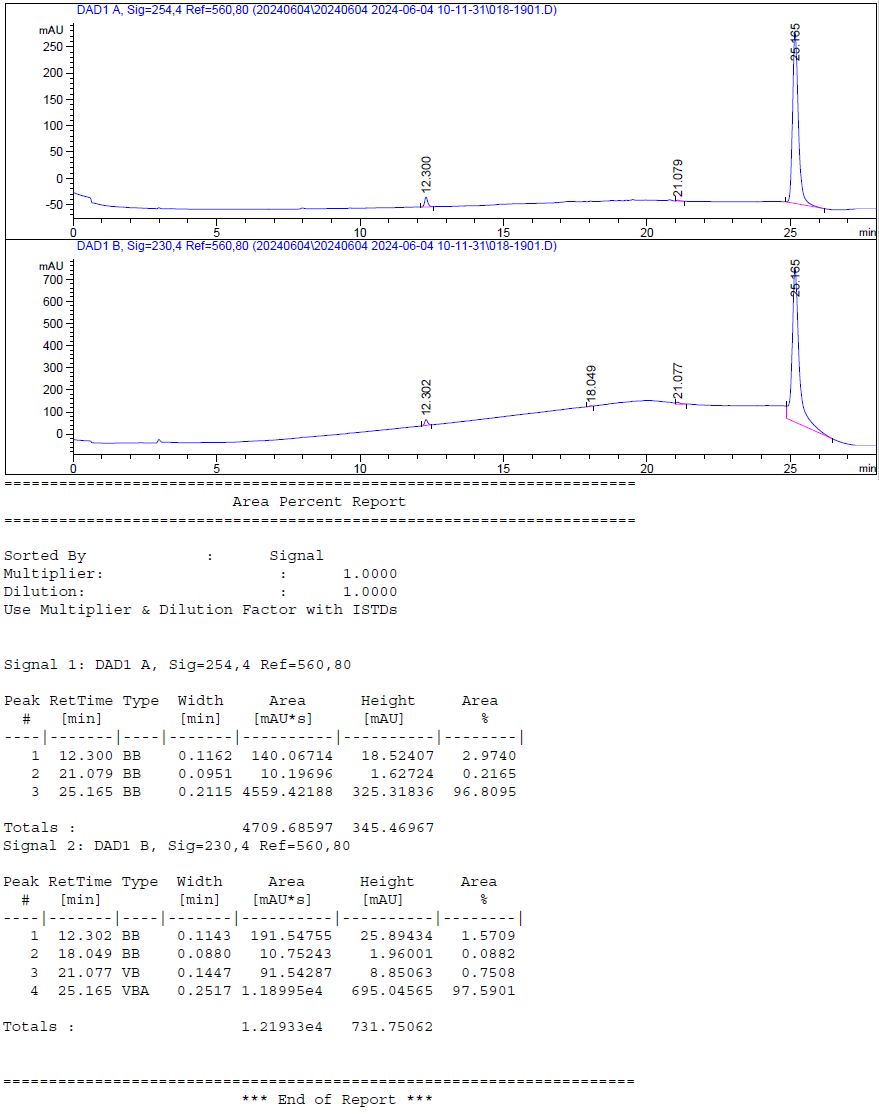


**Figure S1.48:** HPLC traces of compound **8g**.

**7h**

**Figure S1.49:** ^1^H NMR (400 MHz, CDCl_3_) **7h**.


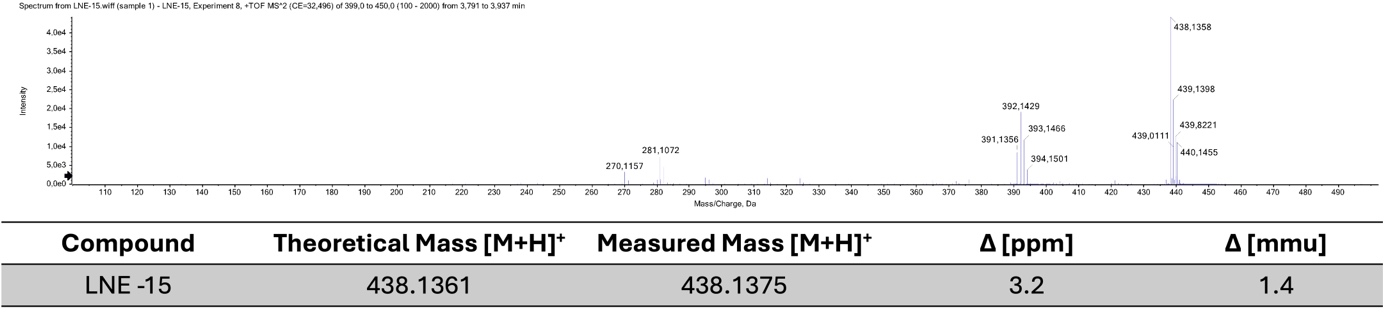


**Figure S1.50:** SWATH-MS results of **7h**.


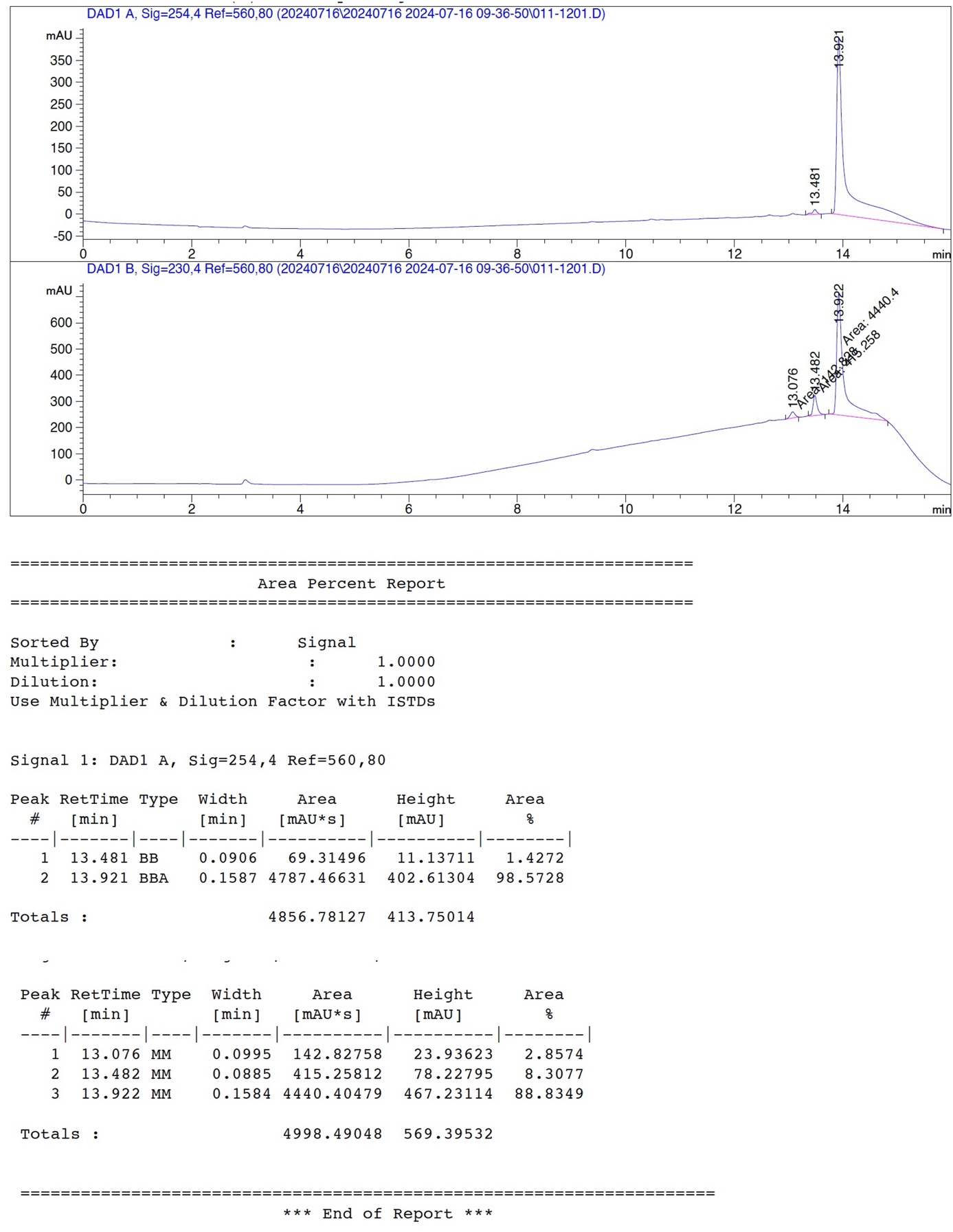


**Figure S1.51:** HPLC traces of compound **7h**.

**8h**

******

**Figure S1.52:** ^1^H NMR (400 MHz, CDCl_3_) **8h**.


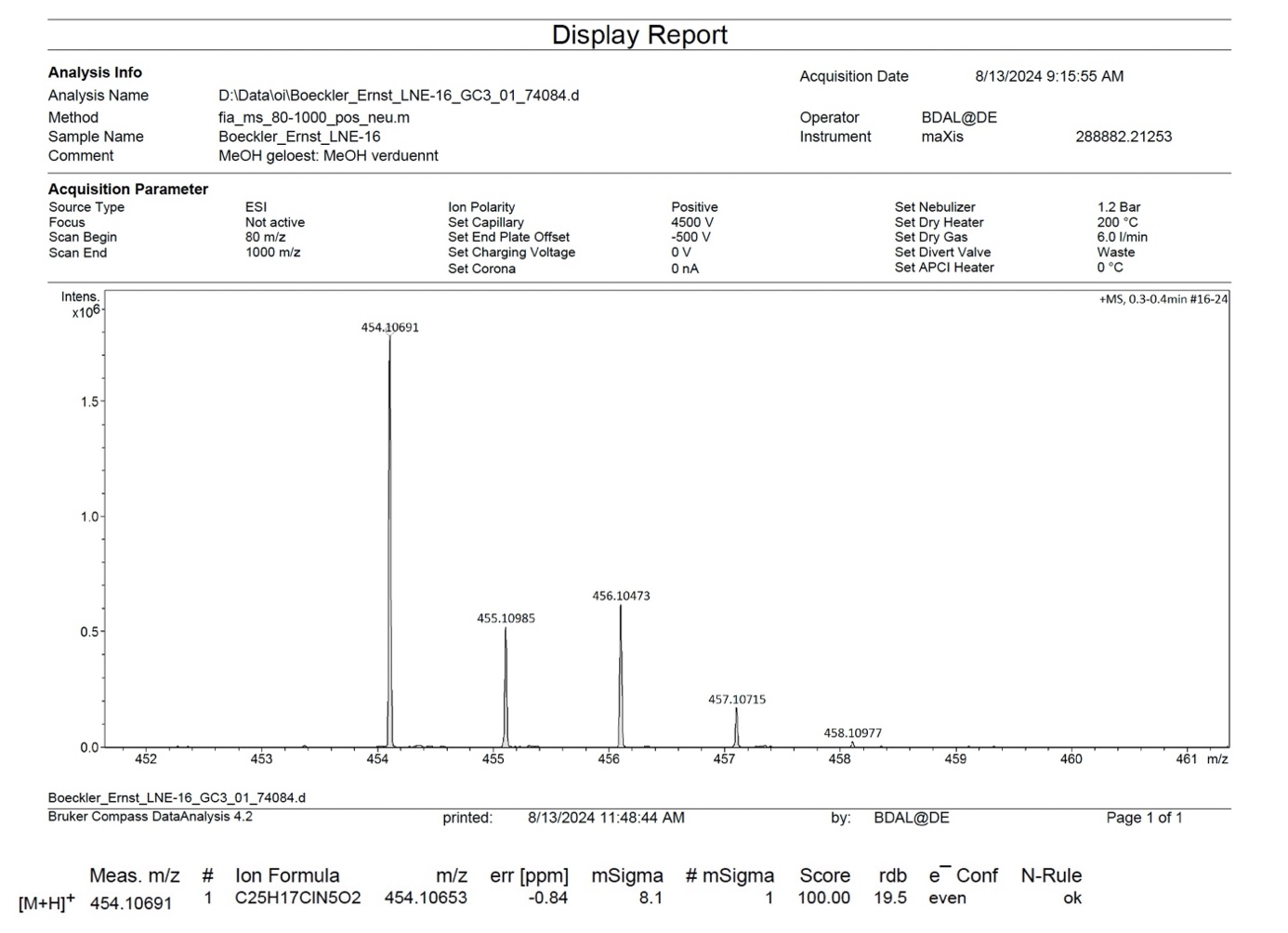


**Figure S1.53:** HRMS ESI-TOF results of **8h**.


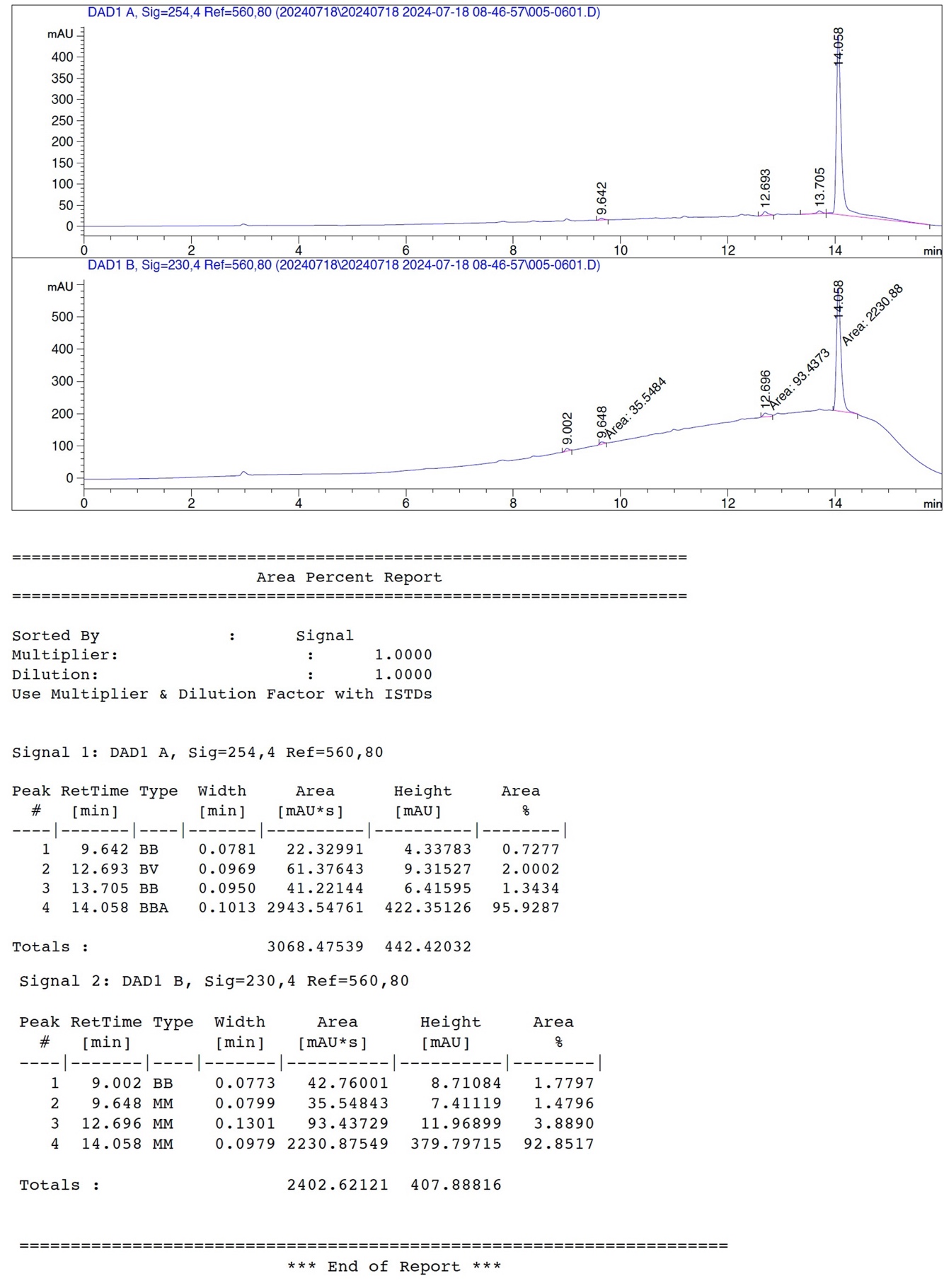


**Figure S1.54:** HPLC traces of compound **8h**.

**7i**

**Figure S1.55:** ^1^H NMR (400 MHz, CDCl_3_) **7i**.


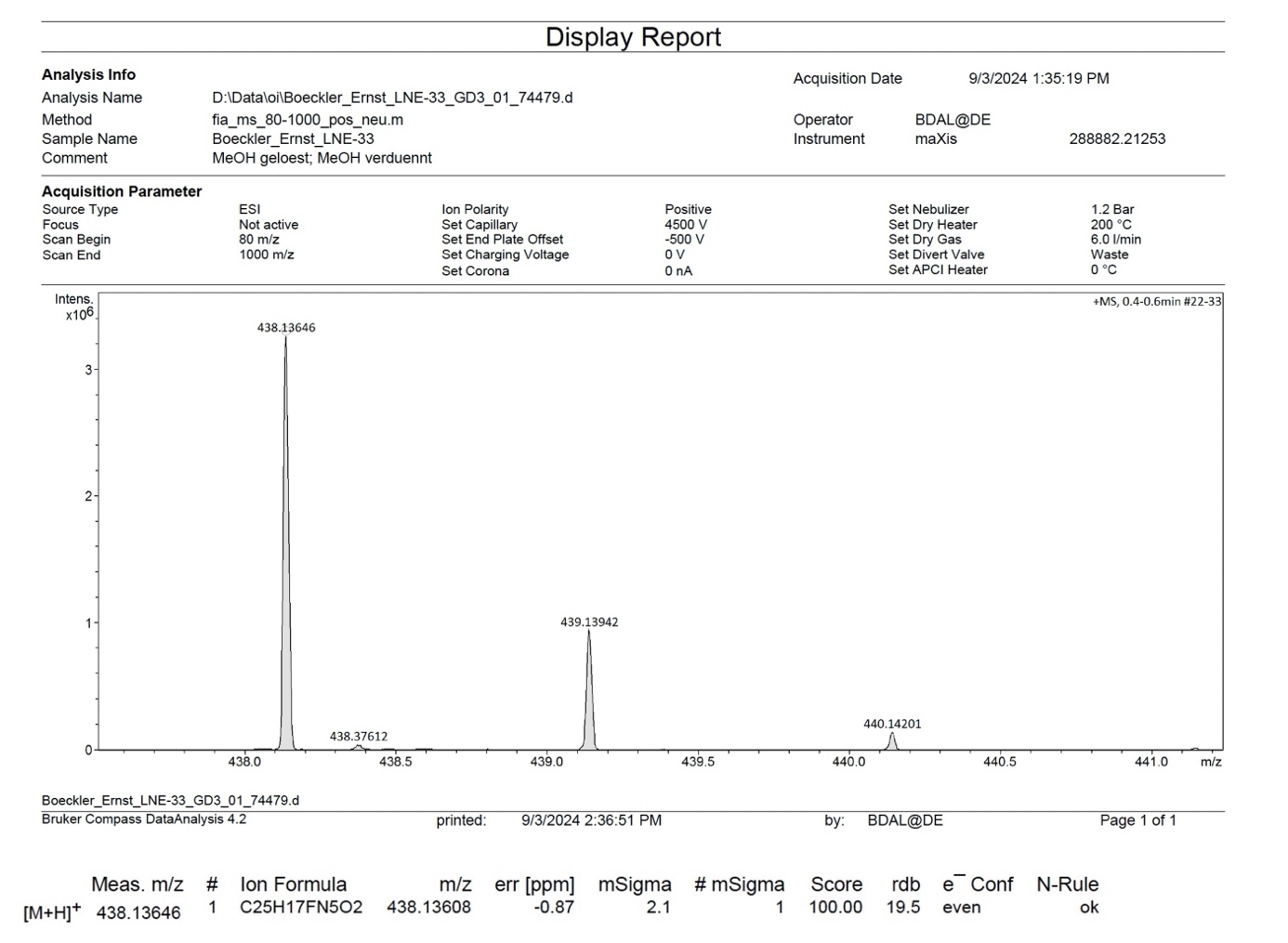


**Figure S1.56:** HRMS ESI-TOF results of **7i**.


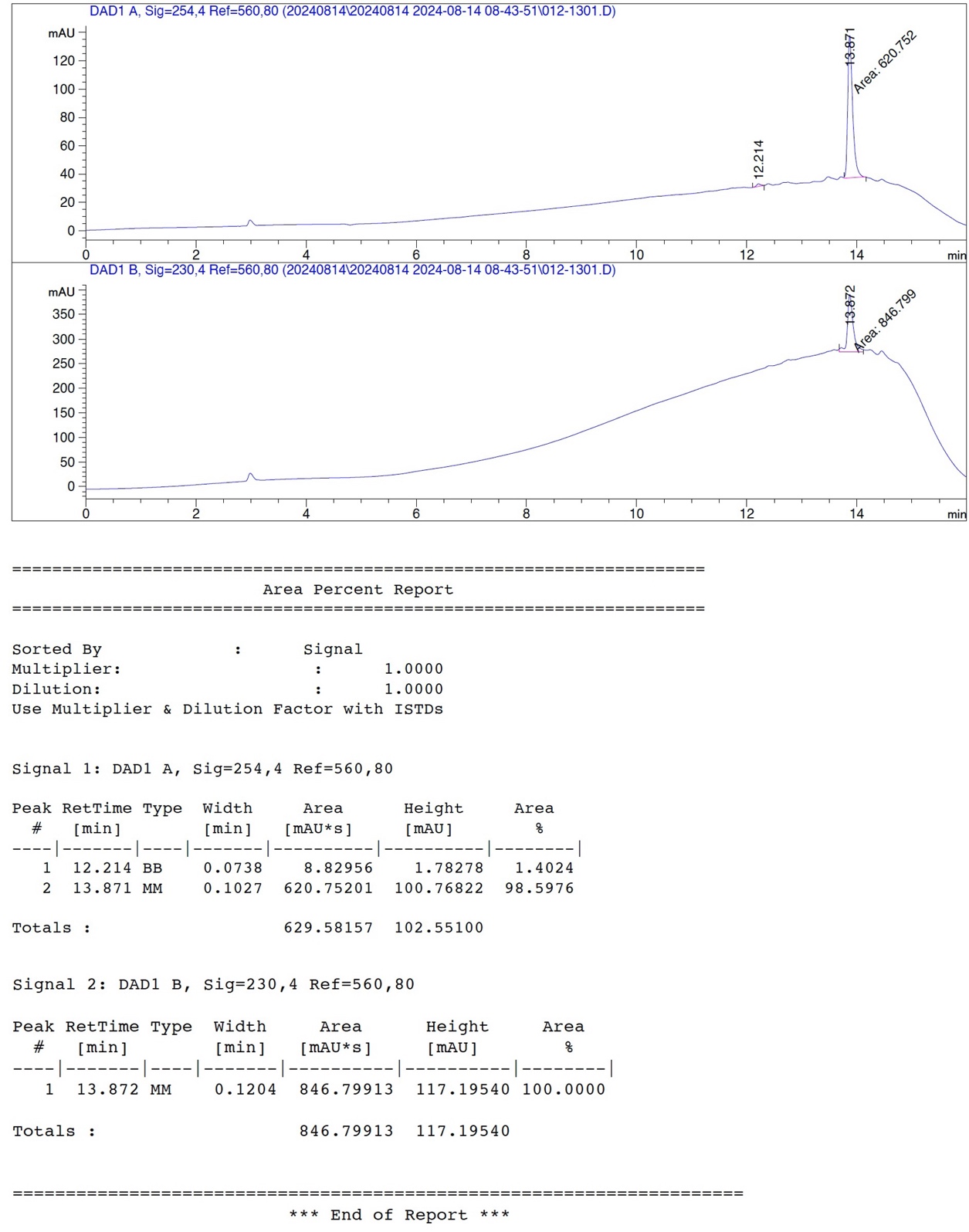


**Figure S1.57:** HPLC traces of compound **7i**.

**8i**

**Figure S1.58:** ^1^H NMR (400 MHz, CDCl_3_) **8i**.


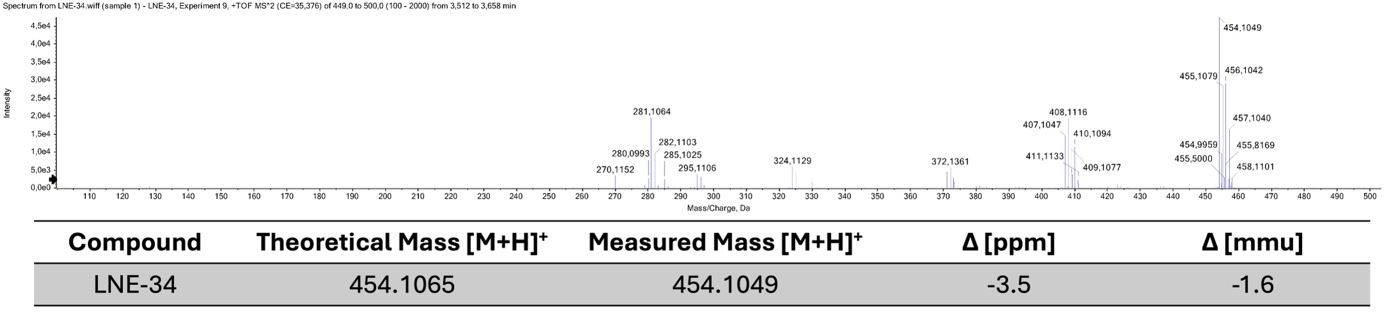


**Figure S1.59:** SWATH-MS results of **8i**.


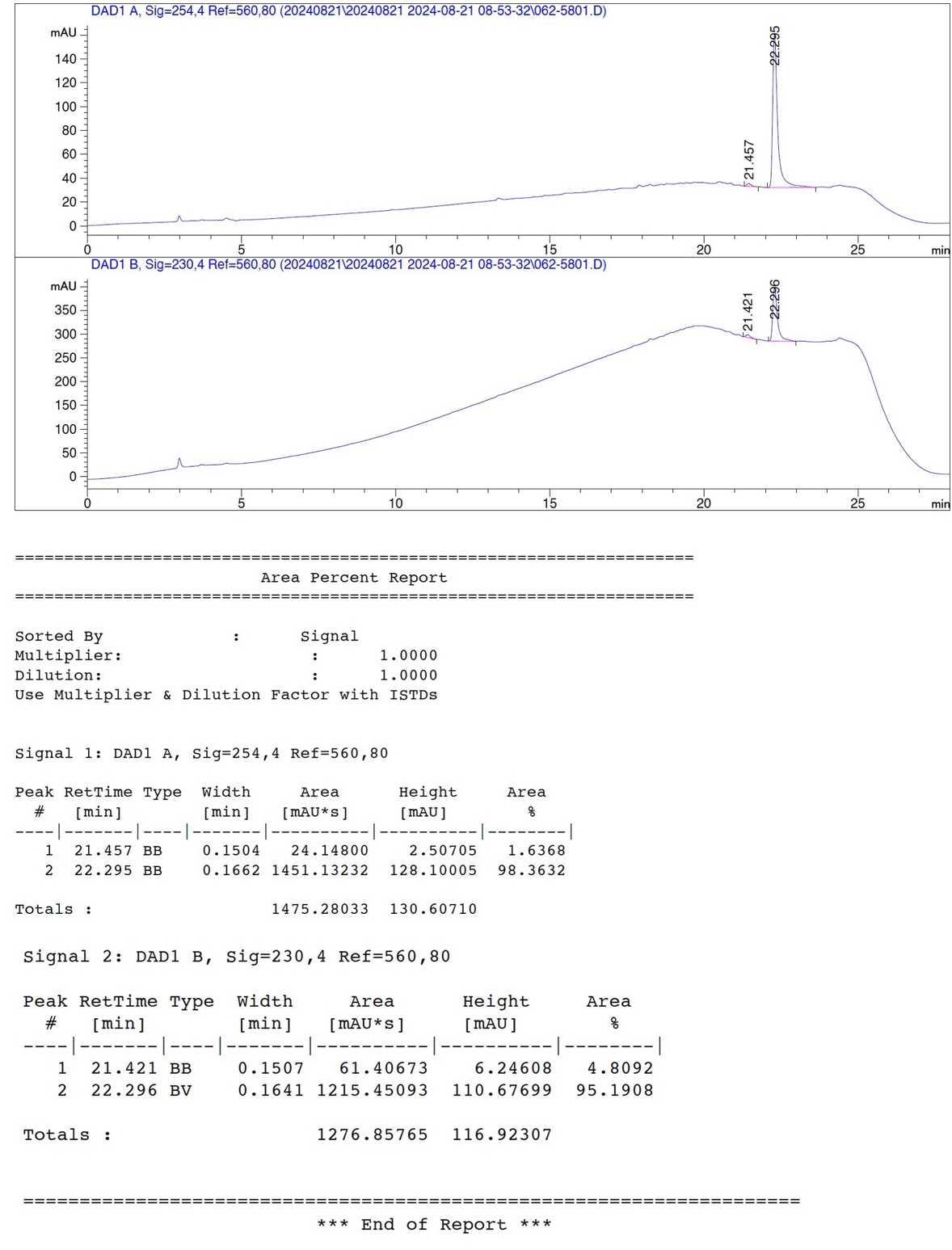


**Figure S1.60:** HPLC traces of compound **8i**.

# Reactivity measurements with Glutathione (GSH)

**Table S2.1:** Results of the reactivity GSH assay assessment of the synthesized SAR compounds and the original compounds **7a** and **8a** (100 µM) with 5 mM GSH. Each measurement was performed in duplicate

| **Compound ID** | **t_1/2_ GSH ± SD [h]** |
| --- | --- |
| **Afatinib** | 15.10 ± 1.01 |
| **7a** | 34.51 ± 1.59 |
| **8a** | > 100 |
| **7b** | 35.82 ± 10.06 |
| **8b** | > 100 |
| **7c** | 50.14 ± 4.16 |
| **8c** | > 100 |
| **7d** | 62.14 ± 10.18 |
| **8d** | > 100 |
| **7e** | N/A^[a]^ |
| **8e** | 11.07 ± 2.63 |
| **7f** | 18.46 ± 1.91 |
| **7g** | 24.76 ± 5.01 |
| **8g** | 17.70 ± 3.84 |
| **7h** | 18.58 ± 2.96 |
| **8h** | > 100 |
| **7i** | 39.05 ± 9.09 |
| **8i** | > 100 |

^[a]^No evaluable melting data points were obtained due to solubility issues


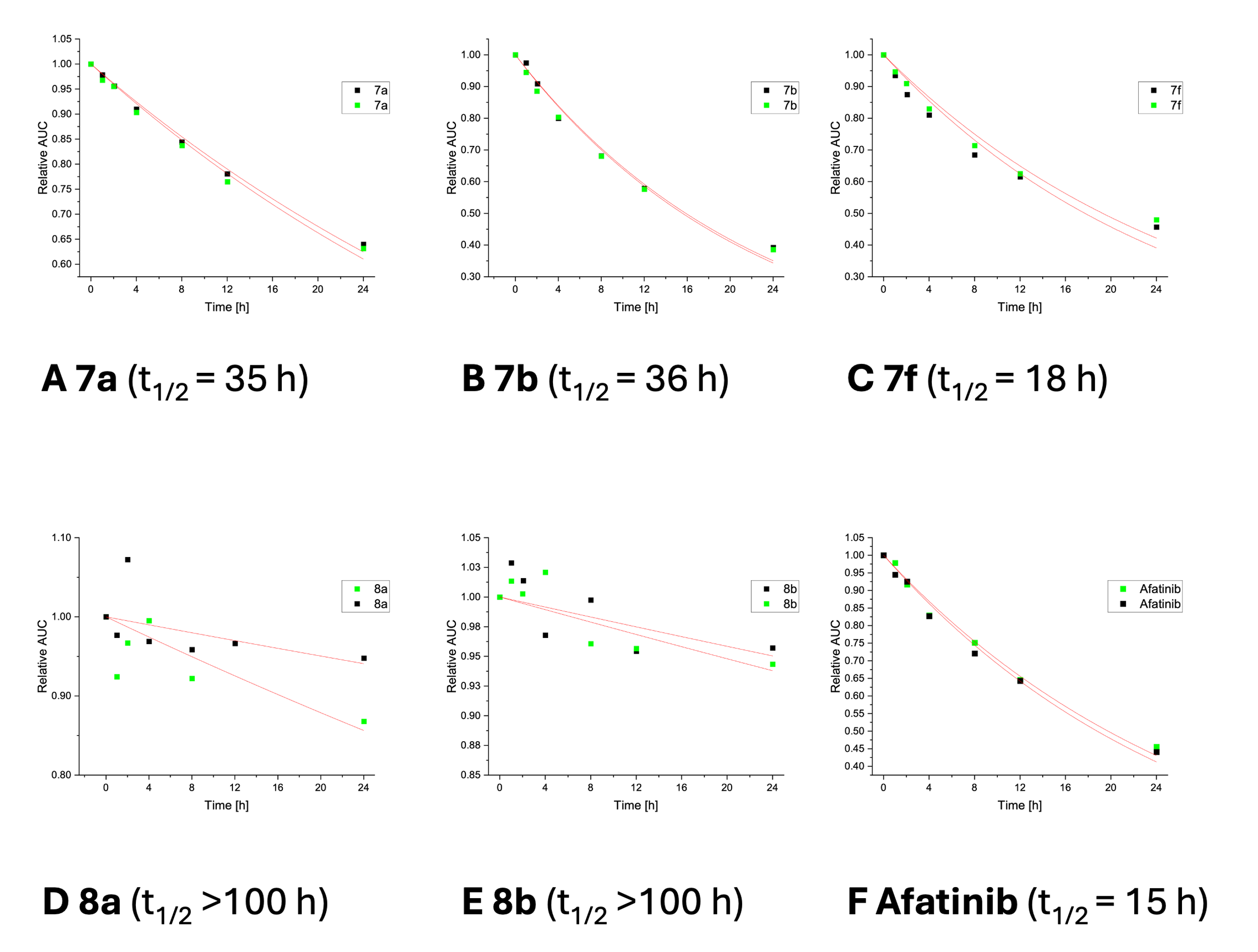


**Figure S2.1:** GSH assay results of (A) **7a**, (B) **7b**, (C) **7f**, (D) **8a**, (E)**8b** and (F) **Afatinib**.

**
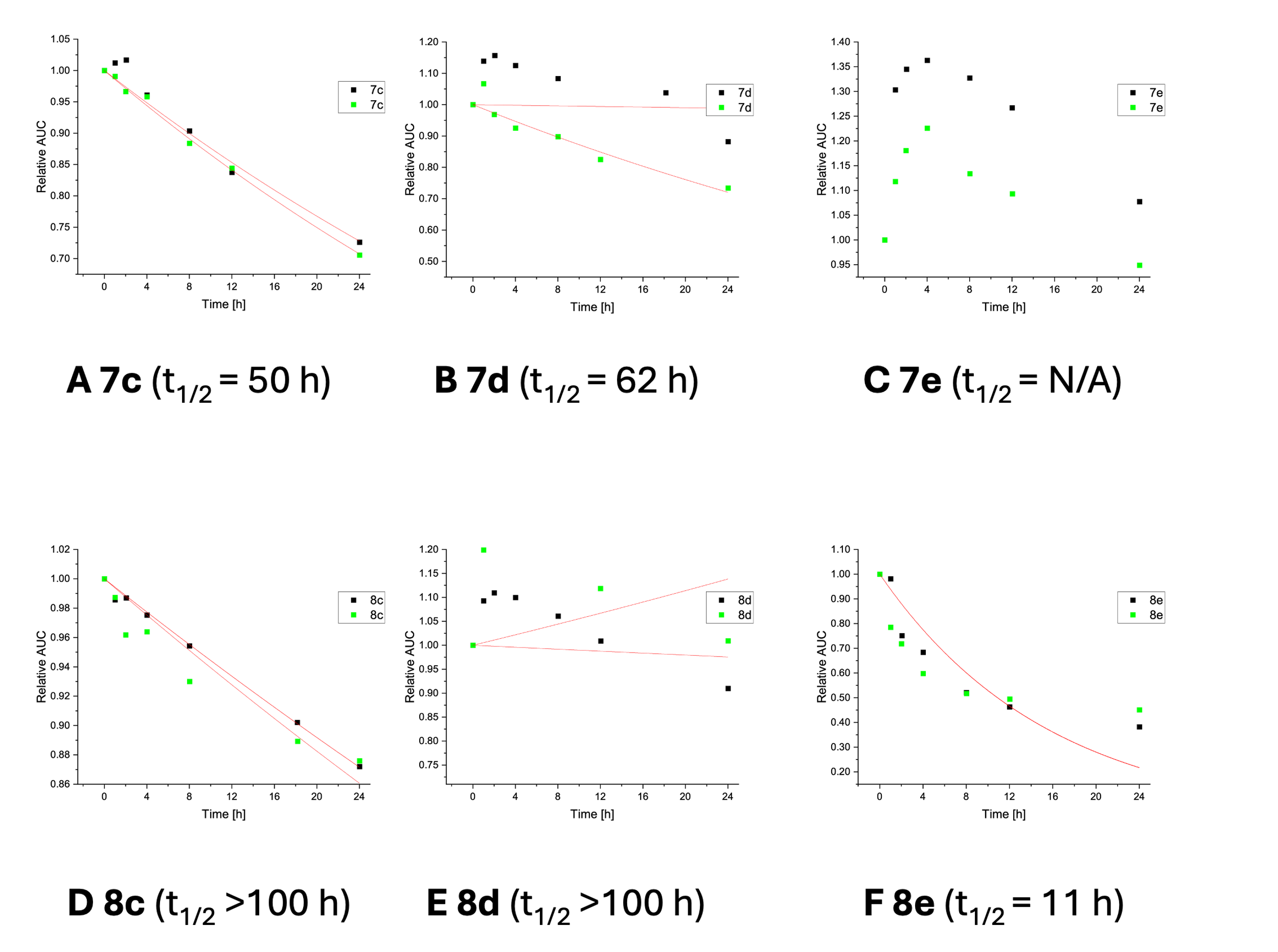
Figure S2.2:** GSH assay results of (A) **7c**, (B) **7d**, (C) **7e**, (D) **8c**, (E)**8d** and (F) **8e**.

**
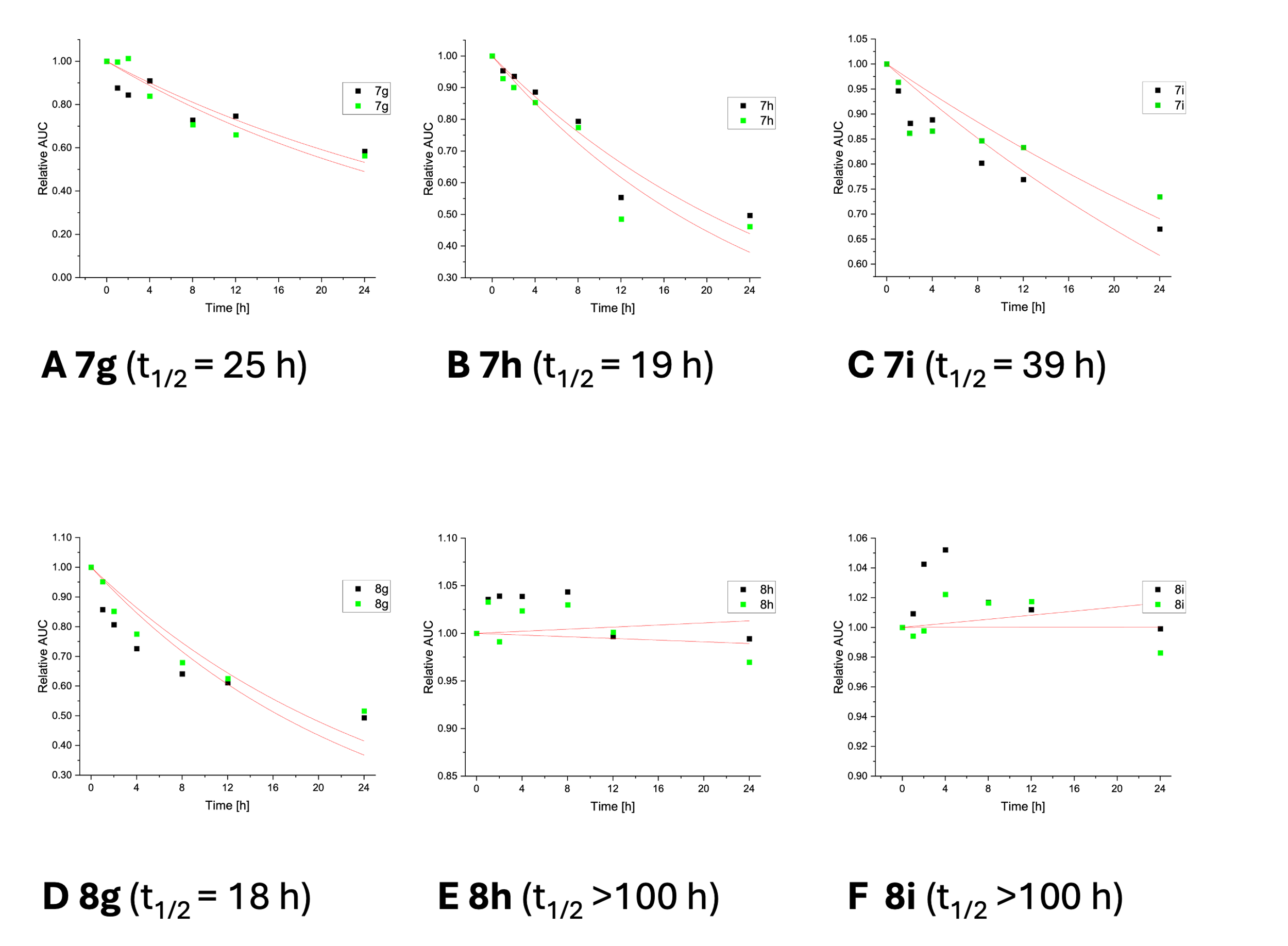
Figure S2.3:** GSH assay results of (A) **7g**, (B) **7h**, (C) **7i**, (D) **8g**, (E)**8h** and (F) **8i**.

# X-ray Crystallography

**Determination of the Binding Mode of Compound GCL36 using X-ray Crystallography**

**Table S3.1:** Crystallographic data collection and refinement statistics of the dataset of USP7 co-crystallised with GCL36 corresponding to the PDB code 9QJE. Parenthesized values show the respective value of the highest resolution bin.

|  | **Compound 7a (PDB: 9QJE)** |
| --- | --- |
| Wavelength [Å] | 1.03 |
| Space group | P 2_1_ |
| Cell dimension  a, b, c [Å]  α, ß, γ [°] | 75.75, 68.82, 77.67  90.00, 94.48, 90.00 |
| Resolution range [Å] | 50-2.26 (2.40-2.26) |
| Redundancy | 7.06 (7.31) |
| Completeness [%] | 98.90 (99.20) |
| Mean I/σ(I) | 23.33 (1.02) |
| R-meas [%] | 4.10 (171.50) |
| CC1/2 [%] | 100.00 (59.90) |
| Wilson B [Å²] | 78.21 |
| Resolution included [Å] | 43.58-2.26 |
| R_work_/R_free_ [%] | 20.36/24.78 |
| Bond RMSD [Å] | 0.008 |
| Angle RMSD [°] | 0.891 |
| Ramachandran [%] (favored/allowed/outliers) | 97.74/2.26/0.00 |
| Rotamer outliers [%] | 5.60 |
| All-atom Clashscore | 3.29 |
| Average B factor [Å^2^]  Overall  Protein  Ligand  Water | 90  90  86  109 |

Compound **7a** was modeled with 86 % (chain A) and 84 % (chain B) occupancy. We acknowledge that the compound is close to a symmetry contact and near a pseudo two fold rotation axis (Figure S3.1). However, due to the lack of full occupancy and a slight structural rearrangement at an adjacent helix we believe that this pose is not driven by these contacts. A bromine atom was modeled into the density, where Leu288 packs in the apo crystal (our unpublished apo dataset matches the packing of our search model 4M5X), as this fit the density best.


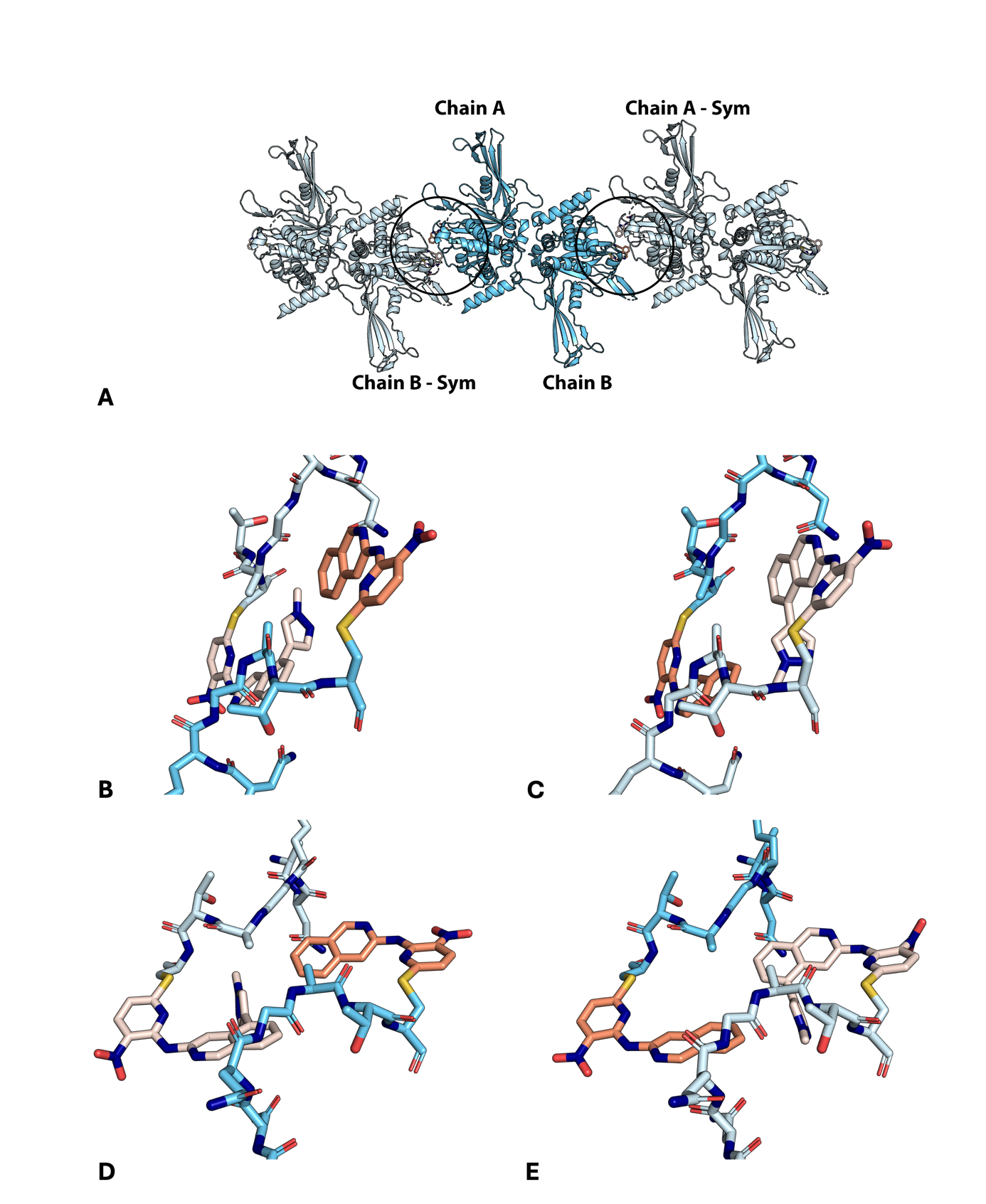


**Figure S3.1**: Overview of the symmetry contacts (A) Chain A is in proximity of a symmetry mate of chain B, while chain B is in proximity of a symmetry mate of chain A. (B) and (C) show the contact of chain A (blue) and the symmetry related chain B (light blue) from the same perspective. (D) and (E) show the contact of chain A (blue) and the symmetry related chain B (light blue) from the same perspective.

# Protein Sequences

**Table S4.1:** All used protein sequences

| Protein | Sequence (N’-C’) |
| --- | --- |
| USP7 | GGSKKHTGYVGLKNQGATCYMNSLLQTLFFTNQLRKAVYMMPTEGDDSSKSVPLALQRVFYELQHSDKPVGTKKLTKSFGWETLDSFMQHDVQELCRVLLDNVENKMKGTCVEGTIPKLFRGKMVSYIQCKEVDYRSDRREDYYDIQLSIKGKKNIFESFVDYVAVEQLDGDNKYDAGEHGLQEAEKGVKFLTLPPVLHLQLMRFMYDPQTDQNIKINDRFEFPEQLPLDEFLQKTDPKDPANYILHAVLVHSGDNHGGHYVVYLNPKGDGKWCKFDDDVVSRCTKEEAIEHNYGGHDDDLSVRHCTNAYMLVYIRESKLSEVLQAVTDHDIPQQLVERLQEEKRIEAQKRKERQE |
| USP7asoc | GGSKKHTGYVGLKNQGATCYMNSLLQTLFFTNQLRKAVYMMPTEGDDSSKSVPLALQRVFYELQHSDKPVGTKKLTKSFGWETLDSFMQHDVQELSRVLLDNVENKMKGTSVEGTIPKLFRGKMVSYIQSKEVDYRSDRREDYYDIQLSIKGKKNIFESFVDYVAVEQLDGDNKYDAGEHGLQEAEKGVKFLTLPPVLHLQLMRFMYDPQTDQNIKINDRFEFPEQLPLDEFLQKTDPKDPANYILHAVLVHSGDNHGGHYVVYLNPKGDGKWSKFDDDVVSRSTKEEAIEHNYGGHDDDLSVRHSTNAYMLVYIRESKLSEVLQAVTDHDIPQQLVERLQEEKRIEAQKRKERQE |

# Intact Protein Mass Spectrometry


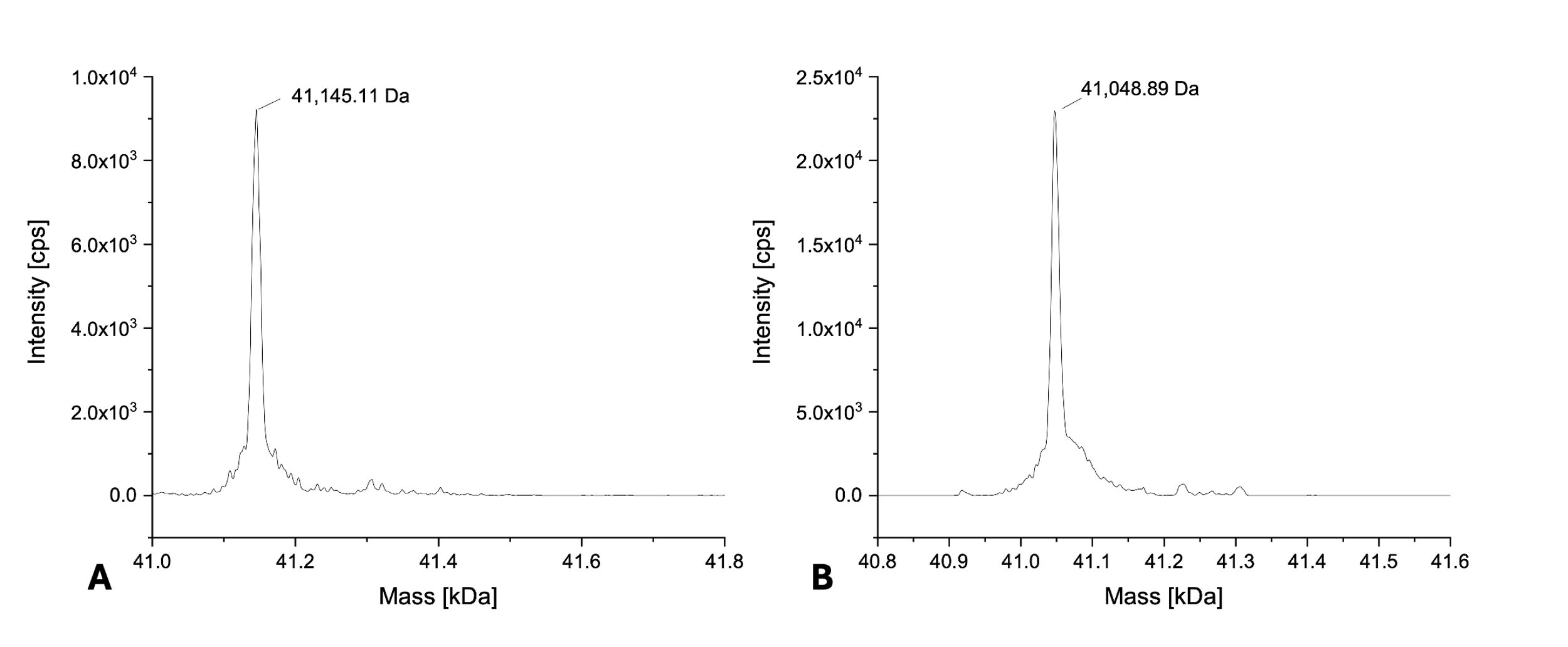


**Figure S5.1**: Deconvoluted MS spectra of the intact USP7 (A) and USP7asoc mutant (B).

**Table S5.1:** Results of the USP7cd proteins of the MS experiment

| **Protein** | **Theoretical mass [Da]** | **Experimental mass [Da]** | **∆m [Da]** | **∆m [ppm]** |
| --- | --- | --- | --- | --- |
| USP7 | 41,145.61 | 41,145.11 | -0.50 | -12.2 |
| USP7asoc | 41,049.22 | 41,048.89 | -0.33 | -8.0 |
